# Harnessing unprotected deactivated amines and arylglyoxals in the Ugi reaction for the synthesis of fused complex nitrogen heterocycles

**DOI:** 10.3762/bjoc.20.154

**Published:** 2024-07-25

**Authors:** Javier Gómez-Ayuso, Pablo Pertejo, Tomás Hermosilla, Israel Carreira-Barral, Roberto Quesada, María García-Valverde

**Affiliations:** 1 Departamento de Química, Universidad de Burgos, Burgos 09001, Spain

**Keywords:** arylglyoxals, deactivated amines, nitrogen heterocycles, Ugi reaction

## Abstract

Piperazines and diazepines are examples of nitrogen heterocycles present in many marketed drugs highlighting their importance in the discovery of novel bioactive compounds. However, their synthesis often faces challenges, including complex functionalization and lengthy reaction sequences. Multicomponent reactions, notably the Ugi reaction, have emerged as powerful tools to address these hurdles. Here, we have demonstrated the possibility of using the combination of arylglyoxals and carboxylic acids tethered to nonprotected deactivated amines as a powerful strategy for the synthesis of complex fused heterocycles. The limited nucleophilic character of the amino group of the anthranilic acid, indole-2-carboxylic acid, pyrrole-2-carboxylic acid or *N*-phenylglycine has allowed the use of these compounds in the Ugi reaction without triggering competitive reactions. The additional functional group present in the resulting Ugi adduct can be leveraged in different post-condensation strategies to easily generate multiple fused nitrogen heterocycles including benzodiazepinone and piperazinone cores.

## Introduction

**Figure 1:**
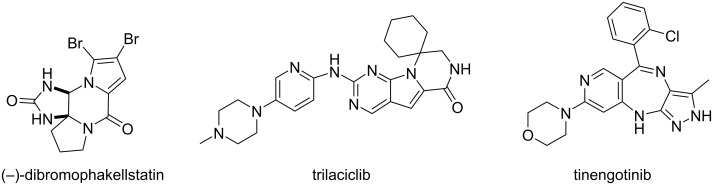
Fused heterocycles containing the piperazine and diazepine core.

Nitrogen heterocycles such as piperazines or diazepines represent important systems in the search of new bioactive compounds, as evidenced by the fact that they are present in many marketed drugs [1–2]. These substructures are found fused with other heterocycles in many cases, as illustrated by the antineoplastic dibromophakellstatin [3–6], the CDK inhibitor trilaciclib [7] or the kinase inhibitor tinengotinib [8] (Figure 1). Despite the interest of these structures, several drawbacks are typically found during their syntheses, for instance the difficulty in obtaining certain functionalizations or the need of long, elaborated reaction sequences.

In recent years, multicomponent reactions have become a useful tool for the synthesis of complex structures which overcome many of these problems, with the Ugi reaction leading these strategies. The scope of the Ugi reaction further increases when coupled with post-condensation reactions, which usually require the use of starting reagents that bear two functional groups. In order to avoid competitive reactions, these groups need to be compatible with the components of the Ugi reaction or the intermediates generated during the reaction. Thus, an amine can rarely be directly incorporated as an additional functional group, because of different competitive reactions triggered by it, such as the interrupted Ugi reaction resulting from the competitive addition over the nitrilium intermediate [9–10] or the split-Ugi reaction arising from a competitive *O*,*N*-acyl transference on the imidate intermediate, through a remote Mumm rearrangement [11].

Different strategies have been developed to avoid these competitive reactions, the most common ones being the use of protecting groups (Ugi/deprotection/cyclization strategy) [12–14] or of surrogates of amines [15]. However, direct incorporation of the second amine without derivatization is the most desirable strategy from the point-of view of sustainability. This has been achieved following two different approaches: the blockage of the amine group throughout the reaction by the incorporation of an additional group on the carbonyl component [16–18] or the use of amine groups with a limited reactivity, modulated by their substitution [19].

## Results and Discussion

### Synthesis of 3*H-*benzo[*e*][1,4]diazepin-5-ones

As part of our continued interest in developing new and efficient strategies for synthesizing complex fused nitrogen heterocycles, we decided to evaluate the use of amine groups with a reduced reactivity in the synthesis of 3*H-*benzo[*e*][1,4]diazepin-5-ones, heterocycles previously synthesized by our research group through Ugi/Staudinger/aza-Wittig and Ugi/reduction/cyclization sequences, using 2-azidobenzoic [20] and 2-nitrobenzoic [21] acids, respectively. The first methodology has several drawbacks, as the need to synthesise the 2-azidobenzoic acid from anthranilic acid and sodium azide, the requirement of anhydrous conditions to perform the Staudinger/aza-Wittig sequence or the generation of large quantities of triphenylphosphine oxide which needs to be removed by chromatography column, while the more eco-friendly second strategy needs an additional stage for the reduction of the nitro group on the Ugi adduct.

**Scheme 1:**
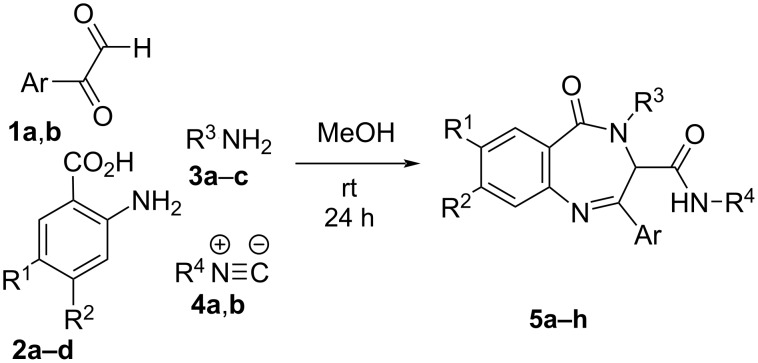
Synthesis of benzodiazepinones **5** from anthranilic acid derivatives.

In order to find a more efficient synthesis, we thought that the second nitrogen in the diazepine nucleus could be incorporated without the need of surrogates or protecting groups, as the reduced nucleophilic character of the amino group of the anthranilic acid would prevent its participation in the Ugi reaction. In this way, the Ugi reaction was performed using arylglyoxals **1**, anthranilic acid derivatives **2**, amines **3** and isocyanides **4** following the most common procedure. Thus, initially the amine **3** (1 equiv) was added to a solution of the arylglyoxal **1** (1 equiv) in methanol and the mixture was stirred for 15 min. Then, the acid **2** (1 equiv) and the isocyanide **4** (1 equiv) were added and the reaction was stirred at room temperature for 24 h. Fortunately, after the workup the only product observed was the corresponding benzodiazepinone **5**, resulting from a spontaneous cyclization of the Ugi adduct, in a six-center four-component Ugi reaction (U-6C-4CR), which prevents the need of additional steps (Scheme 1, Table 1).

**Table 1:** Results obtained in the synthesis of benzodiazepinones **5** from anthranilic acid derivatives.

Entry	**1** (Ar)	**2** (R^1^, R^2^)	**3** (R^3^)	**4** (R^4^)	**5** (%)^a^

1	**1a** (C_6_H_5_)	**2a** (H, H)	**3a** (C_6_H_5_CH_2_)	**4a** (*c*C_6_H_11_)	**5a** (66)^b^
2	**1a** (C_6_H_5_)	**2a** (H, H)	**3b** (2-NO_2_C_6_H_4_CH_2_)	**4a** (*c*C_6_H_11_)	**5b** (80)
3	**1b** (4-CH_3_C_6_H_4_)	**2a** (H, H)	**3c** (CH_2_CH_2_CH_2_Br)	**4a** (*c*C_6_H_11_)	**5c** (58)^c^
4	**1a** (C_6_H_5_)	**2a** (H, H)	**3a** (C_6_H_5_CH_2_)	**4b** (C_6_H_5_CH_2_)	**5d** (63)
5	**1a** (C_6_H_5_)	**2b** (H, NO_2_)	**3a** (C_6_H_5_CH_2_)	**4a** (*c*C_6_H_11_)	**5e** (68)
6	**1a** (C_6_H_5_)	**2c** (NO_2_, H)	**3a** (C_6_H_5_CH_2_)	**4a** (*c*C_6_H_11_)	**5f** (63)
7	**1a** (C_6_H_5_)	**2d** (I, H)	**3a** (C_6_H_5_CH_2_)	**4a** (*c*C_6_H_11_)	**5g** (67)
8	**1a** (C_6_H_5_)	**2d** (I, H)	**3b** (2-NO_2_C_6_H_4_CH_2_)	**4a** (*c*C_6_H_11_)	**5h** (61)

^a^Yield after purification; ^b^global yield for the synthesis of **5a** from Ugi/Staudinger/aza-Wittig sequence 42% [20], and from Ugi/reduction/cyclization sequence 55% [21]; ^c^the formation of pyrrolobenzodiazepine **8** (35%) was observed (see Scheme 4).

**Scheme 2:**
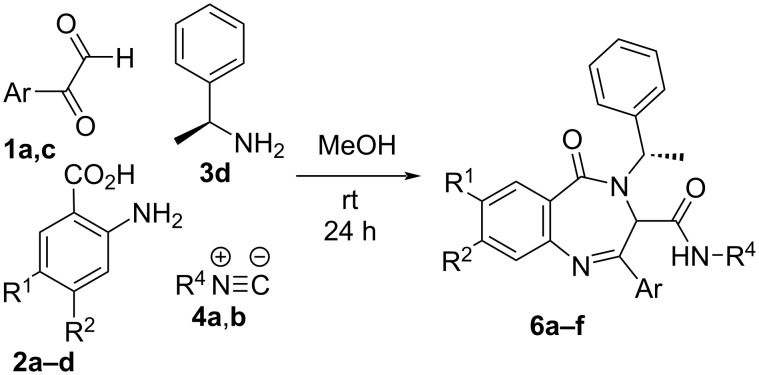
Diastereoselective one-pot synthesis of benzodiazepinones **6**.

Due to the interest of these results and considering the diastereoselectivity observed when enantiopure (*S*)-α-methylbenzylamine was used as chiral component in the two-step syntheses from amine surrogates [22], we assayed our new strategy to achieve a one-pot diastereoselective synthesis of benzodiazepinones **6** (Scheme 2, Table 2). Interestingly, the reaction took place with a α,3-*like* relative configuration on the major diastereomer, similar to that obtained for the Ugi/Staudinger/aza-Wittig sequence and complementary to that observed for the Ugi/reduction/cyclization sequence (see Supporting Information File 1, Figure S2).

**Table 2:** Results obtained in the diastereoselective one-pot synthesis of benzodiazepinones **6**.

Entry	**1** (Ar)	**2** (R^1^, R^2^)	**4** (R^4^)	**6** (%)^a^	dr^b^ (*l*:*u*)^c^

1	**1a** (C_6_H_5_)	**2a** (H, H)	**4a** (*c*C_6_H_11_)	**6a** (66)	79:21
2	**1a** (C_6_H_5_)	**2a** (H, H)	**4b** (C_6_H_5_CH_2_)	**6b** (80)	76:24
3	**1c** (4-FC_6_H_4_)	**2a** (H, H)	**4a** (*c*C_6_H_11_)	**6c** (58)	74:26
4	**1a** (C_6_H_5_)	**2b** (H, NO_2_)	**4a** (*c*C_6_H_11_)	**6d** (63)	72:28
5	**1a** (C_6_H_5_)	**2c** (NO_2_, H)	**4a** (*c*C_6_H_11_)	**6e** (63)	68:32
6	**1a** (C_6_H_5_)	**2d** (I, H)	**4a** (*c*C_6_H_11_)	**6f** (67)	76:24

^a^Yield referred to the major diastereomer after purification; ^b^determined by ^1^H NMR of the reaction mixture; ^c^configuration *l*(α*S*,3*S*):*u*(α*S*,3*R*) [22].

**Scheme 3:**
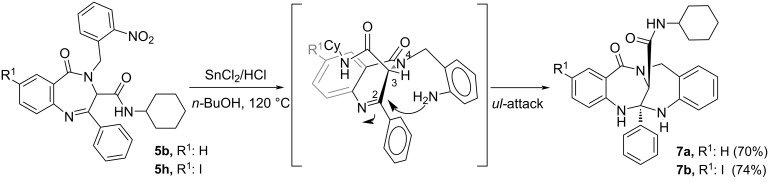
Synthesis of bis-1,4-benzodiazepines **7**.

With the aim of building more complex systems, we explored post-condensation reactions on the 2-nitrobenzylamine and 3-bromopropylamine derivatives. Thus, we carried out the reduction of the nitro group on derivatives **5b** and **5h** employing tin(II) chloride and chlorhydric acid in boiling *n*-butanol (120 °C), conditions previously assayed in our group. Under these conditions, the bis-1,4-benzodiazepines **7** resulting from the reaction of the amino group with the imine on the benzodiazepine were obtained as a single diastereomer. On the basis of the preferred conformation for the 1,4-benzodiazepin-5-ones **5** and **6** [20,22], where the amide substituent in C3 is pseudoaxially oriented in an all-*trans* conformation, only the *unlike* attack would be allowed (Scheme 3).

**Scheme 4:**
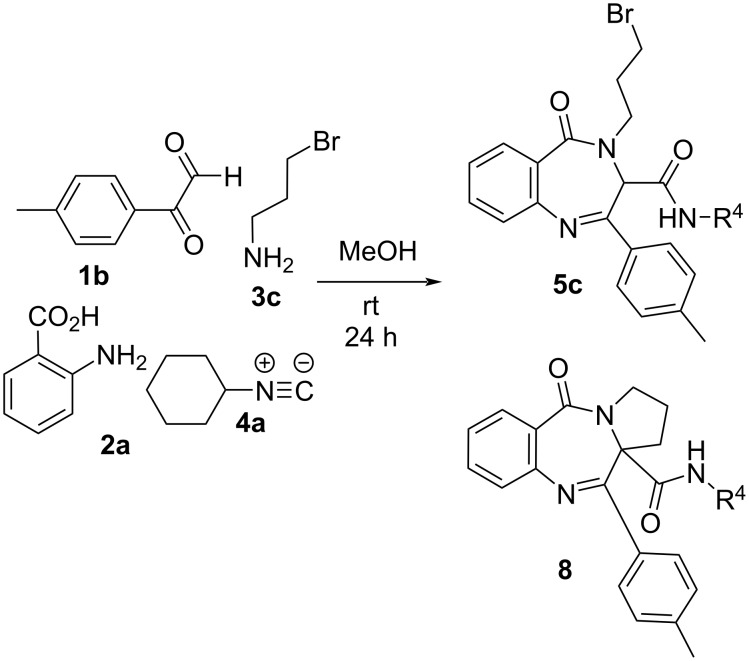
Synthesis of benzodiazepinone **5c** and pyrrolobenzodiazepinone **8** from anthranilic acid and 3-bromopropanamine.

On the other hand, when 3-bromopropanamine was used, the formation of the pyrrolobenzodiazepinone system **8** was observed albeit along with benzodiazepinone **5c** (Scheme 4). In order to improve the synthesis of this fused heterocycle, different strategies were tried, e.g., performing the Ugi reaction in different solvents (dichloromethane, ethyl acetate, methanol) to prevent the precipitation of benzodiazepine **5c**, conducting the Ugi reaction in boiling methanol or treating the benzodiazepine with different bases (caesium carbonate, sodium hydroxide). However, the complete cyclisation to the pyrrolobenzodiazepinone was not achieved, so the described three-step strategy starting from 2-nitrobenzoic acid (Ugi reaction/cyclization to pyrrolidine/reduction sequence) remains as a better choice [23].

### Synthesis of piperazinones

**Scheme 5:**
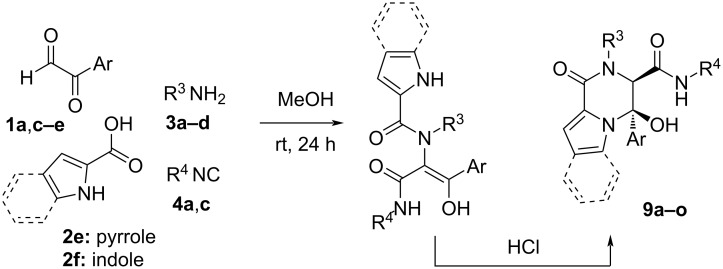
Synthesis of pyrrolopiperazinones **9** from pyrrole and indole carboxylic acids.

**Scheme 6:**
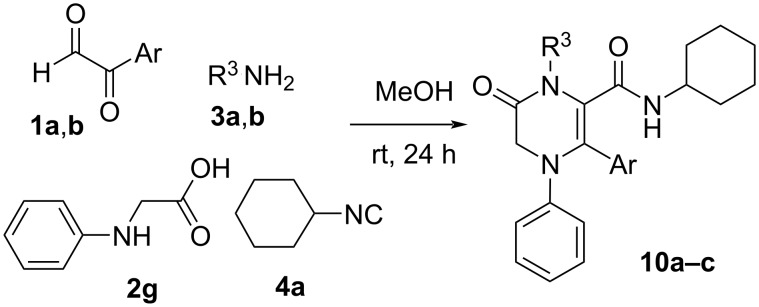
Synthesis of pyrrolopiperazinones **10** from *N*-phenylglicine.

**Figure 2:**
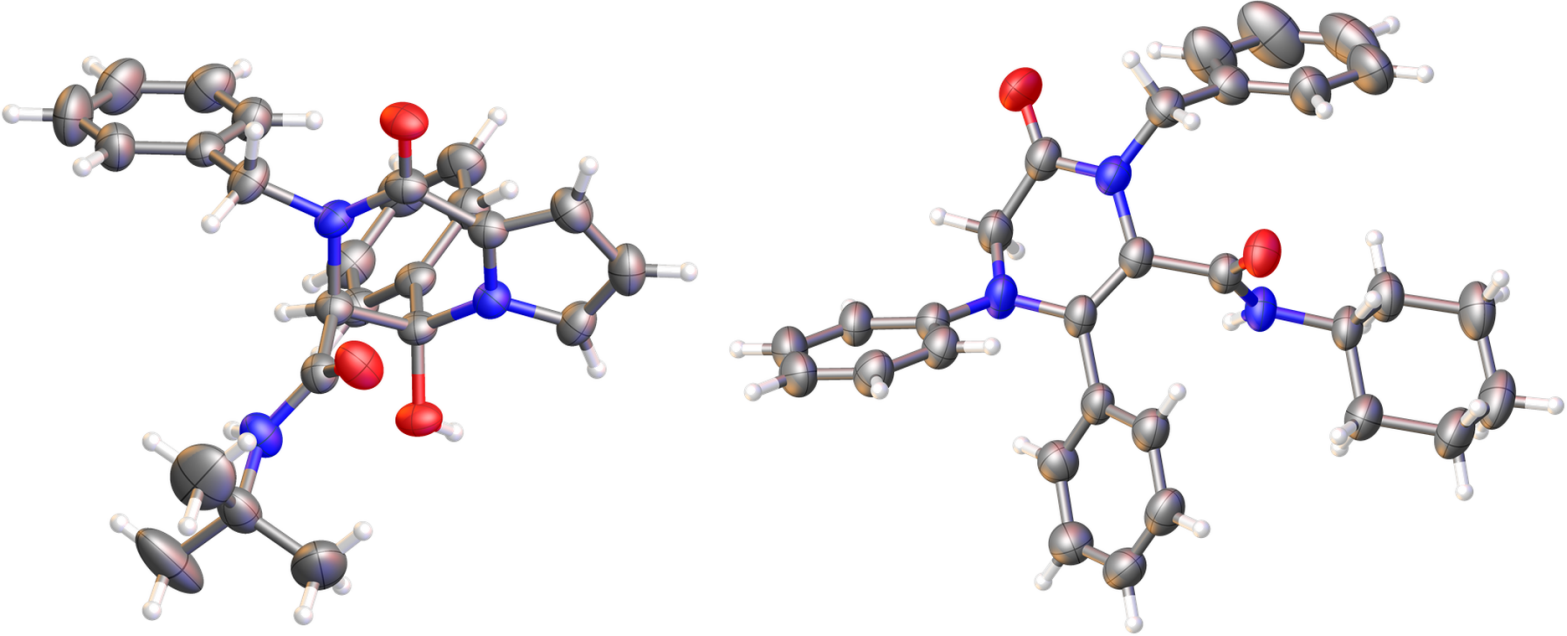
X-ray diffraction structures of pyrrolopiperazinones **9a** (left) and **10a** (right). The thermal ellipsoid plot (Olex2) is at the 40% probability level.

On the basis of these results, we planned to synthesise piperazinone derivatives following a similar strategy. Thus, we chose different deactivated 2-amino-substituted carboxylic acid derivatives such as the indole-2-carboxylic acid, recently used in a similar strategy with aromatic amines [24], pyrrole-2-carboxylic acid and *N*-phenylglycine, and carried out their reactions with arylglyoxals, alkylamines and isocyanides (Scheme 5). As we expected, the cyclization product **9** was observed, although the complete cyclization of the Ugi adduct, found as the enol tautomer, needed the addition of hydrochloric acid (1 equiv) in the case of the *tert-*butylamine derivatives. However, the nature of the cyclization product depended on the carboxylic acid employed in the Ugi reaction. Thus, the indole and pyrrole derivatives yielded the corresponding hemiaminal **9** as a single diastereomer (Scheme 5, Table 3), while *N*-phenylglycine afforded the enamine derivative **10** (Scheme 6, Table 4). Both structures were confirmed by single-crystal X-ray diffraction of compounds **9a** and **10a** (Figure 2). The diastereoselectivity observed in the hemiaminals and their stability can be explained by the intramolecular hydrogen bond formed between the hydroxy group and the carbamide substituent, which explains the high deshielding observed in the ^1^H NMR spectra for the signal of the OH group (around 8.5 ppm).

**Table 3:** Results obtained in the synthesis of pyrrolopiperazinones **9** from pyrrole and indole carboxylic acids.

Entry	**1** (Ar)	**2**	**3** (R^3^)	**4** (R^4^)	**9** (%)^a,b^

1	**1a** (C_6_H_5_)	**2e**	**3a** (C_6_H_5_CH_2_)	**4c** (C(CH_3_)_3_)	**9a** (61)^b^
2	**1a** (C_6_H_5_)	**2e**	**3d** (C(CH_3_)_3_)	**4a** (*c*C_6_H_11_)	**9b** (52)^c^
3	**1a** (C_6_H_5_)	**2e**	**3c** (CH_2_CH_2_CH_2_Br)	**4a** (*c*C_6_H_11_)	**9c** (16)^d^
4	**1a** (C_6_H_5_)	**2e**	**3c** (CH_2_CH_2_CH_2_Br)	**4a** (C(CH_3_)_3_)	**9d** (24)^d^
5	**1c** (4-FC_6_H_4_)	**2e**	**3c** (CH_2_CH_2_CH_2_Br)	**4c** (C(CH_3_)_3_)	**9e** (22)^d^
6	**1a** (C_6_H_5_)	**2e**	**3b** (2-NO_2_C_6_H_4_CH_2_)	**4a** (*c*C_6_H_11_)	**9f** (77)
7	**1d** (4-CF_3_C_6_H_4_)	**2e**	**3b** (2-NO_2_C_6_H_4_CH_2_)	**4c** (C(CH_3_)_3_)	**9g** (69)
8	**1a** (C_6_H_5_)	**2f**	**3a** (C_6_H_5_CH_2_)	**4a** (*c*C_6_H_11_)	**9h** (49)
9	**1c** (4-FC_6_H_4_)	**2f**	**3a** (C_6_H_5_CH_2_)	**4a** (*c*C_6_H_11_)	**9i** (58)
10	**1a** (C_6_H_5_)	**2f**	**3d** (C(CH_3_)_3_)	**4c** (C(CH_3_)_3_)	**9j** (65)^c^
11	**1a** (C_6_H_5_)	**2f**	**3c** (CH_2_CH_2_CH_2_Br)	**4c** (C(CH_3_)_3_)	**9k** (22)^d^
12	**1a** (C_6_H_5_)	**2f**	**3b** (2-NO_2_C_6_H_4_CH_2_)	**4a** (*c*C_6_H_11_)	**9l** (72)
13	**1c** (4-FC_6_H_4_)	**2f**	**3b** (2-NO_2_C_6_H_4_CH_2_)	**4a** (*c*C_6_H_11_)	**9m** (75)
14	**1e** (4-CH_3_OC_6_H_4_)	**2f**	**3b** (2-NO_2_C_6_H_4_CH_2_)	**4c** (C(CH_3_)_3_)	**9n** (76)
15	**1d** (4-CF_3_C_6_H_4_)	**2f**	**3b** (2-NO_2_C_6_H_4_CH_2_)	**4c** (C(CH_3_)_3_)	**9o** (71)

^a^Yield after purification; ^b^the only diastereomer observed was the (3R*,4R*); ^c^yield obtained after treatment of the reaction mixture with HCl (1 equiv) for 24 h; ^d^the corresponding dipyrrolopiperazinone **12** resulting from a second cyclization was isolated from the reaction mixture (**12a** (32%), **12b** (7%), **12c** (13%), **12d** (15%)) (see Scheme 8).

**Table 4:** Results obtained in the synthesis of pyrrolopiperazinones **10** from *N*-phenylglycine.

Entry	**1** (Ar)	**3** (R^3^)	**10** (%)^a^

1	**1a** (C_6_H_5_)	**3a** (C_6_H_5_CH_2_)	**10a** (54)
2	**1b** (4-FC_6_H_4_)	**3a** (C_6_H_5_CH_2_)	**10b** (49)
3	**1a** (C_6_H_5_)	**3b** (2-NO_2_C_6_H_4_CH_2_)	**10c** (−)^b,c^

^a^Yield after purification; ^b^unstable; ^c^used in the following step without purification.

**Scheme 7:**
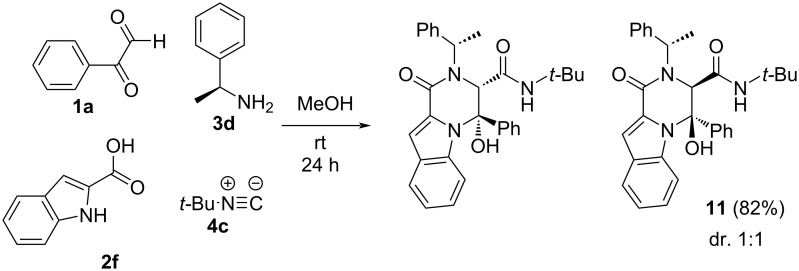
Synthesis of pyrrolopiperazinone **11** using (*S*)-α-methylbenzylamine.

As before, we also explored the stereochemical outcome in the synthesis of pyrrolopiperazinones when enantiopure (*S*)-α-methylbenzylamine was used as chiral component. In this case, although the relative configuration on C3,C4 remained unchanged ((3R*,4R*)), an equimolar mixture of diastereomers (2(1*S*),3*R*,4*R*) and (2(1*S*),3*S*,4*S*) was obtained (Scheme 7).

**Scheme 8:**
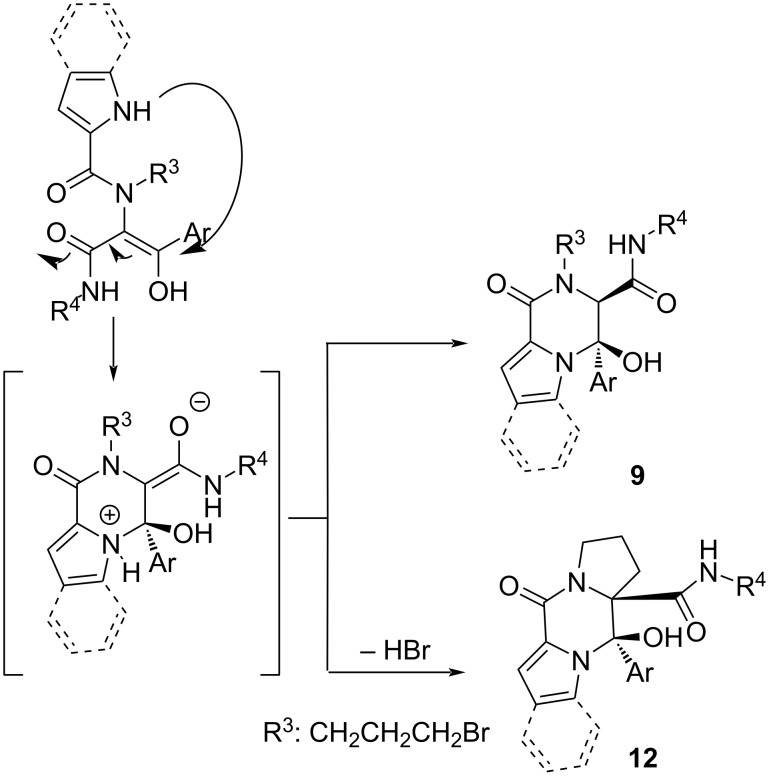
Proposed mechanism in the spontaneous cyclization of Ugi adducts obtained from arylglyoxals and deactivated amines.

These results indicate that these reactions seem to take place through conjugated additions on the enol tautomer of the Ugi adduct. Indeed, the enolate intermediate would explain the stereochemical results, controlled by the configuration in the hemiaminal intermediate and not by the chiral information on the amine, unlike in the case of benzodiazepinones, where the protonation would be exclusively controlled by the chiral amine in the synthesis of **6**. Moreover, this proposed mechanism also explains the spontaneous cyclization in the absence of a base when 3-bromopropanamine was used (Scheme 8).

Moreover, we envisaged the possibility of obtaining piperazinoquinazoline derivatives, a core found in fungal metabolites [25–26], systems with promising antitumour activity [27], as well as dipyrrolopiperazinone derivatives, substructure found in some alkaloids such as dibromophakellin or the palau’amine [28], which possess immunosuppressive and cytotoxic properties [29], through post-condensation reactions.

**Scheme 9:**
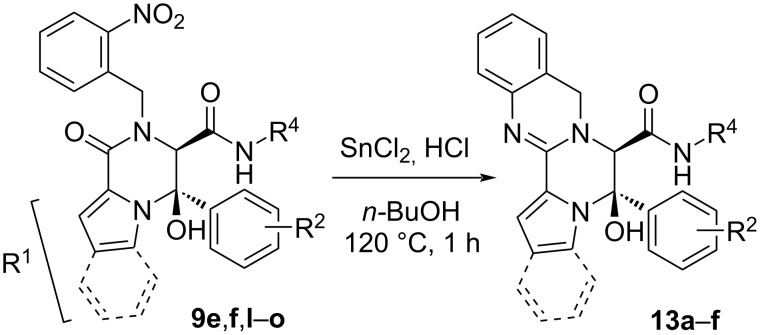
Synthesis of pyrrolopiperazinoquinazolines **13**.

**Scheme 10:**
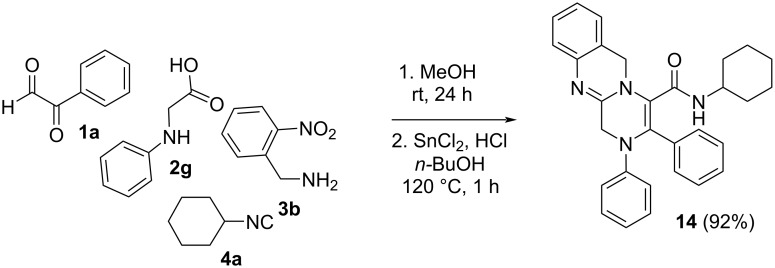
Synthesis of piperazinoquinazoline **14**.

Following the methodology previously described in our group [30], the reduction of the nitro group on indole and pyrrole derivatives **9f**,**g**,**l–o** (Scheme 9, Table 5) employing tin(II) chloride under acidic conditions in boiling *n*-butanol (120 °C) afforded the corresponding pyrrolopiperazinoquinazolines **13** with high yields. Interestingly, despite the acidic medium and high temperatures, the hemiaminal group was conserved, showing the high stability of these systems. On the other hand, the reduction of the *N-*phenylglycine derivative **10c**, used without purification after the Ugi/cyclization sequence, yielded the corresponding piperazinoquinazoline **14** with a high global yield (Scheme 10).

**Table 5:** Results obtained in the synthesis of pyrrolopiperazinoquinazolines **13**.

Entry	**9** (R^1^, R^2^, R^4^)	**13** (%)^a,b^

1	**9f** (pyrrole, H, *c*C_6_H_11_)	**13a** (70)
2	**9g** (pyrrole, 4-CF_3_, C(CH_3_)_3_)	**13b** (70)
3	**9l** (indole, H, *c*C_6_H_11_)	**13c** (72)
4	**9m** (indole, 4-F, *c*C_6_H_11_)	**13d** (68)
5	**9n** (indole, 4-CH_3_O, C(CH_3_)_3_)	**13e** (69)
6	**9o** (indole, 4-CF_3_, C(CH_3_)_3_)	**13f** (68)

^a^Yield after purification; ^b^the only diastereomer observed was the (3R*,4R*).

**Scheme 11:**
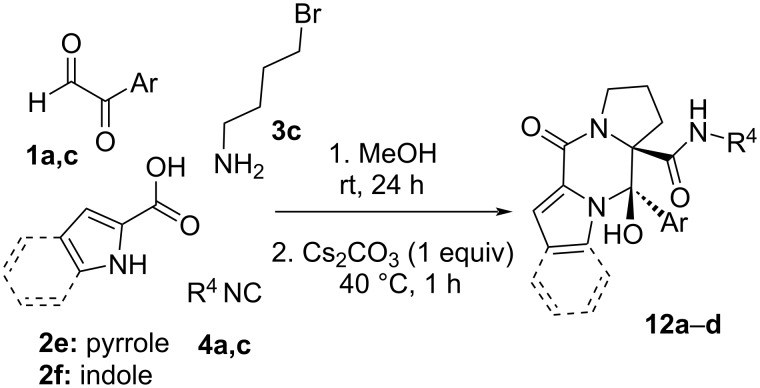
Synthesis of dipyrrolopiperazinones **12**.

**Figure 3:**
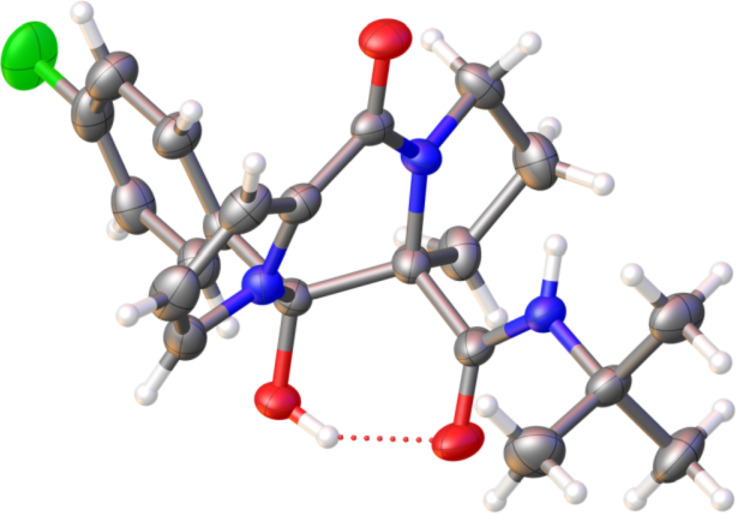
X-ray diffraction structure of dipyrrolopiperazinone **12c**. The thermal ellipsoid plot (Olex2) is at the 40 % probability level.

Moreover, the treatment of piperazinones **9c**–**e**,**k** derived from 3-bromopropanamine with caesium carbonate in boiling acetonitrile for 1 h afforded the corresponding dipyrrolopiperazinone **12** quantitatively. In view of the interest of these structures and the poor global yield obtained because of the purification in the first step, we tried the synthesis of these polycyclic systems in a one-pot sequence. In this way, after performing the Ugi reaction for 24 h, the reaction mixture, without purification, was treated with caesium carbonate (1 equiv) and heated to reflux for an hour. Gratefully, the dipyrrolopiperazinone derivatives **12** were obtained with high yields and complete diastereoselectivity in an efficient and simple way (Scheme 11, Table 6). The structure of these systems was further confirmed by single-crystal X-ray diffraction of compound **12c** (Figure 3).

**Table 6:** Results obtained in the synthesis of dipyrrolopiperazinones **12**.

Entry	**1** (Ar)	**2**	**4** (R^4^)	**12** (%)^a,b^

1	**1a** (C_6_H_5_)	**2e**	**4a** (*c*C_6_H_11_)	**12a** (93)
2	**1a** (C_6_H_5_)	**2e**	**4c** (C(CH_3_)_3_)	**12b** (74)
3	**1c** (4-FC_6_H_4_)	**2e**	**4c** (C(CH_3_)_3_)	**12c** (85)
4	**1a** (C_6_H_5_)	**2f**	**4c** (C(CH_3_)_3_)	**12d** (80)

^a^Yield after purification; ^b^the only diastereomer observed was the (3R*,4R*).

## Conclusion

In this work, we have demonstrated the straightforward access to a variety of complex nitrogen heterocycles by using unprotected deactivated amines tethered to carboxylic acids combined with arylglyoxals in the Ugi reaction. The reduced nucleophilic character of the amino group of the anthranilic acid, indole-2-carboxylic acid, pyrrole-2-carboxylic acid or *N*-phenylglycine allowed the use of these compounds in this multicomponent reaction without triggering competitive reactions. The presence of an additional functional group in the resulting Ugi adduct can be exploited in different post-condensation strategies to generate multiple fused nitrogen heterocycles in an easy manner.

## Supporting Information

File 1 General synthetic procedures and characterisation.

## Data Availability

All data that supports the findings of this study is available in the published article and/or the supporting information to this article.
