# Supplementary material for: Harnessing unprotected deactivated amines and arylglyoxals in the Ugi reaction for the synthesis of fused complex nitrogen heterocycles
Source: Beilstein J Org Chem. 2024 Jul 25;20:1758–66. doi: 10.3762/bjoc.20.154 (PMC11285059; doi:10.3762/bjoc.20.154)
Supplement: File 1 — General synthetic procedures and characterisation. [file Beilstein_J_Org_Chem-20-1758-s001.pdf]

BEILSTEIN JOURNAL OF ORGANIC CHEMISTRY

Supporting Information

for

Harnessing unprotected deactivated amines and arylglyoxals in the Ugi reaction for the synthesis of fused complex nitrogen heterocycles

Javier Gómez-Ayuso, Pablo Pertejo, Tomás Hermosilla, Israel Carreira-Barral, Roberto Quesada and María García-Valverde

Beilstein J. Org. Chem. **2024,** *20,* 1758–1766. doi:10.3762/bjoc.20.154

General synthetic procedures and characterisation

License and Terms: This is a supporting information file under the terms of the Creative Commons Attribution License (https://creativecommons.org/licenses/by/4.0). Please note that the reuse, redistribution and reproduction in particular requires that the author(s) and source are credited and that individual graphics may be subject to special legal provisions.
The license is subject to the *Beilstein Journal of Organic Chemistry* terms and conditions: (https://www.beilstein-journals.org/bjoc/terms)

Table of Contents

1. General information **S1**

2. Experimental information and characterization data **S1**

2.1. General procedure for the synthesis of benzodiazepinones **5** and **6** and pyrrolobenzodiazepinone **8** S1

2.2. General procedure for the synthesis of bis-benzodiazepines **7** S5

2.3. General procedure for the synthesis of piperazinones **9, 10** and **11** S6

2.4. General procedure for the synthesis of pirazinoquinazolines **13** and **14** S16

2.5. General procedure for the synthesis of dipyrrolopiperazinones **12** S20

3. X-ray crystallographic data **S23**

4. ^{1}H, ^{13}C, DEPT-135 and ^{19}F NMR spectra **S26**

4.1. Reaction mixtures in the synthesis of benzodiazepinone **6b** from (*S*)-α-methylbenzylamine**.** S26

4.2. Benzodiazepinones S27

4.3. Piperazinones S38

1. General information

Melting points are not corrected. Infrared spectra were registered in potassium bromide tablets. ^{1}H and ^{13}C NMR spectra were recorded in $CDCl_3$ and DMSO-d_6 at 300 and 75 MHz on a Varian Mercury 300. Chemical shifts are reported in parts per million with respect to residual solvent protons, and coupling constants are reported in hertz. Low resolution mass spectra were recorded in the positive ion mode by electronic impact at 70 eV. High-resolution mass spectra were recorded in the positive ion mode by electronic impact at 70 eV or positive electrospray ionization mode, ESI(+). X-ray diffraction studies were performed on a Bruker D8 VENTURE diffractometer.

2. Experimental information and characterization data

2.1. General procedure for the synthesis of benzodiazepinones 5 and 6 and pyrrolobenzodiazepinone 8

Arylglyoxal hydrate **1** (2 mmol, 1 equiv) was dissolved in methanol (7 mL), after which the corresponding amine **3** (2 mmol, 1 equiv) was added [2-nitrobenzylamine **3b** and 3-bromopropylamine **3c** were obtained from the treatment of the commercial salts (2.1 mmol, 2.1 equiv) with sodium hydroxide (2.0 mmol, 2.0 equiv) in methanol]. The mixture was stirred for 20 minutes at room temperature and then the corresponding acid **2** (2 mmol, 1 equiv.) and the isocyanide **4** (2 mmol, 1 equiv) were added. After 24 hours of reaction, the solvent was removed under reduced pressure and the residue dissolved in dichloromethane (30 mL). This solution was washed with a 1 M hydrochloric acid aqueous solution (2 × 50 mL) and a saturated sodium carbonate aqueous solution (1 × 50 mL). The organic phase was dried over anhydrous sodium sulfate, filtered and concentrated to dryness. The products were purified by flash column chromatography (SiO_2, hexane/ethyl acetate).

Compounds **5a**,[1] **5d**,[2] **6a-6f** (except *l*-**6c**),[3] and **8**[4] have been previously described.

---

[1] Sañudo, M.; García-Valverde, M.; Marcaccini, S.; Delgado, J. J.; Rojo, J.; Torroba, T. *J. Org. Chem.* **2009**, *74*, 2189-2192.
[2] Pertejo, P.; García-Valverde, M.; Peña, P.; Cordero, N. A.; Torroba, T.; González-Ortega, A. *Org. Biomol. Chem.* **2014**, *12*, 4905-4916.
[3] Pertejo, P.; Corres, N.; Torroba, T.; García-Valverde, M. *Org. Lett.* **2015**, *17*, 612-615.
[4] Pertejo, P.; Carreira-Barral, I.; Peña-Calleja, P.; Quesada, M.; García-Valverde, M. *J. Org. Chem.* **2020**, *85*, 2291-2302.

_N_-Cyclohexyl-4-(2-nitrobenzyl)-5-oxo-2-phenyl-4,5-dihydro-_3H_-benzo[_e_][1,4]diazepine-3-carboxamide. (5b)

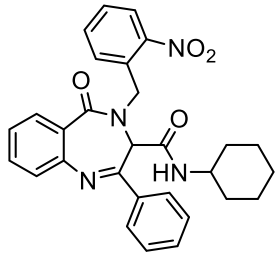

White solid (80%, 794 mg). M.p. 122-124 ºC. Rf = 0.26 (hexane/ethyl acetate 2:1).

^{1}H NMR (300 MHz, $CDCl_3$) δ: 8.15-6.60 (m, 13H), 5.47 (s, 1H), 5.45 (d, J = 8.5 Hz, 1H), 5.37 (d, J = 15.8 Hz, 1H), 5.08 (d, J = 15.8 Hz, 1H), 3.39–3.22 (m, 1H), 1.80–0.48 (m, 10H).

^{13}C {^{1}H} NMR (75 MHz, $CDCl_3$) δ: 168.0 (Cq), 164.7 (Cq), 164.2 (Cq), 148.7 (Cq), 146.0 (Cq), 137.4 (Cq), 133.6 (CH), 132.2 (CH), 131.4 (CH), 131.24 (CH), 131.2 (Cq), 130.7 (CH), 128.9 (CH), 128.8 (CH), 127.5 (CH), 127.2 (CH), 126.6 (CH), 125.9 (Cq), 124.7 (CH), 60.0 (CH), 49.2 (CH_2), 48.5 (CH), 32.4 (CH_2), 25.2 (CH_2), 24.7 (CH_2), 24.6 (CH_2).

HRMS (EI) calculated for ($C_{29}H_{28}N_4O_4$) [M^+]: 496.2111; found: 496.2116.

4-(3-Bromopropyl)-_N_-cyclohexyl-5-oxo-2-p-tolyl-4,5-dihydro-3_H_-benzo[_e_][1,4]diazepine-3-carboxamide. (5c)

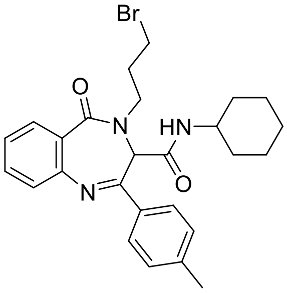

Sticky solid (58%, 574 mg). Rf = 0.23 (hexane/ethyl acetate 2:1).

^{1}H NMR (300 MHz, $CDCl_3$) δ: 8.03 – 7.10 (m, 8H), 5.31 (s, 1H), 5.27 (d, J = 8.4 Hz, 1H), 3.85 (m, 1H), 3.70 (m, 1H), 3.58 – 3.44 (m, 1H), 3.41 – 3.23 (m, 2H), 2.42 (s, 3H), 2.28 – 1.85 (m, 1H), 1.70 – 0.46 (m, 10H).

^{13}C {^{1}H} NMR (75 MHz, $CDCl_3$) δ: 167.5 (Cq), 164.4 (Cq), 163.9 (Cq), 145.8 (Cq), 142.4 (Cq), 134.6 (Cq), 131.9 (CH), 130.6 (CH), 129.9 (CH), 127.7 (CH), 127.1 (CH), 126.5 (CH), 126.4 (Cq), 61.0 (CH), 49.4 (CH_2), 48.6 (CH), 32.4 (CH_2), 32.4 (CH_2), 31.4 (CH_2), 31.1 (CH_2), 25.2 (CH_2), 24.6 (CH_2), 21.5 (CH_2).

HRMS (EI) calculated for ($C_{26}H_{30}BrN_3O_2$) [M^+]: 495.1521; found: 495.1523.

4-Benzyl-_N_-cyclohexyl-8-nitro-5-oxo-2-phenyl-4,5-dihydro-3_H_-benzo[_e_][1,4]diazepine-3-carboxamide. (5e)

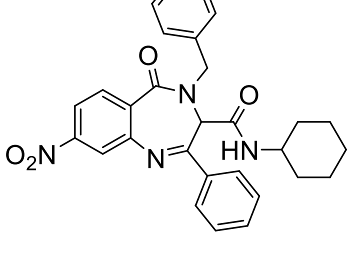

Yellow solid (68%, 675 mg). M.p.: 133-135 ºC. Rf = 0.24 (hexane/ethyl acetate 2:1).

IR (KBr, cm^{-1}): 3334 (NH); 2932; 2856; 1634 (C=O); 1525; 1350.

^{1}**H NMR** (300MHz, $CDCl_3$) δ: 8.24 (d, *J* = 2.2 Hz, 1H), 8.18 (d, *J* = 8.7 Hz, 1H), 8.07 (dd, *J* = 8.7, 2.2 Hz, 1H), 7.83 (d, *J* = 7.4 Hz, 2H), 7.57-7.31 (m, 8H), 5.37 (s, 1H), 5.30 (d, *J* = 14.3 Hz,1H), 4.98 (d, *J* = 8.2 Hz, 1H), 4.46 (d, *J* = 14.3 Hz, 1H), 3.23-3.11 (m, 1H), 1.46-0.93 (m, 8H), 0.56-0.42 (m, 2H).

^{13}**C {^{1}H} NMR** (75MHz, $CDCl_3$) δ: 166.7 (Cq), 166.0 (Cq), 163.9 (Cq), 149.9 (Cq), 147.1 (Cq), 137.0 (Cq), 136.1 (Cq), 132.7 (CH),132.3 (CH), 131.3 (Cq), 129.8 (CH), 129.3 (CH), 129.2 (CH), 128.0 (CH), 122.8 (CH), 120.2 (CH), 58.9 (CH), 53.2 (CH_2), 48.5 (CH), 32.5 (CH_2), 32.4 (CH_2), 25.3 (CH_2), 24.7 (CH_2), 24.6 (CH_2).

MS (EI) m/z (relative intensity) 496 (M^+, 5); 372 (56); 371 (65); 371 (75); 281 (19); 280 (100); 91 (69).

HRMS (EI) calculated for $C_{29}H_{28}N_4O_4$ [M^+]: 496.2111; found: 496.2114.

4-Benzyl-*N*-cyclohexyl-7-nitro-5-oxo-2-phenyl-4,5-dihydro-3*H*-benzo[*e*][1,4]diazepine-3-carboxamide. (5f)

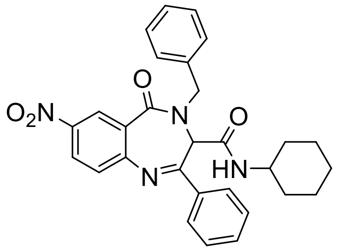

Yellow solid (63%, 625 mg). M.p.169-171 ºC. Rf = 0.26 (hexane/ethyl acetate 2:1).

IR (KBr, cm^{-1}): 3334 (NH); 2929; 1650 (C=O);1632; 1519; 1343.

^{1}**H NMR** (300MHz, $CDCl_3$) δ: 8.89 (d, *J* = 2.6 Hz, 1H), 8.31 (dd, *J* = 8.8, 2.6 Hz, 1H), 7.90 (d, *J* = 7.1 Hz, 2H), 7.61-7.33 (m, 9H), 5.45 (d, *J* = 14.3 Hz, 1H), 5.39 (s, 1H), 5.01 (d, *J* = 8.3 Hz, 1H), 4.27 (d, *J* = 14.3 Hz, 1H), 3.22-3.08 (m, 1H), 1.45-0.95 (m, 8H), 0.57-0.36 (m, 2H).

^{13}**C {^{1}H} NMR** (75MHz, $CDCl_3$) δ: 167.0 (Cq), 165.7 (Cq), 164.0 (Cq), 151.1 (Cq), 145.2 (Cq), 137.0 (Cq), 136.2 (Cq),132.5 (CH), 129.9 (CH), 129.3 (CH), 128.6 (CH), 128.2 (CH), 127.3 (CH), 126.8 (Cq), 126.6 (CH), 59.1 (CH), 53.3 (CH_2), 48.4 (CH) 32.4 (CH_2), 32.1 (CH_2), 25.2 (CH_2), 24.7 (CH_2), 24.5 (CH_2).

MS (EI) m/z (relative intensity) 496 (M^+, 3); 372 (28); 371 (100); 280 (62); 178 (17); 91(60); 91 (54).

HRMS (EI) calculated for $C_{29}H_{28}N_4O_4$ [M^+]: 496.2111; found: 496.2100.

4-Benzyl-*N*-cyclohexyl-7-iodo-5-oxo-2-phenyl-4,5-dihydro-3*H*-benzo[*e*][1,4]diazepine-3-carboxamide. (5g)

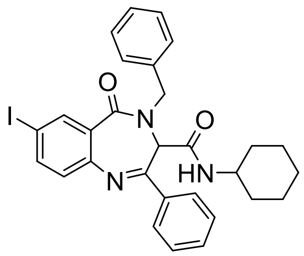

White solid (67%, 773 mg). M.p 186-187 ºC. Rf = 0.33 (hexane/ethyl acetate 2:1).

IR (KBr, cm^{-1}): 3324 (NH); 2931; 2854;1674; 1625 (C=O);1520; 1453; 1259.

^{1}H NMR (300 MHz, $CDCl_3$) δ: 8.30 (d, *J* =2.0 Hz, 1H), 7.83-7.75 (m, 3H), 7.52-7.29 (m, 8H), 7.10 (d, *J* = 8.5 Hz, 1H), 5.35 (d, *J* = 14.2 Hz, 1H), 5.30 (s, 1H), 5.03 (d, *J* = 8.4 Hz, 1H), 4.30 (d, *J* = 14.2 Hz, 1H), 3.29-3.15 (m, 1H), 1.48-1.03 (m, 8H), 0.60-0.46 (m, 10H).

^{13}C {^{1}H} NMR (75MHz, $CDCl_3$) δ: 166.2 (Cq), 165.3 (Cq), 164.4 (Cq), 146.1 (Cq), 141.1 (CH), 139.4 (CH), 137.8 (Cq), 136.5 (Cq), 131.7 (CH), 129.7 (CH), 129.2 (CH), 129.1 (CH), 129.0 (CH), 127.8 (CH), 90.7 (Cq), 59.3 (CH), 53.1 (CH_2), 48.3 (CH), 32.7 (CH_2), 32.2 (CH_2), 25.4 (CH_2), 24.8 (CH_2), 24.7 (CH_2).

MS (EI) m/z (relative intensity) 577 (M^+, 16); 453 (60); 452 (86); 452 (78); 361 (100); 272 (40); 271 (52); 178 (29); 91(85); 91(69).

HRMS (EI) calculated for $C_{29}H_{28}IN_3O_2$ [M^+]: 577.1226; found: 577.1221.

***N*-Cyclohexyl-7-iodo-4-(2-nitrobenzyl)-5-oxo-2-phenyl-4,5-dihydro-3*H*-benzo[*e*][1,4]diazepine-3-carboxamide. (5h)**

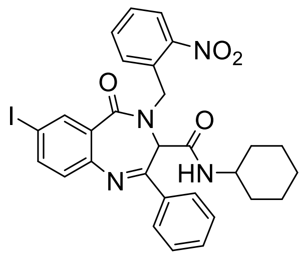

Yellow solid (61%, 759 mg). M.p. 95-96 ºC. Rf = 0.34 (hexane/ethyl acetate 2:1).

^{1}H NMR (300 MHz, $CDCl_3$) δ: 8.41 – 7.00 (m, 12H), 5.50 (s, 1H), 5.40 (d, *J* = 8.4 Hz, 1H), 5.26 (d, *J* = 15.5 Hz, 1H), 5.14 (d, *J* = 15.5 Hz, 1H), 3.52 – 3.19 (m, 1H), 1.72 – 0.50 (m, 10H).

^{13}C {^{1}H} NMR (75 MHz, $CDCl_3$) δ: 166.6 (Cq), 165.2 (Cq), 164.0 (Cq), 148.8 (Cq), 145.6 (Cq), 141.0 (CH), 139.1 (CH), 137.1 (Cq), 133.7 (CH), 131.6 (CH), 131.5 (CH), 130.8 (Cq), 129.2 (CH), 128.9 (CH), 128.8 (CH), 127.6 (Cq), 127.6 (CH), 124.8 (CH), 90.9 (Cq), 59.7 (CH), 49.0 (CH_2), 48.5 (CH), 32.6 (CH_2), 32.3 (CH_2), 25.2 (CH_2), 24.7 (CH_2).

HRMS (EI) calculated for $C_{29}H_{27}IN_4O_4$ [M^+]: 622.1077; found: 622.1072.

(3*S*)-*N*-Cyclohexyl-2-(4-fluorophenyl)-4-((S)-methylbenzyl)-5-oxo-4,5-dihydro-3*H*-benzo[*e*][1,4]diazepine-3-carboxamide. (*l*-6c)

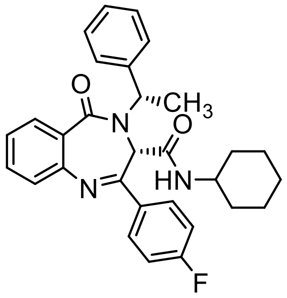

Yellow solid (58%, 564 mg). M. p. 136-138 °C. $[\alpha]_D^{20}$ = +54.2 (c = 1.06, acetone). Rf = 0.24 (hexane/ethyl acetate 2:1).

IR (KBr, cm^{-1}). 3413; 2930; 2856; 1693; 1638 (C=O), 1514, 1340.

^{1}H NMR (300 MHz, $CDCl_3$) δ: 8.01-7.83 (m, 3H), 7.53-7.2 (m, 10H), 6.32 (q, *J* = 7.0 Hz, 1H), 5.14 (s, 1H), 4.97 (d, *J* = 8.3 Hz, 1H), 3.19-2.92 (m, 1 H), 1.59-0.18 (m, 10H), 1.17 (d, *J* = 7.0 Hz, 3H).

^{13}C {^{1}H} NMR (75 MHz, $CDCl_3$) δ: 167.3 (Cq), 166.3 (Cq), 165.0 (Cq), 164.6 (d, ^1J = 252.4 Hz, C-F), 145.5 (Cq), 139.5 (Cq), 134.8 (Cq), 134.8 (Cq), 132.4 (CH), 130.7 (CH), 130.2 (CH), 130.1 (CH), 129.8 (CH), 129.2 (CH), 127.9 (CH), 127.4 (CH), 126.4 (CH), 126.1 (Cq), 116.2 (d, ^2J = 21.9 Hz, C-F), 55.5 (CH), 53.0 (CH), 48.0 (CH), 32.5 (CH_2), 32.1 (CH_2), 25.4 (CH_2), 24.8 (CH_2), 24.6 (CH_2), 17.6 (CH_3).

MS (EI) m/z (relative intensity) 483 (M+, 14); 358 (46); 254 (61); 253 (100);105 (90); 83 (36).

HRMS (EI) calculated for $C_{30}H_{30}FN_3O_2$ [M$^+$]: 483.2322; found: 483.2328.

2.2. General procedure for the synthesis of bis-benzodiazepines 7

Benzodiazepine **5b,h** (0.5 mmol, 1 equiv), tin(II) chloride (5 mmol, 10 equiv) and HCl (1.5 mmol, 3 equiv) were added to 30 mL of *n*-butanol. The mixture is then heated to 120 °C and stirred for 1 hour. After that, the solvent is removed and the crude is redissolved in dichloromethane (50 mL) and washed with a saturated solution of sodium carbonate (3 x 40 mL). The organic phase was dried over anhydrous sodium sulfate, filtered and concentrated to dryness. The product was purified by flash column chromatography (SiO_2, hexane/ethyl acetate).

(6*R,15*S**)-*N*-Cyclohexyl-6-phenyl-5,7,12,14-tetrahydro-6*H*-6,13-methanodibenzo[*d,i*][1,3,7]triazecine-15-carboxamide. (7a)**

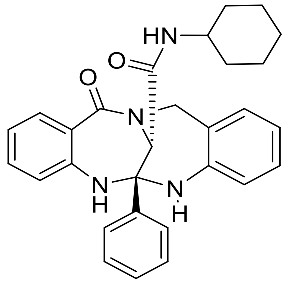

Sticky solid (70%, 163 mg). Rf = 0.28 (hexane/ethyl acetate 2:1).

^{1}H NMR (300 MHz, $CDCl_3$) δ: 7.90 – 6.71 (m, 13H), 4.92 (d, *J* = 8.2 Hz, 1H), 4.60 (d, *J* = 11.0 Hz, 1H), 3.91 (d, *J* = 11.0 Hz, 1H), 3.35 (s, 1H), 3.36 – 3.23 (m, 1H), 1.78 – 0.11 (m, 10H).

^{13}C {^{1}H} NMR (75 MHz, $CDCl_3$) δ: 168.6 (Cq), 162.1 (Cq), 146.0 (Cq), 143.2 (Cq), 139.0 (Cq), 134.1 (CH), 132.7 (Cq), 130.3 (CH), 129.6 (CH), 129.4 (CH), 128.5 (CH), 128.3 (CH), 127.9 (CH), 127.8 (CH), 126.5 (CH), 119.0 (CH), 116.2 (Cq), 114.8 (CH), 77.2 (Cq), 64.9 (CH), 48.0 (CH), 47.4 (CH_2), 33.0 (CH), 31.9 (CH_2), 25.1 (CH_2), 24.4 (CH_2), 24.3 (CH_2).

HRMS (EI) calculated for ($C_{29}H_{30}N_4O_2$) [M^+]: 466.2369; found: 466.2374.

(6*R,15*S**)-*N*-Cyclohexyl-6-phenyl-2-iodo-5,7,12,14-tetrahydro-*6H*-6,13-methanodibenzo[*d,i*][1,3,7]triazecine-15-carboxamide. (7b)**

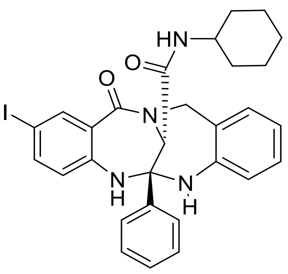

White solid (74%, 219 mg). M. p. 222-223 ºC. Rf = 0.36 (hexane/ethyl acetate 2:1).

^{1}H NMR (300 MHz, $CDCl_3$) δ: 8.06 – 7.08 (m, 12H), 6.77 (d, *J* = 8.5 Hz, 1H), 4.91 (d, *J* = 7.9 Hz, 1H), 4.55 (d, *J* = 11.0 Hz, 1H), 3.91 (d, *J* = 11.0 Hz, 1H), 3.33 (s, 1H), 3.36-3.20 (m, 1H) 1.71 – 0.14 (m, 12H).

^{13}C {^{1}H} NMR (75MHz, $CDCl_3$) δ: 168.6 (Cq), 160.7 (Cq), 145.5 (Cq), 142.8 (Cq), 142.3 (CH), 138.7 (Cq), 137.8 (CH), 132.6 (Cq), 130.2 (CH), 129.7 (CH), 128.6 (CH), 128.4 (CH), 128.2 (CH), 127.9 (CH), 126.4 (CH), 118.2 (Cq), 117.0 (CH), 80.0 (Cq), 77.3 (Cq), 64.9 (CH_2), 48.0 (CH_2), 47.4(CH_2), 33.0 (CH) 32.1 (CH_2), 31.9 (CH_2), 25.1 (CH_2), 24.4 (CH_2), 24.3 (CH_2).

HRMS (EI) calculated for ($C_{29}H_{29}IN_4O_2$) [M^+]: 592.1335; found: 592.1326.

2.3. General procedure for the synthesis of piperazinones 9, 10 and 11

Arylglyoxal hydrate **1** (2 mmol, 1 equiv) was dissolved in methanol (7 mL), after which the corresponding amine **3** (2 mmol, 1 equiv), was added. The mixture was stirred for 20 minutes at room temperature and then the corresponding acid **2** (2 mmol, 1 equiv) and the isocyanide **4** (2 mmol, 1 equiv) were added. The corresponding mixture was stirred at room temperature for 24 hours and the obtained precipitate was isolated by vacuum filtration, washed in cold methanol and dried in vacuo, affording the corresponding piperazinone **9** and **10**. In the case of products **9c–e** and **9k**, a precipitate was not formed. So, after 24 hours of reaction, a 1 M hydrochloric acid aqueous solution (15 mL) was added to the solution. The methanol was removed under reduced pressure and the residue treated with dichloromethane (30 mL). The resulting mixture was washed with a 1 M

hydrochloric acid aqueous solution (2 × 50 mL) and a saturated sodium carbonate aqueous solution (1 × 50 mL). The organic phase was dried over anhydrous sodium sulfate, filtered and concentrated to dryness. The products **9c-e,k** were purified by flash column chromatography (SiO_2, hexane/ethyl acetate). Diastereomers of **11** were also separated by flash column chromatography (SiO_2, hexane/ethyl acetate).

In the cases of *tert*-butyl amine derivatives a mixture between the Ugi adduct and the corresponding piperazinone **9b** and **9j** was formed. 0.16 mL of HCl 37% (2 mmol, 1 equiv) were added to the mixture and it was then stirred for another 16 hours. The solvent was then removed under reduced pressure and the residue dissolved in dichloromethane (30 mL). This solution was washed with a saturated sodium carbonate aqueous solution (2 × 50 mL). The organic phase was dried over anhydrous sodium sulfate, filtered and concentrated to dryness. The products were purified by crystallization (in 2-propanol) or flash column chromatography (SiO_2, hexane/ethyl acetate from 10:1 to 2:1).

(3*R,4*R**)-2-Benzyl-*N*-(*tert*-butyl)-4-hydroxy-1-oxo-4-phenyl-1,2,3,4-tetrahydropyrrolo[1,2-a]pyrazine-3-carboxamide. (9a)**

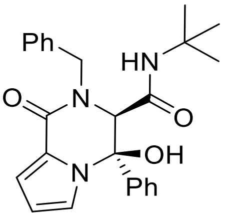

Grey Solid (61%, 509 mg). M.p. = 181-182 ºC. Rf = 0.15 (hexane/ethyl acetate 4:1)

^{1}H NMR (300 MHz, $CDCl_3$) δ 8.37 (s, 1H, OH), 7.30 – 7.01 (m, 10H, H_{Ar}), 6.73 (d, *J* = 8.3 Hz, 2H, H_{Ar}), 6.39 – 6.24 (m, 1H, H_{Ar}), 5.72 (s, 1H, NH), 4.55 (d, *J* = 14.3 Hz, 1H), 4.10 (s, 1H), 3.96 (d, *J* = 14.3 Hz, 1H), 0.96 (s, 9H).

^{13}C {^{1}H} NMR (75 MHz, $CDCl_3$) δ 170.3 (CO), 158.8 (CO), 141.3 (Cq_{Ar}), 135.8 (Cq_{Ar}), 129.2 (CH_{Ar}), 129.1 (CH_{Ar}), 129.0 (CH_{Ar}), 128.6 (CH_{Ar}), 128.2 (CH_{Ar}), 125.3 (CH_{Ar}), 123.3 (Cq_{Ar}), 121.9 (CH_{Ar}), 115.4 (CH_{Ar}), 110.7 (CH_{Ar}), 88.4 (Cq), 65.9 (CH), 52.2 (Cq), 50.7 (CH_2), 27.8 (CH_3).

HRMS (ESI) calculated for ($C_{25}H_{28}N_3O_3$) ($[M+H]^+$): 418.2125; found: 418.2137.

(3*R,4*R**)-2-(*tert*-Butyl)-*N*-cyclohexyl-4-hydroxy-1-oxo-4-phenyl-1,2,3,4-tetrahydropyrrolo[1,2-*a*]pyrazine-3-carboxamide. (9b)**

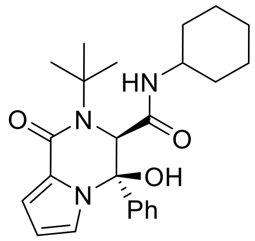

White Oil (52%, 426 mg). Rf = 0.16 (hexane/ethyl acetate 4:1).

^{1}H NMR (300 MHz, $CDCl_3$) δ 8.38 (s, 1H, OH), 7.31 – 7.19 (m, 3H, H_{Ar}), 7.12 – 7.04 (m, 1H, H_{Ar}), 7.00 – 6.93 (m, 1H, H_{Ar}), 6.86 – 6.74 (m, 2H, H_{Ar}), 6.40 (d, *J* = 8.1 Hz, 1H, NH), 6.27 (t, *J* = 3.2 Hz, 1H, H_{Ar}), 4.47 (s, 1H), 3.76 – 3.58 (m, 1H), 1.83 – 0.77 (m, 10H), 1.09 (s, 9H).

^{13}C {^{1}H} NMR (75 MHz, $CDCl_3$) δ 171.4 (CO), 159.8 (CO), 141.4 (Cq_{Ar}), 129.1 (CH_{Ar}), 128.5 (CH_{Ar}), 125.7 (CH_{Ar}), 124.9 (Cq_{Ar}), 120.7 (CH_{Ar}), 114.9 (CH_{Ar}), 110.8 (CH_{Ar}), 88.7 (Cq), 63.9 (CH), 57.7 (Cq), 48.6 (CH), 32.1 (CH_2), 32.0 (CH_2), 28.5 (CH_3), 25.1 (CH_2), 24.3 (CH_2), 24.0 (CH_2).

HRMS (ESI) calculated for ($C_{24}H_{32}N_3O_3$) ($[M+H]^+$): 410.2438; found: 410.2435.

(3*R,4*R**)-2-(3-Bromopropyl)-*N*-cyclohexyl-4-hydroxy-1-oxo-4-phenyl-1,2,3,4-tetrahydropyrrolo[1,2-*a*]pyrazine-3-carboxamide. (9c)**

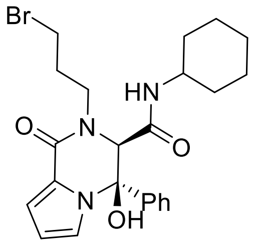

Orange oil (16%, 152 mg). Rf = 0.44 (hexane/ethyl acetate 2:1).

^{1}H NMR (300 MHz, $CDCl_3$) δ 7.61 – 7.21 (m, 5H), 7.18 (dd, *J* = 2.7, 1.6 Hz, 1H, H_{Ar}), 7.05 (dd, *J* = 3.8, 1.6 Hz, 1H, H_{Ar}), 6.86 – 6.78 (m, 1H, H_{Ar}), 6.40 – 6.31 (m, 1H, H_{Ar}), 4.29 – 4.12 (m, 2H), 3.79 – 3.61 (m, 2H), 2.90 – 2.67 (m, 2H), 2.53 – 2.40 (m, 1H), 1.86 – 0.79 (m, 10H).

^{13}C {^{1}H} NMR (75 MHz, $CDCl_3$) δ 169.8 (CO), 159.3 (CO), 141.7 (Cq_{Ar}), 133.3 (Cq_{Ar}), 129.7 (CH_{Ar}), 128.8 (CH_{Ar}), 125.6 (CH_{Ar}), 122.4 (CH_{Ar}), 115.7 (CH_{Ar}), 111.2 (CH_{Ar}), 88.2 (Cq), 67.0 (CH), 49.1 (CH), 45.1 (CH_2), 32.2 (CH_2), 32.2 (CH_2), 30.9 (CH_2), 29.9 (CH_2), 25.3 (CH_2), 24.6 (CH_2), 24.4 (CH_2).

HRMS (ESI) calculated for ($C_{23}H_{29}BrN_3O_3$) ($[M+H]^+$): 474.1387; found: 474.1390.

(3*R,4*R**)-2-(3-Bromopropyl)-*N*-(*tert*-butyl)-4-hydroxy-1-oxo-4-phenyl-1,2,3,4-tetrahydropyrrolo[1,2-*a*]pyrazine-3-carboxamide. (9d)**

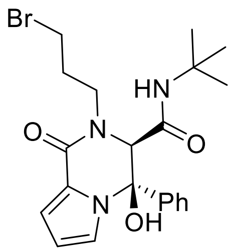

Brown oil (24%, 214 mg). Rf = 0.44 (hexane/ethyl acetate 2:1).

^{1}H NMR (300 MHz, $CDCl_3$) δ 8.31 (s, 1H, OH), 7.36 – 7.27 (m, 3H, H_{Ar}), 7.21 – 7.15 (m, 1H, H_{Ar}), 7.11 – 7.06 (m, 1H, H_{Ar}), 6.87 – 6.78 (m, 2H, H_{Ar}), 6.39 – 6.32 (m, 1H, H_{Ar}), 5.92 (s, 1H, NH), 4.23 – 4.04 (m, 2H), 2.86 (ddd, *J* = 10.4, 6.7, 5.5 Hz, 1H), 2.75 (dt, *J* = 13.7, 6.4 Hz, 1H), 2.46 (ddd, *J* = 10.4, 6.9, 6.0 Hz, 1H), 1.44 (tt, *J* = 6.5, 3.0 Hz, 2H), 1.22 (s, 9H).

^{13}C {^{1}H} NMR (75 MHz, $CDCl_3$) δ 170.5 (CO), 159.2 (CO), 141.7 (Cq_{Ar}), 129.6 (CH_{Ar}), 128.8 (CH_{Ar}), 125.6 (CH_{Ar}), 123.3 (Cq_{Ar}), 122.3 (CH_{Ar}), 115.5 (CH_{Ar}), 111.1 (CH_{Ar}), 88.3 (Cq), 67.2 (CH), 52.9 (Cq), 45.1 (CH_2), 31.0 (CH_2), 30.0 (CH_2), 28.3 (CH_3).

HRMS (ESI) calculated for ($C_{21}H_{27}BrN_3O_3$) ($[M+H]^+$): 448.1230; found: 448.1228.

(3*R,4*R**)-2-(3-Bromopropyl)-*N*-(*tert*-butyl)-4-(4-fluorophenyl)-4-hydroxy-1-oxo-1,2,3,4-tetrahydropyrrolo[1,2-*a*]pyrazine-3-carboxamide. (9e)**

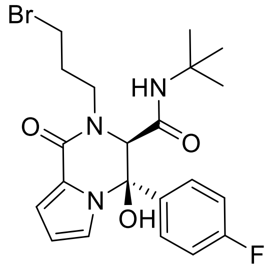

Brown oil (22%, 205 mg). Rf = 0.45 (hexane/ethyl acetate 2:1).

^{1}H NMR (300 MHz, $CDCl_3$) δ 8.35 (s, 1H, OH), 7.18 – 7.15 (m, 1H, H_{Ar}), 7.08 – 7.05 (m, 1H, H_{Ar}), 7.01 – 6.94 (m, 2H, H_{Ar}), 6.84 – 6.77 (m, 2H, H_{Ar}), 6.39 – 6.31 (m, 1H, H_{Ar}), 4.21 – 4.08 (m, 1H), 4.03 (s, 1H), 2.95 (dt, *J* = 10.3, 6.5 Hz, 1H), 2.77 (dt, *J* = 13.4, 6.4 Hz, 1H), 2.57 (dt, *J* = 10.3, 6.5 Hz, 1H), 1.48 (p, *J* = 8.1, 7.4 Hz, 2H), 1.21 (s, 9H).

^{13}C {^{1}H} NMR (75 MHz, $CDCl_3$) δ 170.4 (CO), 163.3 (d, ^1J = 249.4 Hz, Cq_{Ar}), 159.0 (CO), 137.7 (d, ^4J = 3.1 Hz, Cq_{Ar}), 127.7 (d, ^3J = 8.4 Hz, CH_{Ar}), 123.2 (Cq_{Ar}), 122.2 (CH_{Ar}), 115.80 (d, ^2J = 16.1 Hz, CH_{Ar}), 115.6 (CH_{Ar}), 111.3 (CH_{Ar}), 87.9 (Cq), 67.2 (CH), 53.0 (Cq), 45.2 (CH_2), 31.1 (CH_2), 29.8 (CH_2), 28.3 (CH_3).

^{19}F NMR (282 MHz, $CDCl_3$) δ -111.72 (tt, *J* = 8.2, 5.2 Hz).

HRMS (ESI) calculated for ($C_{21}H_{26}BrFN_3O_3$) ($[M+H]^+$): 466.11361; found: 466.11362.

(3*R,4*R**)-*N*-Cyclohexyl-4-hydroxy-2-(2-nitrobenzyl)-1-oxo-4-phenyl-1,2,3,4-tetrahydropyrrolo[1,2-*a*]pyrazine-3-carboxamide. (9f)**

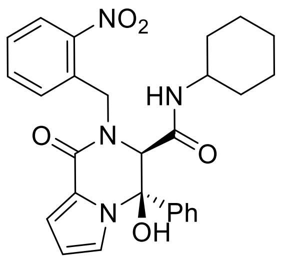

Brown solid (77%, 711 mg) (crystallized in iPrOH). M.p. = 176 – 177 ºC.

^{1}H NMR (300 MHz, $CDCl_3$) δ 7.89 – 7.80 (m, 1H, H_{Ar}), 7.34 – 7.02 (m, 7H, H_{Ar}), 6.74 – 6.59 (m, 3H, H_{Ar}), 6.41 – 6.34 (m, 1H, H_{Ar}), 6.22 (d, J = 8.1 Hz, 1H, NH), 5.24 (d, J = 15.4 Hz, 1H), 4.42 (s, 1H), 4.42 (d, J = 15.4 Hz, 1H), 3.78 – 3.63 (m, 1H), 1.83 – 1.43 (m, 5H), 1.41 – 0.81 (m, 5H).

^{13}C {^{1}H} NMR (75 MHz, $CDCl_3$) δ 169.7 (CO), 159.4 (CO), 148.7 (Cq_{Ar}), 141.8 (Cq_{Ar}), 133.8 (CH_{Ar}), 131.1 (CH_{Ar}), 130.7 (Cq_{Ar}), 129.2 (CH_{Ar}), 128.7 (CH_{Ar}), 128.6 (CH_{Ar}), 125.6 (CH_{Ar}), 124.7 (CH_{Ar}), 123.0 (Cq_{Ar}), 122.8 (CH_{Ar}), 116.2 (CH_{Ar}), 111.2 (CH_{Ar}), 88.2 (Cq), 65.9 (CH), 49.1 (CH), 45.2 (CH_2), 32.2 (CH_2), 32.2 (CH_2), 25.3 (CH_2), 24.6 (CH_2), 24.4 (CH_2).

HRMS (ESI) calculated for ($C_{27}H_{29}N_4O_5$) ($[M+H]^+$): 489.2132; found: 489.2135.

(3*R,4*R**)-*N*-(*tert*-Butyl)-4-hydroxy-2-(2-nitrobenzyl)-1-oxo-4-(4-(trifluoromethyl)phenyl)-1,2,3,4-tetrahydropyrrolo[1,2-*a*]pyrazine-3-carboxamide. (9g)**

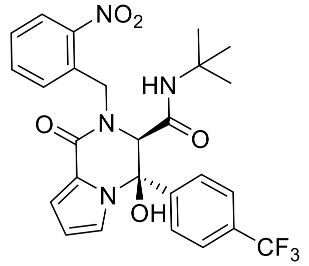

White solid (69%, 731 mg) (crystallized in iPrOH). M.p. = 203 – 204 ºC.

^{1}H NMR (300 MHz, $CDCl_3$) δ 8.48 (s, 1H, OH), 7.78 (dd, J = 8.2, 1.1 Hz, 1H, H_{Ar}), 7.37 – 7.24 (m, 3H, H_{Ar}), 7.22 – 7.13 (m, 2H, H_{Ar}), 7.08 (td, J = 7.7, 1.2 Hz, 1H, H_{Ar}), 6.88 – 6.81 (m, 1H, H_{Ar}), 6.68 (d, J = 8.1 Hz, 2H, H_{Ar}), 6.44 – 6.35 (m, 1H, H_{Ar}), 5.98 (s, 1H, NH), 5.32 (d, J = 14.8 Hz, 1H), 4.42 (s, 1H), 4.33 (d, J = 14.8 Hz, 1H), 1.19 (s, 9H).

^{13}C {^{1}H} NMR (75 MHz, $CDCl_3$) δ 170.0 (CO), 159.1 (CO), 148.8 (Cq_{Ar}), 145.5 (Cq_{Ar}), 133.7 (CH_{Ar}), 131.9 (CH_{Ar}), 130.3 (Cq_{Ar}), 129.1 (CH_{Ar}), 126.0 (CH_{Ar}), 125.5 (q, J = 3.6 Hz, CH_{Ar}), 124.5 (CH_{Ar}), 122.7 (Cq_{Ar}), 122.6 (CH_{Ar}), 116.5 (CH_{Ar}), 111.6 (CH_{Ar}), 88.0 (Cq), 65.1 (CH), 53.0 (Cq), 44.1 (CH_2), 28.3 (CH_3).

HRMS (ESI) calculated for ($C_{26}H_{26}F_3N_4O_5$) ($[M+H]^+$): 531.1850; found: 531.1856.

(3*R,4*R**)-2-Benzyl-*N*-cyclohexyl-4-hydroxy-1-oxo-4-phenyl-1,2,3,4-tetrahydropyrazino[1,2-*a*]indole-3-carboxamide. (9h)**

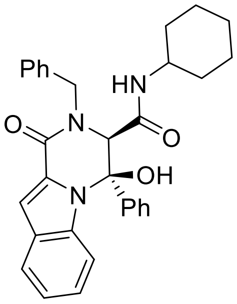

White Solid (49%, 483 mg) (crystallized in iPrOH). M.p. = 207-208 ºC.

^{1}H NMR (300 MHz, $CDCl_3$) δ 8.62 (s, 1H, OH), 7.90 (d, *J* = 8.4 Hz, 1H, H_{Ar}), 7.72 (d, *J* = 7.9 Hz, 1H, H_{Ar}), 7.45 (s, 1H, H_{Ar}), 7.30 – 6.96 (m, 10H, H_{Ar}), 6.67 (d, *J* = 7.4 Hz, 2H, H_{Ar}), 6.03 (d, *J* = 8.1 Hz, NH), 4.46 (d, *J* = 14.3 Hz, 1H), 4.34 (d, *J* = 14.3 Hz, 1H), 4.20 (s, 1H), 3.62 – 3.40 (m, 1H), 1.64 – 0.55 (m, 10H).

^{13}C {^{1}H} NMR (75 MHz, $CDCl_3$) δ 169.7 (CO), 159.7 (CO), 140.8 (Cq_{Ar}), 137.5 (Cq_{Ar}), 135.1 (Cq_{Ar}), 129.3 (CH_{Ar}), 129.0 (CH_{Ar}), 128.6 (CH_{Ar}), 128.4 (Cq_{Ar}), 128.3 (CH_{Ar}), 127.8 (Cq_{Ar}), 125.6 (CH_{Ar}), 125.5 (CH_{Ar}), 122.3 (CH_{Ar}), 121.3 (CH_{Ar}), 114.9 (CH_{Ar}), 109.0 (CH_{Ar}), 90.2 (Cq), 66.0 (CH), 50.9 (CH_2), 48.8 (CH), 32.0 (CH_2), 31.8 (CH_2), 25.1 (CH_2), 24.5 (CH_2), 24.3 (CH_2).

HRMS (ESI) calculated for ($C_{31}H_{32}N_3O_3$) ($[M+H]^+$): 494.2438; found: 494.2430.

(3*R,4*R**)-*N*-Cyclohexyl-4-(4-fluorophenyl)-4-hydroxy-1-oxo-1,2,3,4-tetrahydropyrazino[1,2-*a*]indole-3-carboxamide. (9i)**

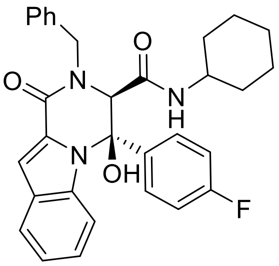

White Solid (58%, 593 mg) (crystallized in iPrOH). M.p. = 206-207 ºC.

^{1}H NMR (300 MHz, $CDCl_3$) δ 8.73 (s, 1H, OH), 7.90 (d, *J* = 8.2 Hz, 1H, H_{Ar}), 7.72 (d, *J* = 8.2 Hz, 1H, H_{Ar}), 7.44 (s, 1H, H_{Ar}), 7.30 – 7.10 (m, 5H, H_{Ar}), 7.04 – 6.90 (m, 2H, H_{Ar}), 6.83 – 6.64 (m, 2H, H_{Ar}), 6.63 – 6.45 (m, 3H, H_{Ar}), 4.82 (d, *J* = 14.3 Hz, 1H), 4.21 (s+d, *J* = 14.3 Hz, 2H), 3.68 – 3.50 (m, 1H), 1.76 – 0.72 (m, 10H).

^{13}C {^{1}H} NMR (75 MHz, $CDCl_3$) δ 169.6 (CO), 162.9 (d, *J* = 248.0 Hz, Cq_{Ar}), 159.3 (CO), 137.5 (Cq_{Ar}), 136.8 (d, *J* = 3.2 Hz, Cq_{Ar}), 134.9 (Cq_{Ar}), 129.00 (d, *J* = 13.3 Hz, CH_{Ar}), 128.9 (CH_{Ar}), 128.3 (Cq_{Ar}), 128.2 (CH_{Ar}), 127.8 (Cq_{Ar}), 127.5 (CH_{Ar}), 127.4 (CH_{Ar}), 125.8 (CH_{Ar}), 122.4 (CH_{Ar}), 121.5 (CH_{Ar}), 115.37 (d, *J* = 21.9 Hz, CH_{Ar}), 114.9 (CH_{Ar}), 109.1 (CH_{Ar}), 89.7 (Cq), 65.6 (CH), 50.4 (CH_2), 49.0 (CH), 32.0 (CH_2), 31.9 (CH_2), 25.1 (CH_2), 24.6 (CH_2), 24.4 (CH_2).

^{19}F NMR (282 MHz, $CDCl_3$) δ -113.02 – -113.14 (m).

HRMS (ESI) calculated for ($C_{31}H_{31}FN_3O_3$) ($[M+H]^+$): 512.2344; found: 512.2333.

(3*R,4*R**)-N,2-di-*tert*-Butyl-4-hydroxy-1-oxo-4-phenyl-1,2,3,4-tetrahydropyrazino[1,2-*a*]indole-3-carboxamide. (9j)**

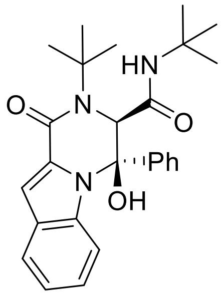

Pink Solid (65%, 277 mg) (crystallized in iPrOH). M.p. = 201 – 202 ºC.

^{1}H NMR (300 MHz, $CDCl_3$) δ 8.73 (s, 1H, OH), 7.88 (d, *J* = 8.4 Hz, 1H, H_{Ar}), 7.74 – 7.35 (m, 4H, H_{Ar}), 7.27 – 7.08 (m, 3H, H_{Ar}), 6.84 – 6.77 (m, 2H, H_{Ar}), 6.44 (s, 1H, NH), 4.50 (s, 1H, CH), 1.20 (s, 9H, CH_3), 1.16 (s, 9H, CH_3).

^{13}C {^{1}H} NMR (75 MHz, $CDCl_3$) δ 171.9 (CO), 160.5 (CO), 141.1 (Cq_{Ar}), 136.7 (Cq_{Ar}), 130.0 (Cq_{Ar}), 129.3 (CH_{Ar}), 129.1 (CH_{Ar}), 128.6 (CH_{Ar}), 127.8 (Cq_{Ar}), 125.9 (CH_{Ar}), 125.2 (CH_{Ar}), 122.2 (CH_{Ar}), 121.1 (CH_{Ar}), 114.6 (CH_{Ar}), 108.1 (CH_{Ar}), 90.6 (Cq), 64.7 (CH), 58.1 (Cq), 52.8 (Cq), 28.3 (CH_3), 28.2 (CH_3).

HRMS (ESI) calculated for ($C_{26}H_{32}N_3O_3$) ($[M+H]^+$): 434.2438; found: 434.2431.

(3*R,4*R**)-2-(3-Bromopropyl)-*N*-(*tert*-butyl)-4-hydroxy-1-oxo-4-phenyl-1,2,3,4-tetrahydropyrazino[1,2-*a*]indole-3-carboxamide. (9k)**

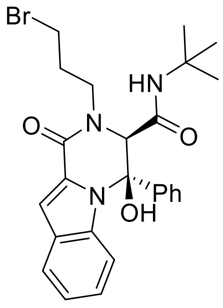

Yellow oil (22%, 219 mg). Rf = 0.43 (hexane/ethyl acetate 2:1).

^{1}H NMR (300 MHz, $CDCl_3$) δ 8.48 (s, 1H, OH), 7.87 (d, *J* = 8.3 Hz, 1H, H_{Ar}), 7.71 (d, *J* = 7.9 Hz, 1H, H_{Ar}), 7.43 (s, 1H, H_{Ar}), 7.30 – 7.10 (m, 5H, H_{Ar}), 6.80 (d, *J* = 7.3 Hz, 2H, H_{Ar}), 6.05 (s, 1H, NH), 4.23 (dt, *J* = 13.8, 6.9 Hz, 1H), 4.17 (s, 1H), 2.93 – 2.77 (m, 2H), 2.55 – 2.44 (m, 1H), 1.55 – 1.43 (m, 2H), 1.19 (s, 9H).

^{13}C {^{1}H} NMR (75 MHz, $CDCl_3$) δ 170.1 (CO), 159.9 (CO), 141.2 (Cq_{Ar}), 137.6 (Cq_{Ar}), 129.5 (Cq_{Ar}), 128.8 (CH_{Ar}), 128.4 (CH_{Ar}), 127.8 (Cq_{Ar}), 125.8 (Cq_{Ar}), 125.7 (CH_{Ar}), 122.4 (CH_{Ar}), 121.5 (CH_{Ar}), 114.9 (CH_{Ar}), 108.9 (CH_{Ar}), 89.9 (Cq), 67.9 (CH), 53.0 (Cq), 45.3 (CH_2), 30.7 (CH_2), 29.9 (CH_2), 28.3 (CH_3).

HRMS (ESI) calculated for ($C_{25}H_{29}BrN_3O_3$) ($[M+H]^+$): 498.1387; found: 498.1389.

(3*R,4*R**)-*N*-Cyclohexyl-4-hydroxy-2-(2-nitrobenzyl)-1-oxo-4-phenyl-1,2,3,4-tetrahydropyrazino[1,2-*a*]indole-3-carboxamide. (9l)**

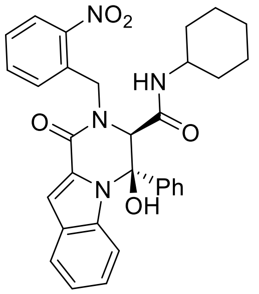

Pale brown solid (72%, 737 mg) (crystallized in iPrOH). M.p. = 222 – 223 ºC.

^{1}H NMR (300 MHz, $CDCl_3$) δ 8.48 (s, 1H, OH), 7.92 – 7.83 (m, 2H, H_{Ar}), 7.78 – 7.66 (m, 1H, H_{Ar}), 7.48 (s, 1H, H_{Ar}), 7.39 – 7.02 (m, 7H, H_{Ar}), 6.69 (d, *J* = 7.8 Hz, 1H, H_{Ar}), 6.60 (d, *J* = 7.6 Hz, 2H, H_{Ar}), 6.32 (d, *J* = 8.3 Hz, 1H, NH), 5.32 (d, *J* = 15.5 Hz, 1H), 4.51 (d, *J* = 15.5 Hz, 1H), 4.45 (s, 1H), 3.76 – 3.61 (m, 1H), 1.89 – 0.81 (m, 10H).

^{13}C {^{1}H} NMR (75 MHz, $CDCl_3$) δ 169.4 (CO), 160.3 (CO), 148.8 (Cq_{Ar}), 141.3 (Cq_{Ar}), 133.9 (Cq_{Ar}), 131.1 (Cq_{Ar}), 130.2 (Cq_{Ar}), 129.2 (CH_{Ar}), 128.8 (CH_{Ar}), 128.7 (CH_{Ar}), 128.1 (CH_{Ar}), 127.9 (Cq_{Ar}), 126.0 (CH_{Ar}), 125.8 (CH_{Ar}), 124.9 (CH_{Ar}), 122.6 (CH_{Ar}), 121.7 (CH_{Ar}), 121.5 (CH_{Ar}), 115.2 (CH_{Ar}), 109.6 (CH_{Ar}), 90.0 (Cq), 66.7 (CH), 49.2 (CH_2), 45.6 (CH), 32.3 (CH_2), 25.3 (CH_2), 24.6 (CH_2), 24.5 (CH_2).

HRMS (ESI) calculated for ($C_{31}H_{30}N_4O_5Na$) ($[M+Na]^+$): 561.2108; found: 561.2110.

(3*R,4*R**)-*N*-Cyclohexyl-4-(4-fluorophenyl)-4-hydroxy-2-(2-nitrobenzyl)-1-oxo-1,2,3,4-tetrahydropyrazino[1,2-*a*]indole-3-carboxamide. (9m)**

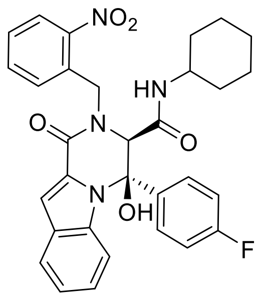

Orange solid (75%, 834 mg) (crystallized in iPrOH). M.p. = 110 – 111ºC.

^{1}H NMR (300 MHz, $CDCl_3$) δ 8.54 (s, 1H, OH), 7.84 (d, *J* = 8.3 Hz, 2H, H_{Ar}), 7.72 (d, *J* = 7.9 Hz, 1H, H_{Ar}), 7.46 (s, 1H, H_{Ar}), 7.39 – 7.31 (m, 1H, H_{Ar}), 7.25 – 7.13 (m, 3H, H_{Ar}), 6.87 – 6.79 (m, 1H, H_{Ar}), 6.73 – 6.65 (m, 2H, H_{Ar}), 6.58 – 6.49 (m, 2H, H_{Ar}), 6.35 (d, *J* = 8.2 Hz, 1H, NH), 5.37 (d, *J* = 15.1 Hz, 1H), 4.47 (d, *J* = 15.1 Hz, 1H), 4.45 (s, 1H), 3.76 – 3.60 (m, 1H), 2.04 – 0.79 (m, 10H).

^{13}C {^{1}H} NMR (75 MHz, $CDCl_3$) δ 169.3, 160.1, 148.8, 137.7, 137.2, 133.7, 131.5, 130.1, 128.9, 128.0, 127.9, 127.8, 127.7, 126.1, 124.8, 122.6, 121.7, 115.6, 115.3, 115.0, 109.7, 89.5, 66.5, 49.2, 44.9, 33.1, 32.3, 32.3, 25.5, 25.2, 24.8, 24.6, 24.5.

^{19}F NMR (282 MHz, $CDCl_3$) δ -112.86 (tt, *J* = 8.5, 5.2 Hz).

HRMS (ESI) calculated for ($C_{31}H_{29}FN_4O_5Na$) ($[M+Na]^+$): 489.2132; found: 489.2135.

(3*R,4*R**)-*N*-(*tert*-Butyl)-4-hydroxy-4-(4-methoxyphenyl)-2-(2-nitrobenzyl)-1-oxo-1,2,3,4-tetrahydropyrazino[1,2-*a*]indole-3-carboxamide. (9n)**

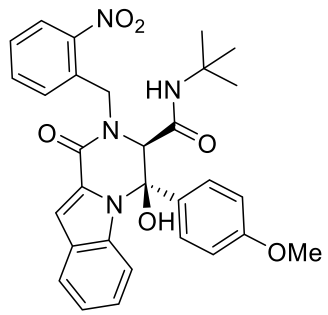

Pale brown solid (76%, 824 mg) (crystallized in iPrOH). M.p. = 187 – 188 ºC.

^{1}H NMR (300 MHz, $CDCl_3$) δ 8.25 (s, 1H, OH), 7.92 – 7.77 (m, 2H, H_{Ar}), 7.76 – 7.67 (m, 1H, H_{Ar}), 7.47 (s, 1H, H_{Ar}), 7.39 – 7.09 (m, 4H, H_{Ar}), 6.95 – 6.84 (m, 1H, H_{Ar}), 6.54 (s, 4H, H_{Ar}), 6.03 (s, 1H, NH), 5.25 (d, *J* = 15.3 Hz, 1H), 4.54 (d, *J* = 15.1 Hz, 1H), 4.37 (s, 1H), 3.71 (s, 3H), 1.13 (s, 9H).

^{13}C {^{1}H} NMR (75 MHz, $CDCl_3$) δ 160.2 (CO), 159.9 (CO), 148.6 (Cq_{Ar}), 137.6 (Cq_{Ar}), 133.7 (CH_{Ar}), 133.1 (Cq_{Ar}), 131.5 (CH_{Ar}), 130.3 (Cq_{Ar}), 128.6 (CH_{Ar}), 128.0 (Cq_{Ar}), 127.7 (Cq_{Ar}), 126.9 (CH_{Ar}), 126.8 (Cq_{Ar}), 125.7 (CH_{Ar}), 124.7 (CH_{Ar}), 122.4 (CH_{Ar}), 121.4 (CH_{Ar}), 114.8 (CH_{Ar}), 113.8 (CH_{Ar}), 109.2 (CH_{Ar}), 89.6 (Cq), 66.9 (CH), 55.2 (CH_3), 52.8 (CH_2), 45.3 (Cq), 28.2 (CH_3).

HRMS (ESI) calculated for ($C_{30}H_{30}N_4O_6Na$) ($[M+Na]^+$): 565.2058; found: 565.2063.

(3*R,4*R**)-*N*-(*tert*-Butyl)-4-hydroxy-2-(2-nitrobenzyl)-1-oxo-4-(4-(trifluoromethyl)phenyl)-1,2,3,4-tetrahydropyrazino[1,2-*a*]indole-3-carboxamide. (9o)**

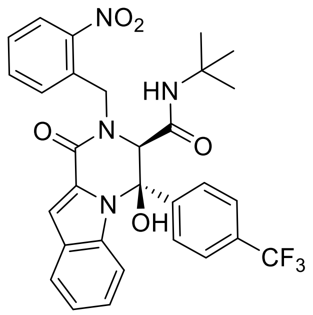

Pale brown solid (71%, 823 mg) (crystallized in iPrOH). M.p. = 221 – 222 ºC.

^{1}H NMR (300 MHz, $CDCl_3$) δ 8.61 (s, 1H, OH), 7.85 – 7.79 (m, 2H, H_{Ar}), 7.77 – 7.71 (m, 1H, H_{Ar}), 7.52 (s, 1H, H_{Ar}), 7.36 – 7.07 (m, 6H, H_{Ar}), 6.87 (dd, *J* = 7.8, 1.2 Hz, 1H, H_{Ar}), 6.67 (d, *J* = 8.1 Hz, 2H, H_{Ar}), 6.07 (s, 1H, NH), 5.40 (d, *J* = 14.8 Hz, 1H), 4.44 (s+d, *J* = 15.8 Hz, 2H), 1.19 (s, 9H).

^{13}C {^{1}H} NMR (75 MHz, $CDCl_3$) δ 169.8 (CO), 159.9 (CO), 148.8 (Cq_{Ar}), 145.1 (Cq_{Ar}), 137.6 (Cq_{Ar}), 133.8 (CH_{Ar}), 131.8 (CH_{Ar}), 129.8 (CH_{Ar}), 129.2 (CH_{Ar}), 127.9 (Cq_{Ar}), 127.7 (Cq_{Ar}), 126.2 (CH_{Ar}), 125.5 (q, J = 3.9 Hz, H_{Ar}), 124.7 (CH_{Ar}), 122.7 (CH_{Ar}), 121.8 (CH_{Ar}), 114.8 (CH_{Ar}), 110.0 (CH_{Ar}), 89.6 (Cq), 65.9 (CH), 53.2 (Cq), 44.4 (CH_2), 28.3 (CH_3).

^{19}F NMR (282 MHz, $CDCl_3$) δ -62.67 (s).

HRMS (ESI) calculated for ($C_{30}H_{27}F_3N_4O_5Na$) ($[M+Na]^+$): 603.1826; found: 603.1833.

1-Benzyl-*N*-cyclohexyl-6-oxo-3,4-diphenyl-1,4,5,6-tetrahydropyrazine-2-carboxamide. (10a)

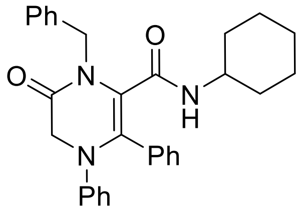

Pink solid (54%, 519 mg) (crystallized in iPrOH). M. p. = 206 – 207 ºC.

^{1}H NMR (300 MHz, $CDCl_3$) δ 7.42 – 6.98 (m, 13H, H_{Ar}), 6.89 – 6.63 (m, 2H, H_{Ar}), 5.20 (s, 2H), 5.09 (d, J = 7.8 Hz, 1H, NH), 4.46 (s, 2H), 3.58 – 3.28 (m, 2H), 1.96 – 1.50 (m, 4H), 1.49 – 0.84 (m, 2H), 0.62 – 0.43 (m, 2H).

^{13}C {^{1}H} NMR (75 MHz, $CDCl_3$) δ 163.5 (CO), 162.4 (CO), 144.2 (Cq), 137.8 (Cq), 133.2 (Cq), 132.3 (Cq), 130.0 (CH_{Ar}), 128.9 (CH_{Ar}), 128.7 (CH_{Ar}), 128.3 (CH_{Ar}), 128.3 (CH_{Ar}), 128.0 (CH_{Ar}), 127.1 (CH_{Ar}), 122.9 (CH_{Ar}), 122.1 (CH_{Ar}), 121.1 (Cq), 55.0 (CH_2), 48.3 (CH), 46.0 (CH_2), 31.9 (CH_2), 25.3 (CH_2), 24.4 (CH_2).

HRMS (ESI) calculated for ($C_{30}H_{32}N_3O_2$) ($[M+H]^+$): 466.2489; found: 466.2481.

1-Benzyl-*N*-cyclohexyl-3-(4-fluorophenyl)-6-oxo-4-phenyl-1,4,5,6-tetrahydropyrazine-2-carboxamide. (10b)

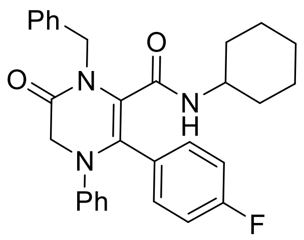

White solid (49%, 472 mg) (crystallized in iPrOH). M.p.= 204 – 205 ºC.

^{1}H NMR (300 MHz, $CDCl_3$) δ 7.37 – 7.26 (m, 8H, H_{Ar}), 7.13 – 7.04 (m, 2H, H_{Ar}), 6.92 – 6.83 (m, 2H, H_{Ar}), 6.74 – 6.68 (m, 2H, H_{Ar}), 5.17 (s, 2H), 5.11 (d, J = 8.3 Hz, 1H, NH), 4.47 (s, 2H), 3.53 – 3.38 (m, 1H), 1.52 – 1.31 (m, 5H), 1.27 – 1.08 (m, 2H), 1.05 – 0.91 (m, 1H), 0.70 – 0.52 (m, 2H).

^{13}C {^{1}H} NMR (75 MHz, $CDCl_3$) δ 163.6, 162.4, 147.3, 132.0, 131.9, 130.0, 129.3, 129.0, 128.8, 128.5, 128.1, 127.4, 123.2, 122.3, 115.6, 115.3, 113.1, 55.2, 48.5, 46.3, 32.2, 25.4, 24.6.

^{19}F NMR (282 MHz, $CDCl_3$) δ -111.43 – -111.61 (m).

HRMS (ESI) calculated for ($C_{30}H_{31}FN_3O_2$) ($[M+H]^+$): 484.2395; found: 484.2397.

(3*R,4*R**)-*N*-(*tert*-Butyl)-4-hydroxy-2-((1*S*)-methylbenzyl)-1-oxo-4-phenyl-1,2,3,4-tetrahydropyrazino[1,2-*a*]indole-3-carboxamide. (11)**

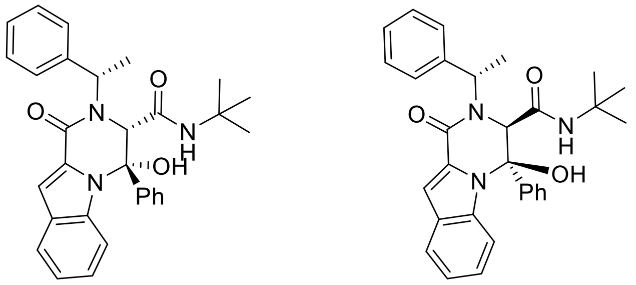

Diastereomer 1. White solid (40%, 383 mg). M.p. = 193-194 ºC. $[\alpha]_D^{20}$ = +142 (c = 0.19, CH_2Cl_2). Rf = 0.83 (hexane/ethyl acetate 2:1).

^{1}H NMR (300 MHz, $CDCl_3$) δ 8.87 (s, 1H, OH), 7.90 (d, J = 8.4 Hz, 1H, H_{Ar}), 7.70 (d, *J* = 8.0 Hz, 1H, H_{Ar}), 7.48 – 7.08 (m, 11H, H_{Ar}), 6.97 – 6.89 (m, 2H, H_{Ar}), 5.92 (q, *J* = 7.3 Hz, 1H), 5.41 (s, 1H, NH), 4.19 (s, 1H), 0.74 (s, 9H), 0.57 (d, *J* = 7.3 Hz, 3H).

^{13}C {^{1}H} NMR (75 MHz, $CDCl_3$) δ 170.4 (CO), 159.7 (CO), 141.3 (Cq_{Ar}), 139.5 (Cq_{Ar}), 137.1 (Cq_{Ar}), 129.7 (CH_{Ar}), 129.3 (CH_{Ar}), 129.0 (CH_{Ar}), 128.8 (Cq_{Ar}), 128.7 (CH_{Ar}), 128.2 (CH_{Ar}), 127.9 (Cq_{Ar}), 126.3 (CH_{Ar}), 125.5 (CH_{Ar}), 122.3 (CH_{Ar}), 121.3 (CH_{Ar}), 114.9 (CH_{Ar}), 108.6 (CH_{Ar}), 90.7 (Cq), 61.8 (CH), 52.1 (Cq), 50.8 (CH), 27.5 (CH_3), 15.5 (CH_3).

Diastereomer 2. White solid (42%, 406 mg). M. p. = 224-225 ºC. $[\alpha]_D^{20}$ = +188 (c = 0.14, CH_2Cl_2). Rf = 0.26 (hexane/ethyl acetate 2:1).

^{1}H NMR (300 MHz, $CDCl_3$) δ 8.01 (s, 1H, OH), 7.83 (d, *J* = 8.4 Hz, 1H, H_{Ar}), 7.71 (d, *J* = 7.3 Hz, 1H, H_{Ar}), 7.46 (s, 1H, H_{Ar}), 7.29 – 6.86 (m, 10H, H_{Ar}), 6.45 (d, *J* = 7.3 Hz, 2H, H_{Ar}), 6.07 (s, 1H, NH), 5.39 (q, *J* = 7.2 Hz, 1H), 4.10 (s, 1H), 1.50 (d, *J* = 7.2 Hz, 3H), 1.09 (s, 9H).

^{13}C {^{1}H} NMR (75 MHz, $CDCl_3$) δ 171.0 (CO), 159.5 (CO), 140.7 (Cq_{Ar}), 138.4 (Cq_{Ar}), 137.3 (Cq_{Ar}), 129.0 (Cq_{Ar}), 128.8 (CH_{Ar}), 128.6 (CH_{Ar}), 128.0 (CH_{Ar}), 127.9 (CH_{Ar}), 127.8 (Cq_{Ar}), 125.6 (CH_{Ar}), 125.5 (CH_{Ar}), 122.4 (CH_{Ar}), 121.3 (CH_{Ar}), 114.8 (CH_{Ar}), 108.6 (CH_{Ar}), 90.1 (Cq), 65.2 (CH), 55.6 (CH), 52.8 (Cq), 28.2 (CH_3), 17.6 (CH_3).

2.4. General procedure for the synthesis of pirazinoquinazolines 13 and 14.

Piperazinone (0.5 mmol, 1 equiv), tin (II) chloride (5 mmol, 10 equiv) and HCl (1.5 mmol, 3 equiv) were added to 30 mL of *n*-butanol. The mixture is then heated to 120 °C and stirred for 1 hour. Neither the piperazinone or the tin chloride dissolve in methanol until the mixture is heated to 120 °C. After that, the solvent is removed and the crude is

redissolved in dichloromethane (50 mL) and washed with a saturated solution of sodium carbonate (3 × 40 mL). The organic phase was dried over anhydrous sodium sulfate, filtered and concentrated to dryness. The product was purified by flash column chromatography (SiO_2, hexane/ethyl acetate 2:1).

(5*R,6*R**)-*N*-Cyclohexyl-5-hydroxy-5-phenyl-5,6-dihydro-8*H*-pyrrolo[2',1':3,4]pyrazino[2,1-*b*]quinazoline-6-carboxamide. (13a)**

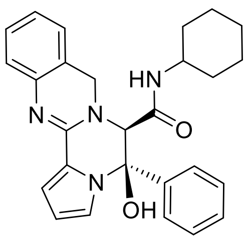

Brown oil (70%, 308 mg). Rf = 0.15 (hexane/ethyl acetate 2:1).

^{1}H NMR (300 MHz, $CDCl_3$) δ 8.87 (s, 1H, OH), 7.80 (d, *J* = 7.5 Hz, 1H, NH), 7.37 – 7.32 (m, 3H, H_{Ar}), 7.24 – 7.20 (m, 1H, H_{Ar}), 7.18 – 7.10 (m, 1H, H_{Ar}), 7.09 – 7.05 (m, 1H, H_{Ar}), 6.96 – 6.82 (m, 2H, H_{Ar}), 6.75 – 6.66 (m, 1H, H_{Ar}), 6.64 – 6.59 (m, 1H, H_{Ar}), 6.39 – 6.32 (m, 1H, H_{Ar}), 5.17 (d, *J* = 15.0 Hz, 1H), 4.63 (s, 2H), 4.01 (s, 1H), 3.51 – 3.27 (m, 1H), 2.85 (d, *J* = 15.0 Hz, 1H), 1.68 – 0.74 (m, 10H).

^{13}C {^{1}H} NMR (75 MHz, $CDCl_3$) δ 170.4 (CO), 160.1 (CN), 144.8 (Cq_{Ar}), 141.4 (Cq_{Ar}), 131.5 (CH_{Ar}), 130.3 (CH_{Ar}), 129.3 (CH_{Ar}), 128.9 (CH_{Ar}), 125.5 (CH_{Ar}), 123.3 (Cq_{Ar}), 122.5 (CH_{Ar}), 119.1 (CH_{Ar}), 118.8 (Cq_{Ar}), 116.2 (CH_{Ar}), 115.9 (CH_{Ar}), 111.0 (CH_{Ar}), 89.1 (Cq), 64.4 (CH), 49.2 (CH_2), 32.3 (CH_2), 31.1 (CH_2), 25.4 (CH_2), 25.0 (CH_2), 24.9 (CH_2).

HRMS (ESI) calculated for ($C_{27}H_{29}N_4O_2$) ($[M+H]^+$): 441.2285; found: 441.2289.

(5*R,6*R**)-*N*-(*tert*-Butyl)-5-hydroxy-5-(4-(trifluoromethyl)phenyl)-5,6-dihydro-8H-pyrrolo[2',1':3,4]pyrazino[2,1-*b*]quinazoline-6-carboxamide. (13b)**

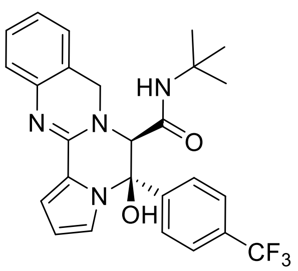

Orange oil (70%, 337 mg). Rf = 0.17 (hexane/ethyl acetate 2:1).

^{1}H NMR (300 MHz, $CDCl_3$) δ 8.87 (s, 1H, OH), 7.57 (d, *J* = 8.7 Hz, 2H, H_{Ar}), 7.22 – 7.08 (m, 3H, H_{Ar}), 6.99 (d, *J* = 8.7 Hz, 2H, H_{Ar}), 6.88 (d, *J* = 6.3 Hz, 1H, H_{Ar}), 6.68 (t, *J* = 7.4 Hz, 1H, H_{Ar}), 6.57 (d, *J* = 7.9 Hz, 1H, H_{Ar}), 6.39 – 6.34 (m, 1H, H_{Ar}), 5.06 (d, *J* = 14.9 Hz, 1H, H_{Ar}), 4.58 (s, 1H, NH), 3.98 (s, 1H), 3.05 (d, *J* = 14.9 Hz, 1H), 1.00 (s, 9H).

^{13}C {^{1}H} NMR (75 MHz, $CDCl_3$) δ 170.3, 159.6, 145.4, 144.9, 131.7, 131.6, 130.4, 126.1, 125.9, 125.9, 123.1, 122.3, 118.7, 118.0, 116.2, 115.7, 111.3, 95.9, 88.5, 64.4, 52.8, 49.0, 27.7.

^{19}F NMR (282 MHz, $CDCl_3$) δ -62.76 (s).

HRMS (ESI) calculated for ($C_{26}H_{26}F_3N_4O_2$) ($[M+H]^+$): 483.2002; found: 483.2005.

(6*R,7*R**)-*N*-Cyclohexyl-6-hydroxy-6-phenyl-6,7-dihydro-9H-indolo[2',1':3,4]pyrazino[2,1-*b*]quinazoline-7-carboxamide. (13c)**

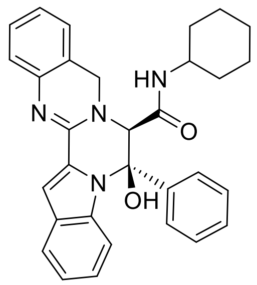

Brown oil (72%, 353 mg). Rf = 0.15 (hexane/ethyl acetate 2:1).

^{1}H NMR (300 MHz, $CDCl_3$) δ 7.79 – 6.56 (m, 15H, H_{Ar}+OH), 5.93 (d, *J* = 8.5 Hz, 1H), 5.55 (s, 1H), 5.48 – 5.35 (m, 2H), 3.42 – 3.27 (m, 1H), 1.81 – 0.79 (m, 9H), 0.50 – 0.28 (m, 1H).

^{13}C {^{1}H} NMR (75 MHz, $CDCl_3$) δ 160.9 (CO), 157.6 (CN), 133.9 (Cq_{Ar}), 132.1 (CH_{Ar}), 132.1 (CH_{Ar}), 131.6 (Cq_{Ar}), 131.2 (CH_{Ar}), 130.5 (CH_{Ar}), 129.4 (CH_{Ar}), 129.4 (CH_{Ar}), 129.1 (Cq_{Ar}), 127.7 (Cq_{Ar}), 124.1 (CH_{Ar}), 122.6 (CH_{Ar}), 122.6 (CH_{Ar}), 122.2 (Cq_{Ar}), 121.4 (Cq_{Ar}), 114.3 (CH_{Ar}), 106.0 (CH_{Ar}), 48.8 (CH), 43.9 (CH), 43.8 (CH_2), 31.5 (CH_2), 25.3 (CH_2), 24.5 (CH_2).

HRMS (ESI) calculated for ($C_{21}H_{31}N_4O_2$) ($[M+H]^+$): 491.2442; found: 491.2446.

(6*R,7*R**)-*N*-Cyclohexyl-6-(4-fluorophenyl)-6-hydroxy-6,7-dihydro-9H-indolo[2',1':3,4]pyrazino[2,1-*b*]quinazoline-7-carboxamide. (13d)**

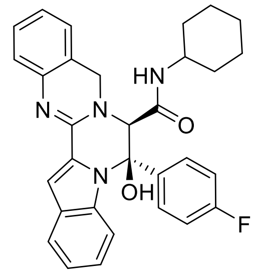

Yellow oil (68%, 345 mg). Rf = 0.16 (hexane/ethyl acetate 2:1).

^{1}H NMR (300 MHz, $CDCl_3$) δ 7.80 – 7.74 (m, 1H, H_{Ar}), 7.58 (s, 1H, OH), 7.54 – 7.47 (m, 2H, H_{Ar}), 7.31 – 7.13 (m, 5H, H_{Ar}), 7.10 – 6.93 (m, 3H, H_{Ar}), 6.66 – 6.56 (m, 2H, H_{Ar}), 6.04 – 5.97 (m, 1H), 5.41 (s, 2H), 5.04 (d, *J* = 7.2 Hz, 1H, NH), 3.45 – 3.26 (m, 1H), 1.55 – 0.80 (m, 9H), 0.47 – 0.30 (m, 1H).

^{13}C {^{1}H} NMR (75 MHz, $CDCl_3$) δ 160.8, 157.4, 146.1, 133.8, 133.4, 133.3, 131.9, 129.4, 129.2, 127.8, 127.7, 124.3, 122.8, 122.7, 120.9, 120.4, 117.7, 116.8, 116.6, 115.9, 114.0, 106.3, 95.9, 48.9, 43.8, 31.7, 25.4, 24.6.

^{19}F NMR (282 MHz, $CDCl_3$) δ -109.28 (tt, *J* = 8.5, 5.2 Hz).

HRMS (ESI) calculated for ($C_{31}H_{30}N_4O_2F$) ($[M+H]^+$): 509.2347; found: 509.2349.

(6*R,7*R**)-*N*-(*tert*-Butyl)-6-hydroxy-6-(4-methoxyphenyl)-6,7-dihydro-9H-indolo[2',1':3,4]pyrazino[2,1-*b*]quinazoline-7-carboxamide. (13e)**

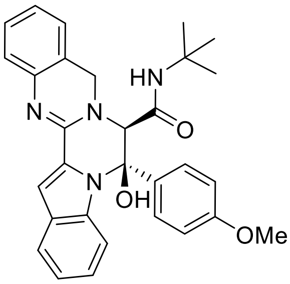

Yellow oil (69%, 340 mg). Rf = 0.16 (hexane/ethyl acetate 2:1).

^{1}H NMR (300 MHz, DMSO-d_6) δ 7.75 (d, J = 7.9 Hz, 1H, H_{Ar}), 7.64 (s, 1H, OH), 7.49 (d, J = 7.9 Hz, 2H, H_{Ar}), 7.39 (s, 1H, H_{Ar}), 7.17 – 6.83 (m, 6H, H_{Ar}), 6.55 (d, J = 8.3 Hz, 1H, H_{Ar}), 6.41 (t, J = 7.3 Hz, 1H, H_{Ar}), 6.07 (d, J = 8.8 Hz, 1H, H_{Ar}), 5.10 (s, 1H, NH), 4.92 (s, 1H), 3.78 (s, 3H), 0.75 (s, 9H).

^{13}C {^{1}H} NMR: The solubility of the product is too low.

HRMS (ESI) calculated for ($C_{30}H_{31}N_4O_3$) ($[M+H]^+$): 495.2391; found: 495.2399.

(6*R,7*R**)-*N*-(*tert*-Butyl)-6-hydroxy-6-(4-(trifluoromethyl)phenyl)-6,7-dihydro-9H-indolo[2',1':3,4]pyrazino[2,1-*b*]quinazoline-7-carboxamide. (13f)**

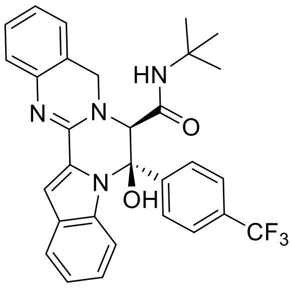

White solid (68%, 362 mg). M.p.= 139 – 140 ºC. Rf = 0.17 (hexane/ethyl acetate 2:1).

^{1}H NMR (300 MHz, $CDCl_3$) δ 9.15 (s, 1H, OH), 7.94 – 7.89 (m, 1H, H_{Ar}), 7.74 (d, J = 8.6 Hz, 1H, H_{Ar}), 7.54 (d, J = 8.4 Hz, 2H, H_{Ar}), 7.47 (s, 1H, H_{Ar}), 7.30 – 7.22 (m, 1H, H_{Ar}), 7.21 – 7.12 (m, 3H, H_{Ar}), 7.01 – 6.88 (m, 2H, H_{Ar}), 6.72 (t, J = 7.4 Hz, 1H, H_{Ar}), 6.60 (d, J = 8.0 Hz, 1H, H_{Ar}), 5.12 (d, J = 14.8 Hz, 1H), 4.59 (s, 1H, NH), 4.06 (s, 1H), 3.14 (d, J = 14.8 Hz, 1H), 0.97 (s, 9H).

^{13}C {^{1}H} NMR (75 MHz, $CDCl_3$) δ 170.1, 160.3, 144.9, 144.7, 137.5, 131.6, 130.5, 128.1, 127.7, 126.1, 126.0, 125.8, 125.8, 122.5, 121.5, 118.7, 117.6, 115.7, 114.6, 109.5, 90.2, 64.9, 52.8, 49.3, 27.6.

HRMS (ESI) calculated for ($C_{30}H_{28}F_3N_4O_2$) ($[M+H]^+$): 533.2159; found: 533.2165.

***N*-Cyclohexyl-2,3-diphenyl-1,6-dihydro-*2H*-pyrazino[2,1-*b*]quinazoline-4-carboxamide. (14)**

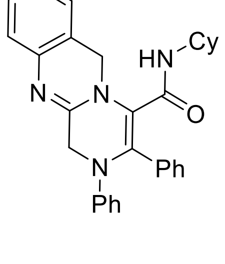

Orange oil (64%, 148 mg). Rf = 0.12 (hexane/ethyl acetate 1:1).

^{1}H NMR (300 MHz, $CDCl_3$) δ 7.53 – 6.92 (m, 12H, H_{Ar}), 6.89 – 6.74 (m, 2H, H_{Ar}), 5.77 (d, J = 7.6 Hz, 1H, NH), 5.10 (s, 2H), 4.51 (s, 2H), 3.74 – 3.57 (m, 1H), 1.77 – 0.64 (m, 10H).

^{13}**C {^{1}H} NMR** (75 MHz, $CDCl_3$) δ 163.0 (Cq), 150.5 (Cq), 148.1 (Cq), 144.5 (Cq), 133.6 (Cq), 129.7 (CH_{Ar}), 129.7 (Cq), 128.9 (CH_{Ar}), 128.8 (CH_{Ar}), 128.6 (CH_{Ar}), 128.5 (CH_{Ar}), 125.9 (CH_{Ar}), 125.4 (CH_{Ar}), 124.0 (CH_{Ar}), 123.5 (Cq), 122.5 (CH_{Ar}), 121.8 (CH_{Ar}), 121.5 (Cq), 54.4 (CH_2), 48.9 (CH), 46.8 (CH_2), 32.4 (CH_2), 25.4 (CH_2), 24.8 (CH_2).

HRMS (ESI) calculated for ($C_{30}H_{30}N_4O$) ($[M+H]^+$): 463.2492; found: 463.2504.

2.5. General procedure for the synthesis of dipyrrolopiperazinones 12.

Arylglyoxal hydrate **1** (2 mmol, 1 equiv) was dissolved in methanol (7 mL), after which the amine **3c** (2 mmol, 1 equiv), was added. The mixture was stirred for 20 minutes at room temperature and then the corresponding acid **2** (2 mmol, 1 equiv) and the isocyanide **4** (2 mmol, 1 equiv) were added. The corresponding mixture was stirred at room temperature for 24 hours after which, 10 mL more of methanol and 650 mg (2 mmol, 1 equiv.) of cesium carbonate were added to the mixture. The mixture was then stirred for one hour at 40 °C. The solvent was then removed under reduced pressure and the residue dissolved in dichloromethane (30 mL). This solution was washed with a 1 M hydrochloric acid aqueous solution (2 × 50 mL) and a saturated sodium carbonate aqueous solution (1 × 50 mL). The organic phase was dried over anhydrous sodium sulfate, filtered and concentrated to dryness. The product **12** was purified by flash column chromatography (SiO_2, hexane/ethyl acetate from 6:1 to 1:1).

(10*R,10*aR**)-*N*-Cyclohexyl-10-hydroxy-5-oxo-10-phenyl-2,3-dihydro-1*H*,5*H*-dipyrrolo[1,2-*a*:1',2'-*d*]pyrazine-10a(10*H*)-carboxamide. (12a)**

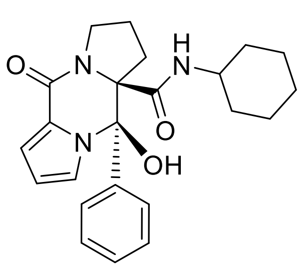

Black solid (93%, 730 mg). M.p. = 182 – 183 ºC. Rf = 0.23 (hexane/ethyl acetate 2:1).

^{1}**H NMR** (300 MHz, $CDCl_3$) δ 8.89 (s, 1H, OH), 7.41 – 7.22 (m, 3H, H_{Ar}), 7.19 – 7.14 (m, 2H, H_{Ar}), 7.10 – 7.08 (m, 1H, H_{Ar}), 7.05 – 6.99 (m, 1H, H_{Ar}), 6.43 (d, J = 8.1 Hz, 1H, NH), 6.28 – 6.24 (m, 1H, H_{Ar}), 3.77 – 3.61 (m, 2H), 3.44 – 3.27 (m, 1H), 2.39 – 2.27 (m, 1H), 2.25 – 2.11 (m, 1H), 2.02 – 1.46 (m, 5H), 1.22 – 0.69 (m, 5H).

^{13}**C {^{1}H} NMR** (75 MHz, $CDCl_3$) δ 175.0 (CO), 158.7 (CO), 138.4 (Cq_{Ar}), 129.3 (CH_{Ar}), 128.4 (CH_{Ar}), 126.8 (CH_{Ar}), 124.9 (Cq_{Ar}), 121.7 (CH_{Ar}), 114.6 (CH_{Ar}), 111.1 (CH_{Ar}), 90.5 (Cq), 71.7 (Cq), 49.0 (CH), 46.0 (CH_2), 33.0 (CH_2), 32.3 (CH_2), 32.2 (CH_2), 25.3 (CH_2), 24.8 (CH_2), 24.5 (CH_2), 22.4 (CH_2).

HRMS (ESI) calculated for ($C_{23}H_{28}N_3O_3$) ($[M+H]^+$): 394.2125; found: 394.2129.

(10*R,10a*R**)-*N*-(*tert*-Butyl)-10-hydroxy-5-oxo-10-phenyl-2,3-dihydro-1*H*,5*H*-dipyrrolo[1,2-*a*:1',2'-*d*]pyrazine-10a(10*H*)-carboxamide. (12b)**

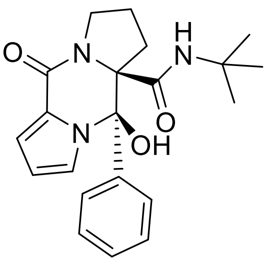

Brown solid (74%, 543 mg). M.p. = 176 – 177 ºC. Rf = 0.22 (hexane/ethyl acetate 2:1).

^{1}H NMR (300 MHz, $CDCl_3$) δ 8.88 (s, 1H, OH), 7.33 – 7.27 (m, 3H, H_{Ar}), 7.20 – 7.13 (m, 2H, H_{Ar}), 7.10 (dd, *J* = 2.7, 1.7 Hz, 1H, H_{Ar}), 7.05 (dd, *J* = 3.8, 1.6 Hz, 1H, H_{Ar}), 6.26 (dd, *J* = 3.8, 2.7 Hz, 1H, H_{Ar}), 6.20 (s, 1H, NH), 3.70 – 3.55 (m, 1H), 3.45 – 3.29 (m, 1H), 2.43 – 2.28 (m, 1H), 2.16 (dt, *J* = 13.7, 6.9 Hz, 1H), 1.73 – 1.54 (m, 1H), 1.18 (s, 9H), 1.13 – 0.97 (m, 1H).

^{13}C {^{1}H} NMR (75 MHz, $CDCl_3$) δ 175.7 (CO), 158.9 (CO), 138.3 (Cq_{Ar}), 129.2 (CH_{Ar}), 128.4 (CH_{Ar}), 126.8 (CH_{Ar}), 124.8 (Cq_{Ar}), 121.7 (CH_{Ar}), 114.7 (CH_{Ar}), 111.1 (CH_{Ar}), 90.5 (Cq), 71.7 (Cq), 52.6 (CH_{Ar}), 46.0 (CH_2), 32.8 (CH_2), 28.3 (CH_3), 22.5 (CH_2).

HRMS (ESI) calculated for ($C_{21}H_{26}N_3O_3$) ($[M+H]^+$): 368.1969; found: 368.1966.

(10*R,10a*R**)-*N*-(*tert*-Butyl)-10-hydroxy-5-oxo-10-(4-fluorophenyl)-2,3-dihydro-1*H*,5*H*-dipyrrolo[1,2-*a*:1',2'-*d*]pyrazine-10a(10*H*)-carboxamide. (12c)**

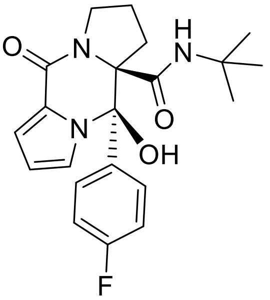

Brown solid (85%, 740 mg). M.p. = 230 – 231 ºC. Rf = 0.23 (hexane/ethyl acetate 2:1).

^{1}H NMR (300 MHz, $CDCl_3$) δ 8.88 (s, 1H, OH), 7.20 – 6.91 (m, 6H, H_{Ar}), 6.27 (dd, *J* = 3.8, 2.7 Hz, 1H, H_{Ar}), 6.09 (s, 1H, NH), 3.70 – 3.56 (m, 1H), 3.46 – 3.33 (m, 1H), 2.36 – 2.14 (m, 2H), 1.74 – 1.58 (m, 1H), 1.18 (s, 9H), 1.16 – 1.10 (m, 1H).

^{13}C {^{1}H} NMR (75 MHz, $CDCl_3$) δ 175.6 (CO), 166.4 (d, ^1J = 227.4 Hz, Cq_{Ar}), 158.7 (CH_{Ar}), 128.9 (d, ^3J = 8.4 Hz, CH_{Ar}), 124.7 (Cq_{Ar}), 121.7 (Cq_{Ar}), 115.4 (d, ^2J = 21.4 Hz, CH_{Ar}), 114.8 (CH_{Ar}), 90.2 (Cq), 71.7 (Cq), 52.7 (Cq), 45.9 (CH_2), 32.8 (CH_2), 28.3 (CH_3), 22.5 (CH_2).

^{19}F NMR (282 MHz, $CDCl_3$) δ -112.64 (tt, *J* = 8.3, 5.2 Hz).

HRMS (ESI) calculated for ($C_{21}H_{25}FN_3O_3$) ($[M+H]^+$): 386.1875; found: 386.1876.

(12*R,12a*R**)-*N*-(*tert*-Butyl)-12-hydroxy-5-oxo-12-phenyl-2,3-dihydro-1*H*,5*H*-pyrrolo[1',2':4,5]pyrazino[1,2-*a*]indole-12a(12*H*)-carboxamide. (12d)**

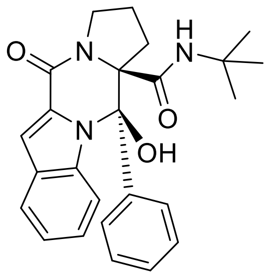

White solid (80%, 667 mg). M.p. = 202 – 203 ºC. Rf = 0.11 (hexane/ethyl acetate 2:1).

^{1}H NMR (300 MHz, $CDCl_3$) δ 9.13 (s, 1H, OH), 7.95 – 7.88 (m, 1H, H_{Ar}), 7.68 – 7.61 (m, 1H, H_{Ar}), 7.41 (s, 1H, H_{Ar}), 7.31 – 7.02 (m, 7H, H_{Ar}), 6.17 (s, 1H, NH), 3.75 – 3.61 (m, 1H), 3.51 – 3.37 (m, 1H), 2.37 (dt, *J* = 14.8, 7.5 Hz, 1H), 2.21 (dt, *J* = 13.7, 6.9 Hz, 1H), 1.65 (dp, *J* = 13.8, 7.3 Hz, 1H), 1.16 (s, 9H), 1.13 – 1.02 (m, 1H).

^{13}C {^{1}H} NMR (75 MHz, $CDCl_3$) δ 175.5 (CO), 159.2 (CO), 138.3 (Cq_{Ar}), 137.1 (Cq_{Ar}), 130.0 (Cq_{Ar}), 129.1 (CH_{Ar}), 128.3 (CH_{Ar}), 128.2 (CH_{Ar}), 127.0 (CH_{Ar}), 125.3 (CH_{Ar}), 122.2 (CH_{Ar}), 121.1 (CH_{Ar}), 114.9 (CH_{Ar}), 107.8 (CH_{Ar}), 92.1 (Cq), 71.8 (Cq), 52.6 (Cq), 46.3 (CH_2), 33.0 (CH_2), 28.3 (CH_3), 22.3 (CH_2).

HRMS (ESI) calculated for ($C_{25}H_{28}N_3O_3$) ($[M+H]^+$): 418.2125; found: 418.2129.

3. X-ray crystallographic data

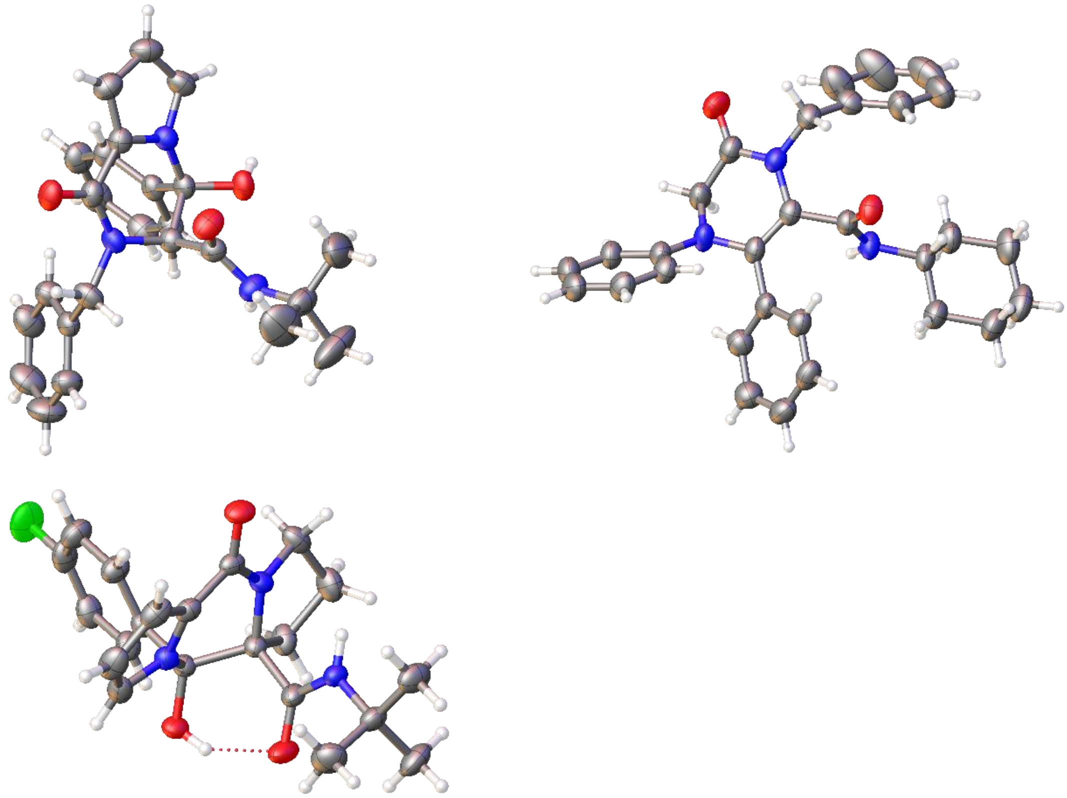

Figure S1. Clockwise, starting from top-left figure. X-Ray molecular structures of compounds **9a**, **10a** and **12c**. In the case of **9a**, a solvent molecule (acetonitrile) has been omitted for the sake of simplicity. The ORTEP plot is at the 40% probability level.

Table S1. Crystal data and refinement details for **9a**, **10a** and **12c**

	**9a**	**10a**	**12c**
Empirical formula	$C_{27}H_{30}N_4O_3$	$C_{30}H_{31}N_3O_2$	$C_{21}H_{24}FN_3O_3$
MW	458.55	465.58	385.43
crystal system	Monoclinic	Monoclinic	Monoclinic
space group	$P2_1/n$	$P2_1/c$	$P2_1/c$
T/K	240(2)	230(2)	220(2)
a/Å	7.4638(5)	13.4401(10)	10.1111(6)
b/Å	19.8712(15)	19.0796(13)	15.7384(9)
c/Å	17.2375(13)	9.9161(8)	12.7944(7)
α/deg	90	90	90
β/deg	97.089(3)	103.793(5)	107.595(2)
γ/deg	90	90	90
V/Å^3	2537.0(3)	2469.5(3)	1940.75(19)
F(000)	976	992	816
Z	4	4	4
λ, Å	1.54178	1.54178	1.54178
D_{calc}/g cm^{-3}	1.201	1.252	1.319
μ/mm^{-1}	0.639	0.623	0.790
θ range/deg	5.15–72.53	3.39–66.83	5.66–73.41
R_{int}	0.0545	0.1082	0.0390
reflections measured	40855	27772	57236
unique reflections	5007	4347	3801
reflections observed	3780	2468	3440
GOF on F^2	1.028	1.012	1.023
$R1^a$	0.0491	0.0596	0.0387
$wR2^b$	0.1365	0.2043	0.1004
Largest ≠ peak & hole/eÅ^{-3}	0.175 and -0.190	0.219 and -0.342	0.304 and -0.208
CCDC number	2355183	2355184	2355185

^{a} $R1 = \Sigma||F_0|-|F_c||/\Sigma|F_0|$. ^{b} $wR2$ (all data) $= \{\Sigma[w(|F_0|^2-|F_c|^2)^2]/\Sigma[w(F_0^4)]\}^{1/2}$

Single crystals were obtained by slow evaporation of a solution of the isolated compound in acetonitrile (**9a**), or in a dichloromethane-methanol (**10a**), or isopropyl ether-ethyl acetate (**12c**) mixture.

Three-dimensional X-ray data were collected on a Bruker D8 VENTURE diffractometer. Data were corrected for absorption effects using the multi-scan method (SADABS).[5] Complex scattering factors were taken from the SHELXL-2016[6] programme running under the Olex2 programme.[7] The three structures were solved with SHELXT[8] and refined by full-matrix least-squares on F^2. All hydrogen atoms were included in calculated positions and refined in riding mode. EXTI correction was employed to complete the refinement of **10a**. Refinement converged with anisotropic displacement parameters for all non-hydrogen atoms. Crystal data and details on data collection and refinement are summarised in **Table S1**.

[5] SABADS: Krause, L.; Herbst-Irmer, R.; Sheldrick, G. M.; Stalke, D. *J. Appl. Cryst.* **2015**, *48*, 3-10.
[6] SHELXL: Sheldrick, G. M. *Acta Cryst.* **2008**, *A64*,112-122.
[7] Olex 2: Dolomanov, O. V.; Bourhis, L. J. Gildea, R. J.; Howard, J. A. K.; Puschmann, H. *J, Appl. Cryst.* **2009**, *42*, 339-341.
[8] SHELXT: Sheldrick, G. M. *Acta Cryst.* **2015**, *A71*, 3-8.

4. ^{1}H, ^{13}C, DEPT-135 and ^{19}F NMR spectra

4.1. Reaction mixtures in the synthesis of benzodiazepinone 6b from (*S*)-α-methylbenzylamine.

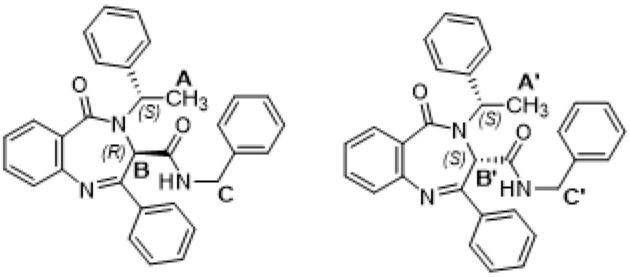

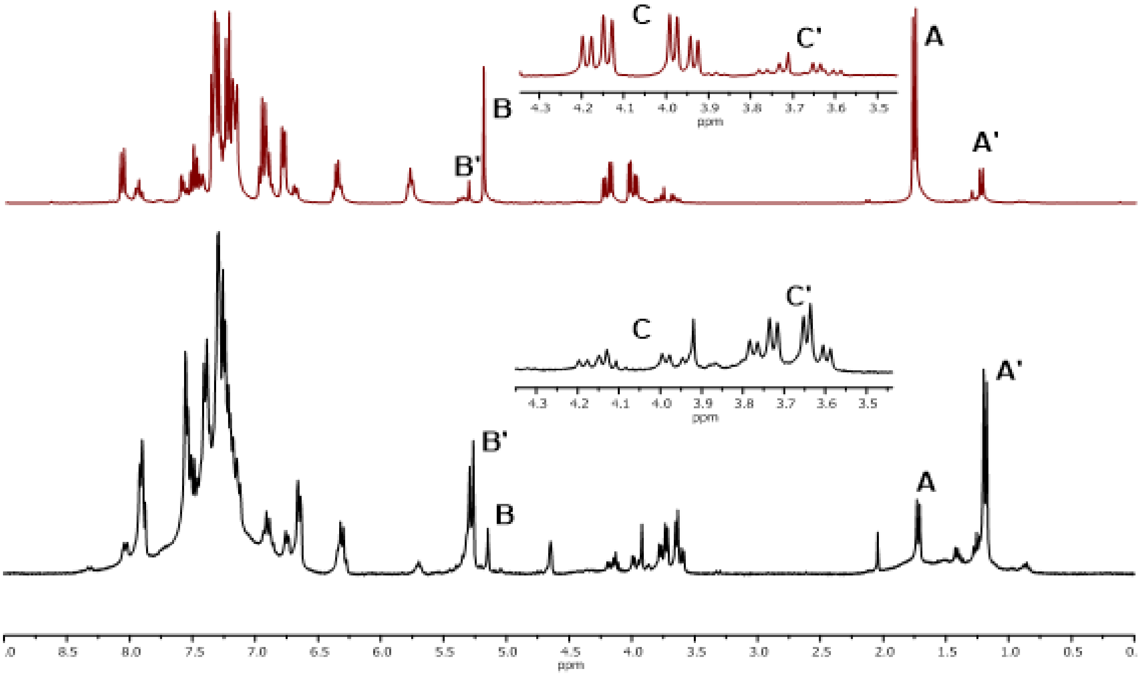

Figure S2. ^{1}H NMR of reaction mixtures in the synthesis of benzodiazepine **6b** from (*S*)-α-methylbenzylamine**.** Results of the two-step Ugi/reduction/cyclization sequence starting from 2-nitrobenzoic acid (top) and results of the one-step synthesis starting from anthranilic acid (down).

4.2. Benzodiazepinones

_N_-Cyclohexyl-4-(2-nitrobenzyl)-5-oxo-2-phenyl-4,5-dihydro-_3H_-benzo[_e_][1,4]diazepine-3-carboxamide. (5b)

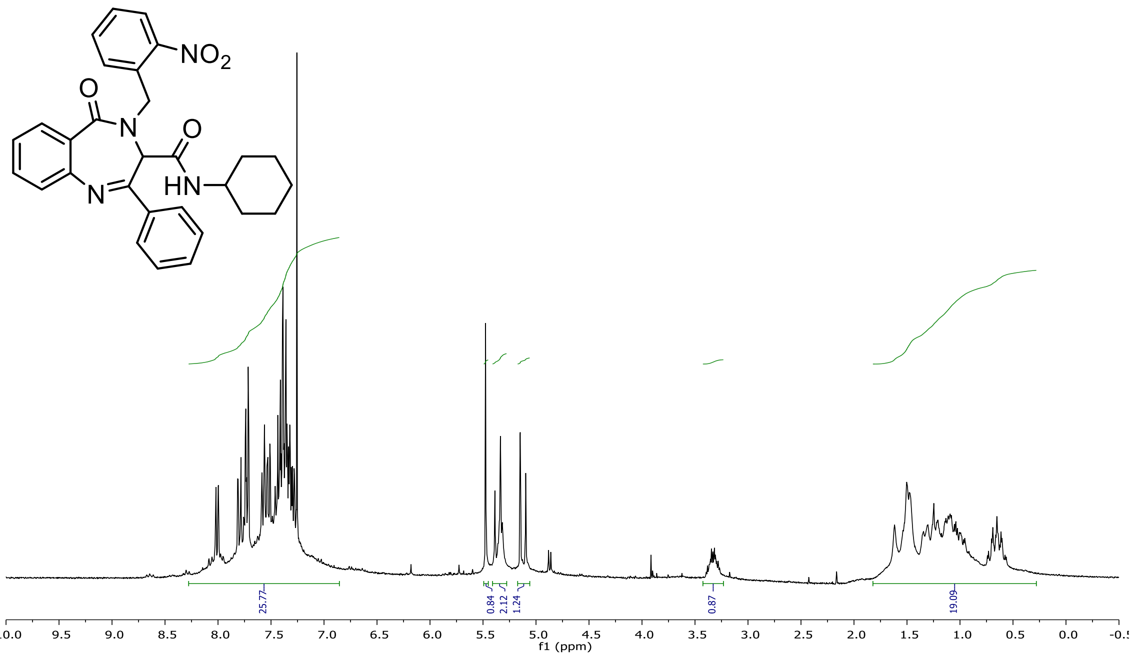

Figure S3. ^{1}H NMR spectrum (300 MHz, $CDCl_3$).

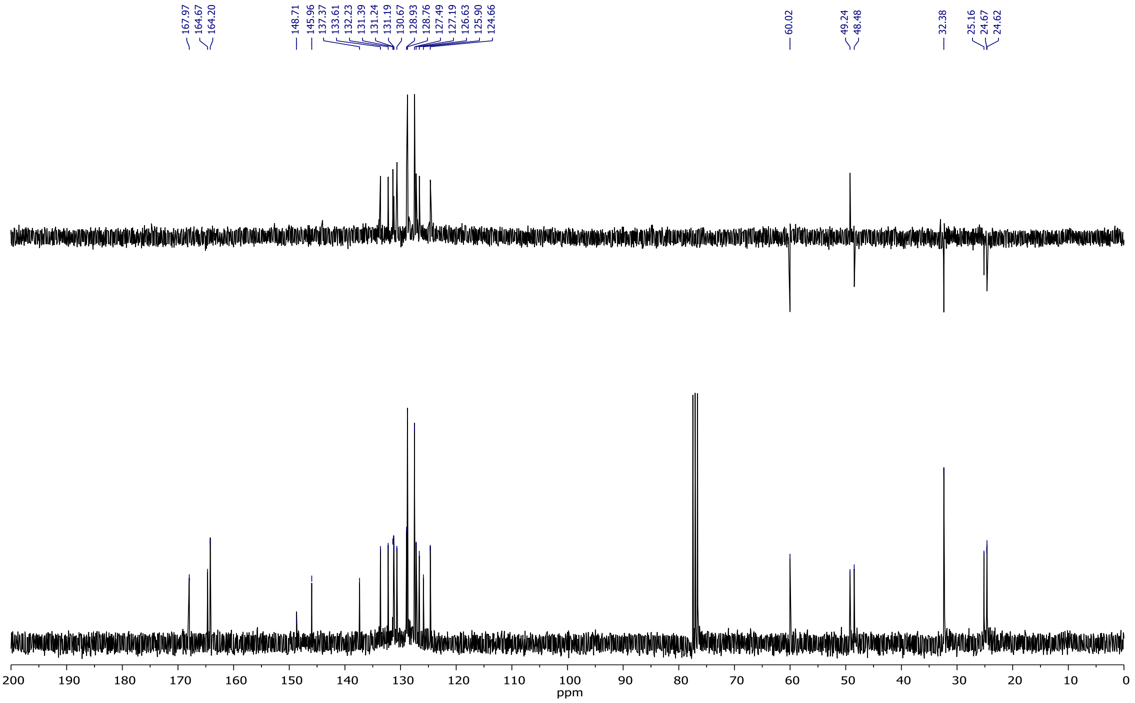

Figure S4. $^{13}C\{^{1}H\}$ and DEPT-135 NMR spectra (75 MHz, $CDCl_3$).

4-(3-Bromopropyl)-*N*-cyclohexyl-5-oxo-2-*p*-tolyl-4,5-dihydro-3*H*-benzo[*e*][1,4]diazepine-3-carboxamide. (5c)

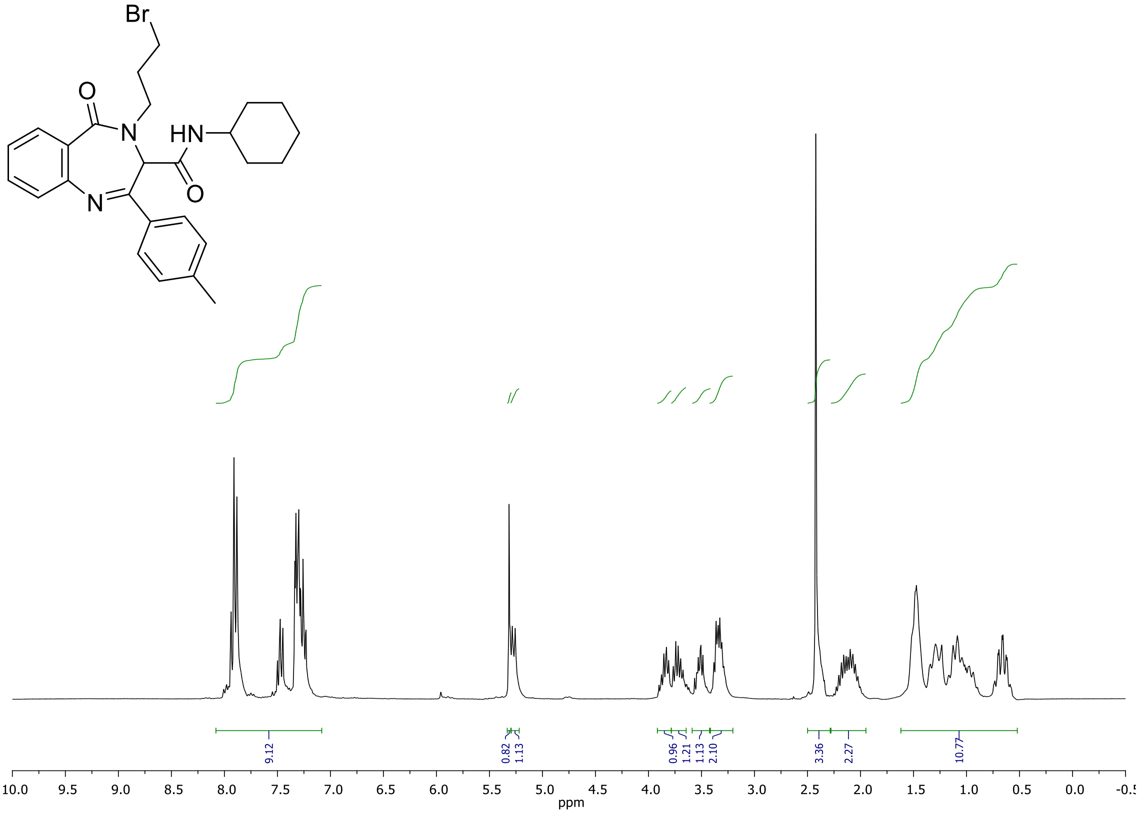

Figure S5. ^{1}H NMR spectrum (300 MHz, $CDCl_3$).

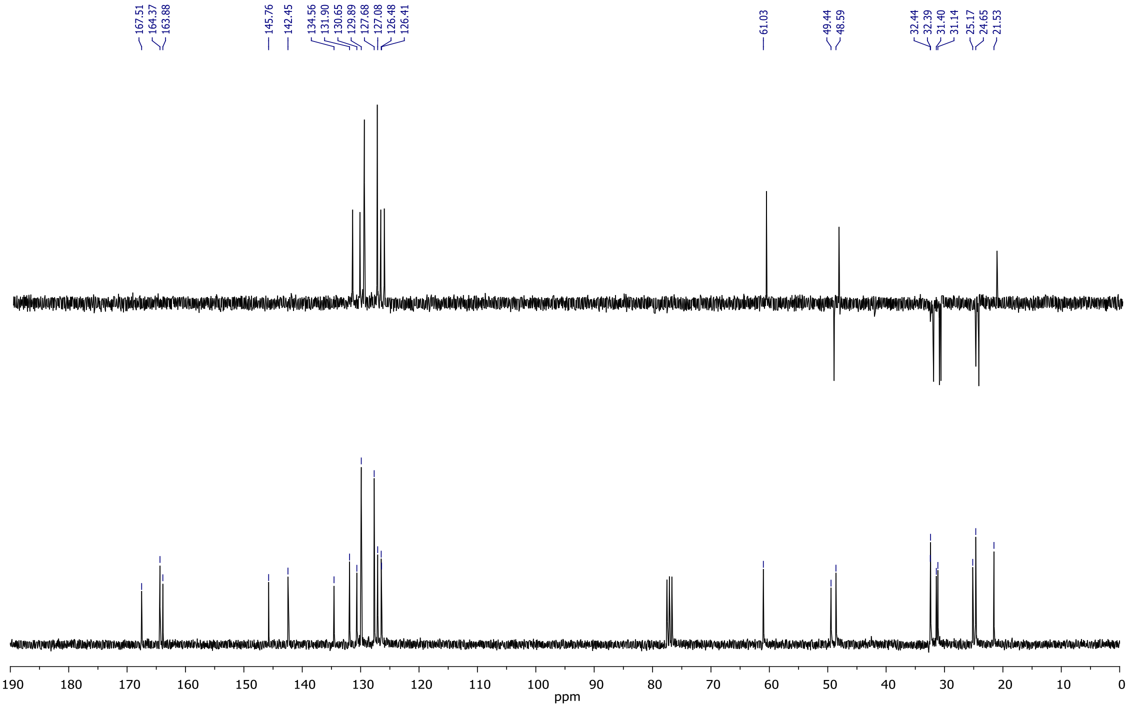

Figure S6. $^{13}C\{^{1}H\}$ and DEPT-135 NMR spectra (75 MHz, $CDCl_3$).

4-Benzyl-*N*-cyclohexyl-8-nitro-5-oxo-2-phenyl-4,5-dihydro-3*H*-benzo[*e*][1,4]diazepine-3-carboxamide. (5e)

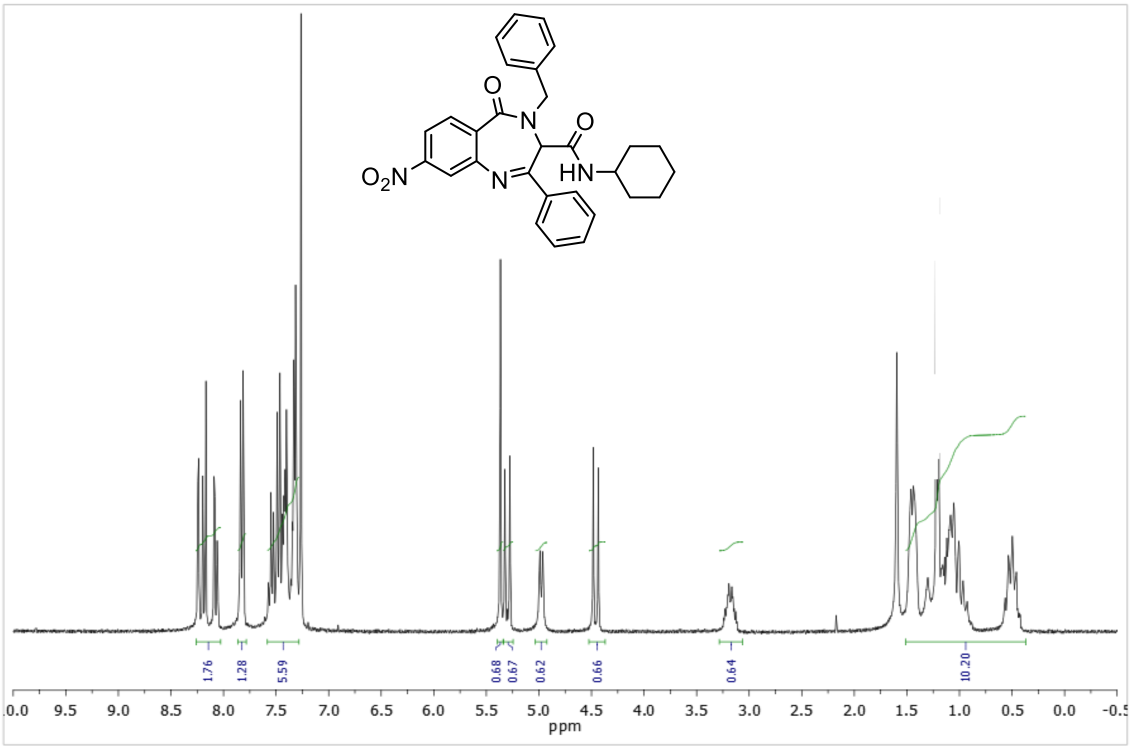

Figure S7. ^{1}H NMR spectrum (300 MHz, $CDCl_3$).

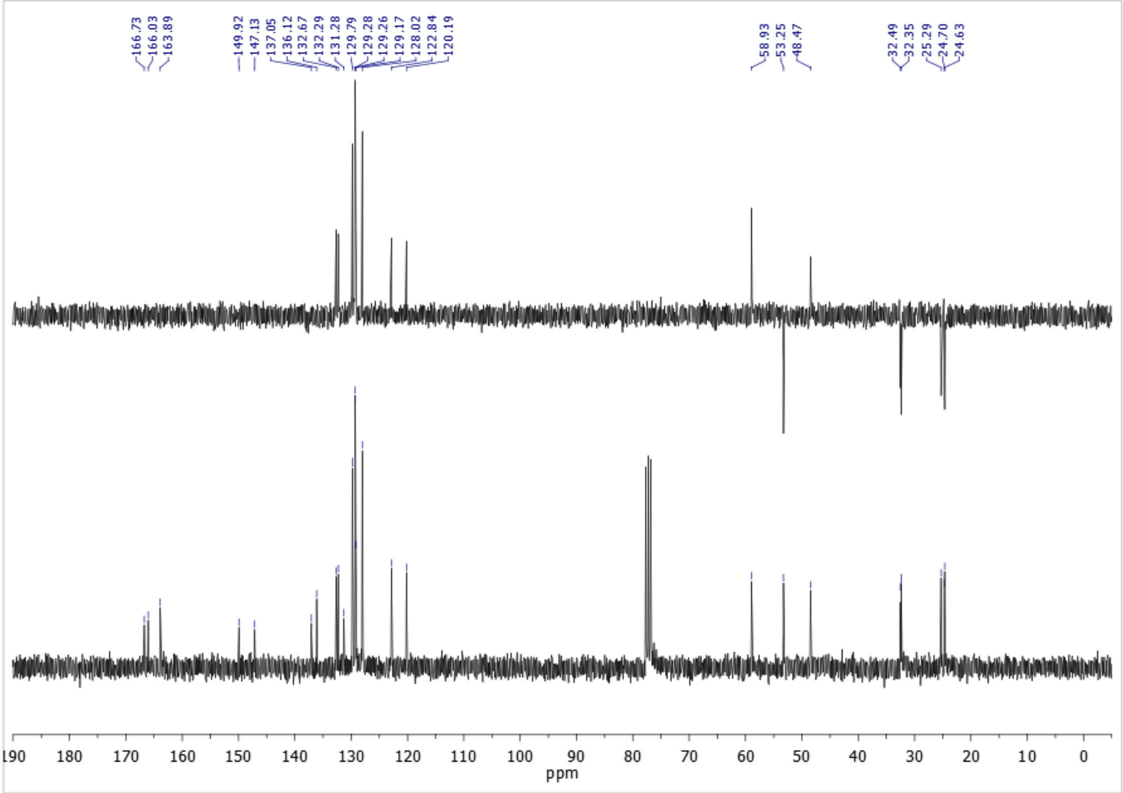

Figure S8. $^{13}C\{^{1}H\}$ and DEPT-135 NMR spectra (75 MHz, $CDCl_3$).

4-Benzyl-*N*-cyclohexyl-7-nitro-5-oxo-2-phenyl-4,5-dihydro-3*H*-benzo[*e*][1,4]diazepine-3-carboxamide. (5f)

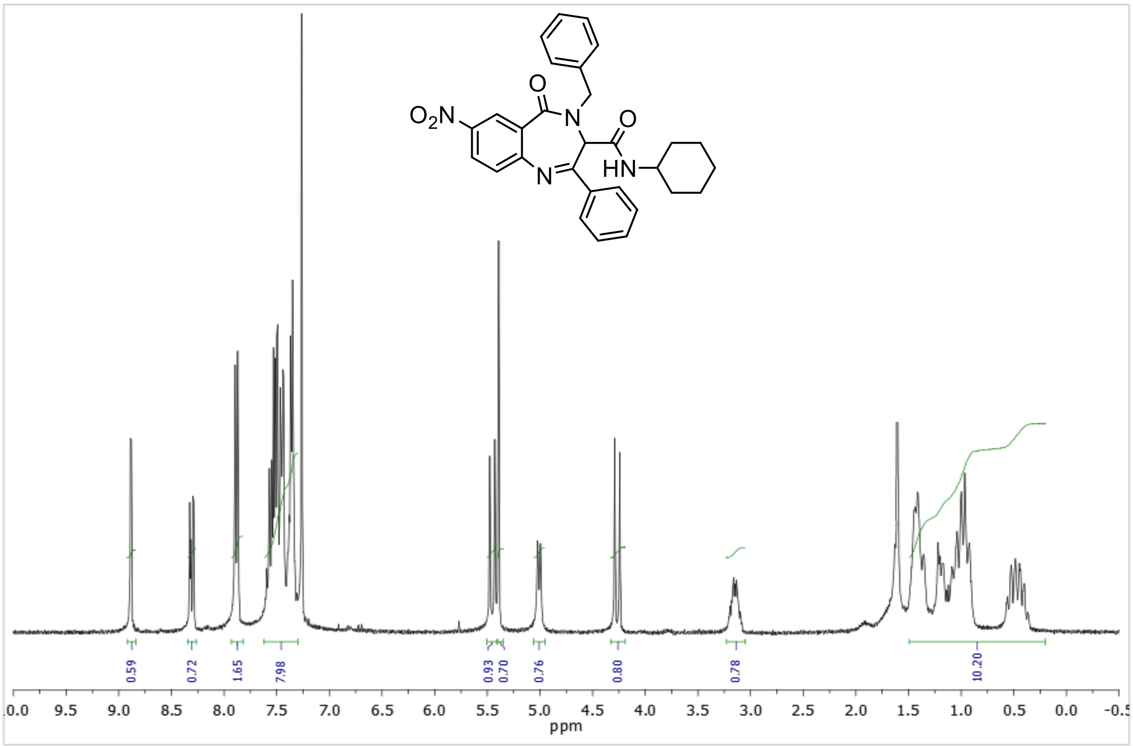

Figure S9. ^{1}H NMR spectrum (300 MHz, $CDCl_3$).

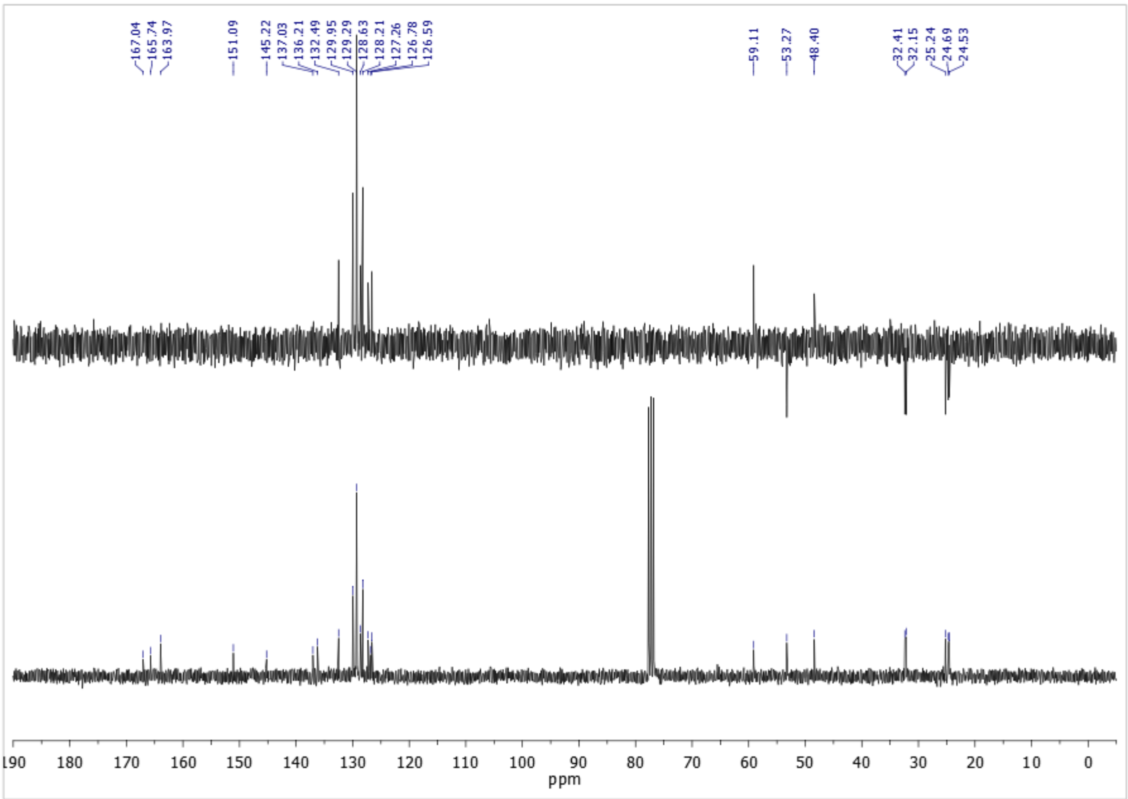

Figure S10. $^{13}C\{^1H\}$ and DEPT-135 NMR spectra (75 MHz, $CDCl_3$).

4-Benzyl-*N*-cyclohexyl-7-iodo-5-oxo-2-phenyl-4,5-dihydro-3*H*-benzo[*e*][1,4]diazepine-3-carboxamide. (5g)

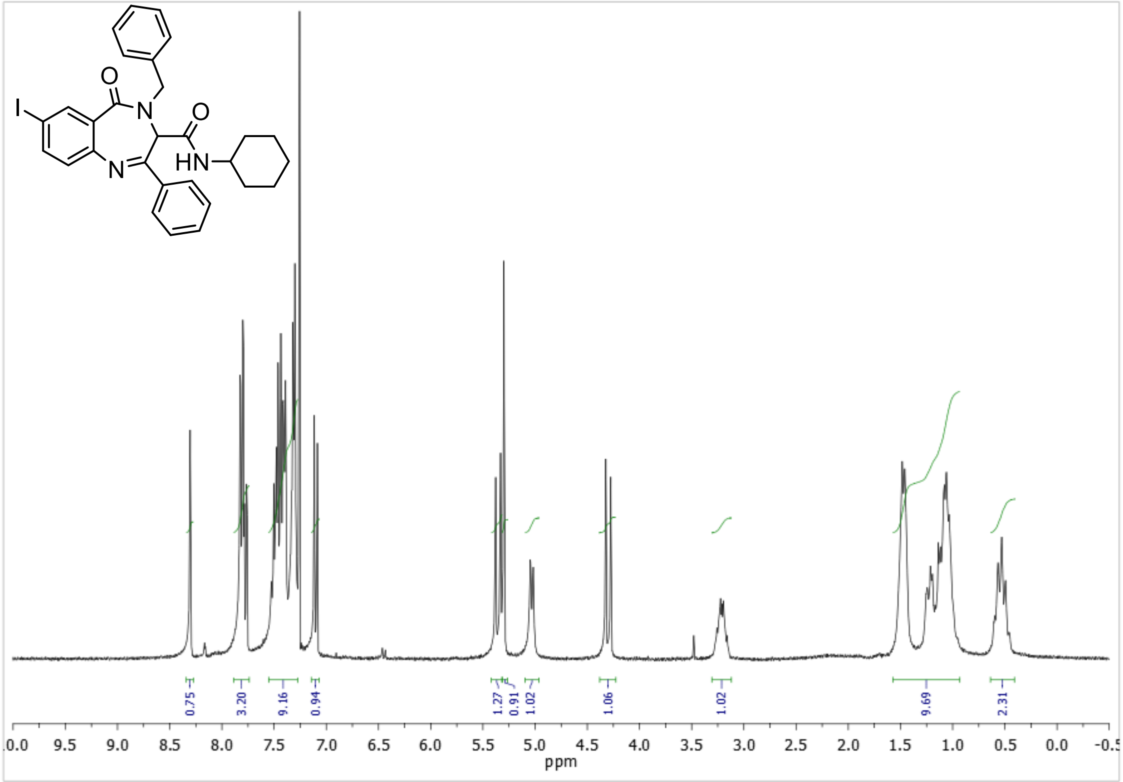

Figure S11. ^{1}H NMR spectrum (300 MHz, $CDCl_3$).

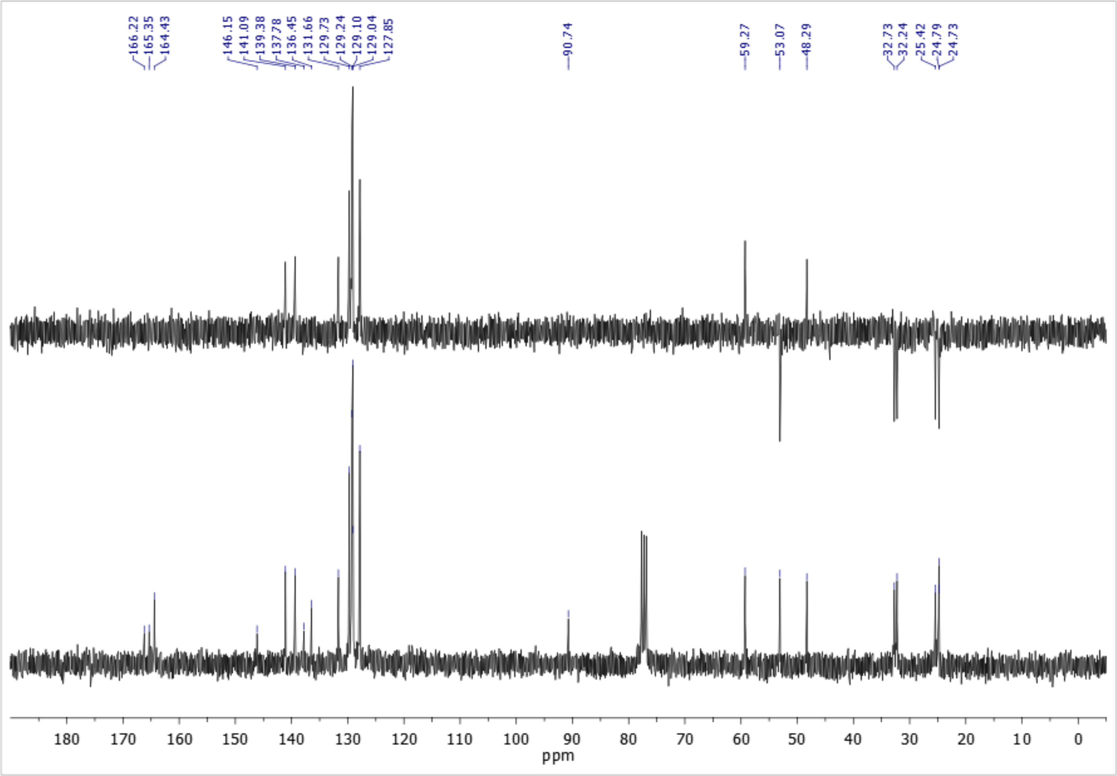

Figure S12. $^{13}C\{^{1}H\}$ and DEPT-135 NMR spectra (75 MHz, $CDCl_3$).

_N_-Cyclohexyl-7-iodo-4-(2-nitrobenzyl)-5-oxo-2-phenyl-4,5-dihydro-3_H_-benzo[_e_][1,4]diazepine-3-carboxamide. (5h)

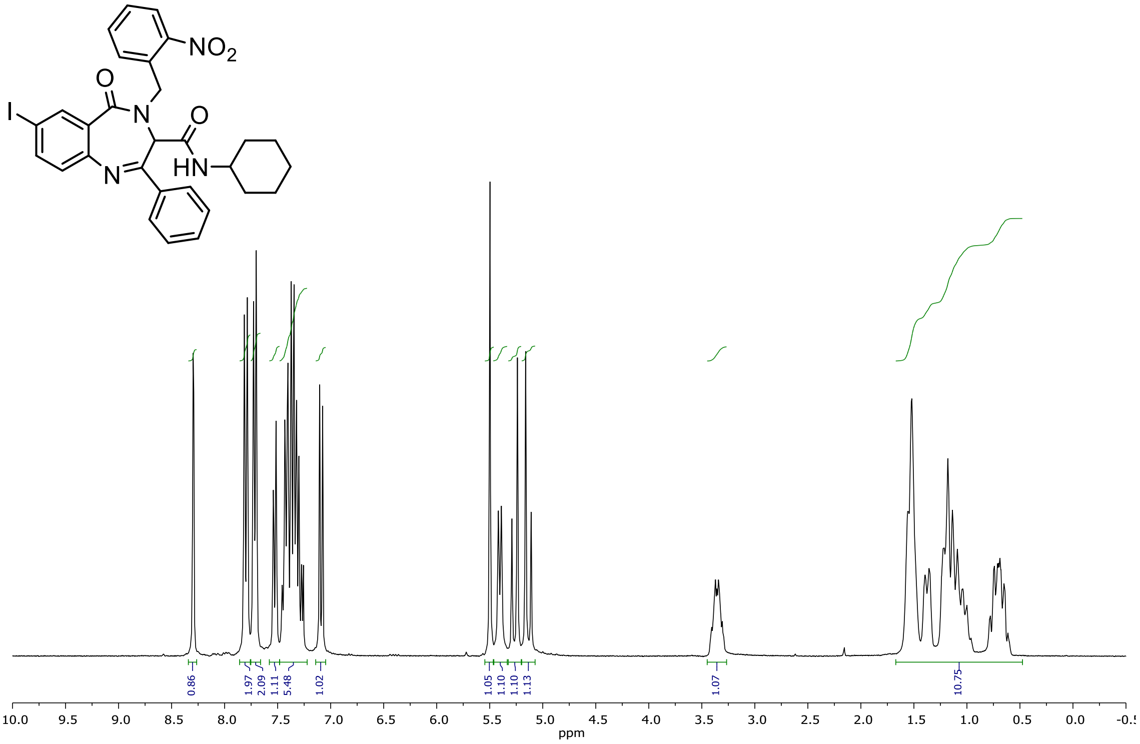

Figure S13. ^{1}H NMR spectrum (300 MHz, $CDCl_3$).

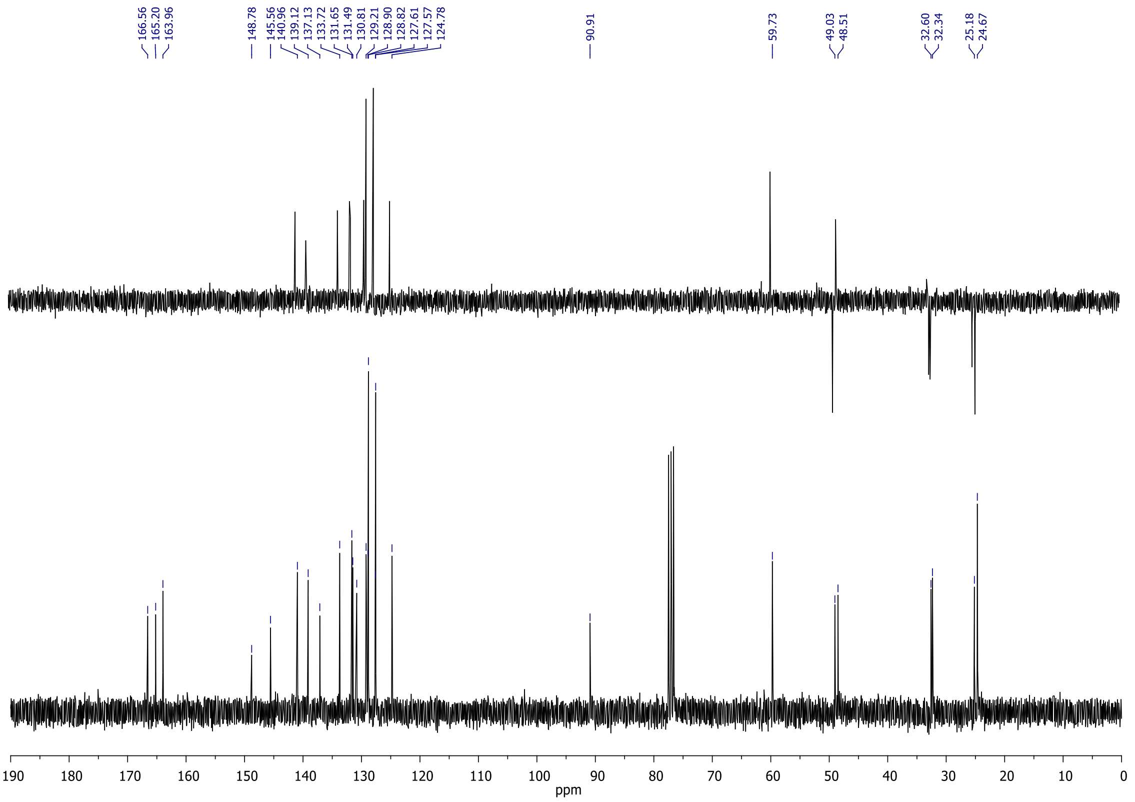

Figure S14. $^{13}C\{^{1}H\}$ and DEPT-135 NMR spectra (75 MHz, $CDCl_3$).

(3*S*)-*N*-Cyclohexyl-2-(4-fluoro)-phenyl-4-((*S*)-methylbenzyl)-5-oxo-4,5-dihydro-3*H*-benzo[*e*][1,4]diazepine-3-carboxamide. (*l*-6c)

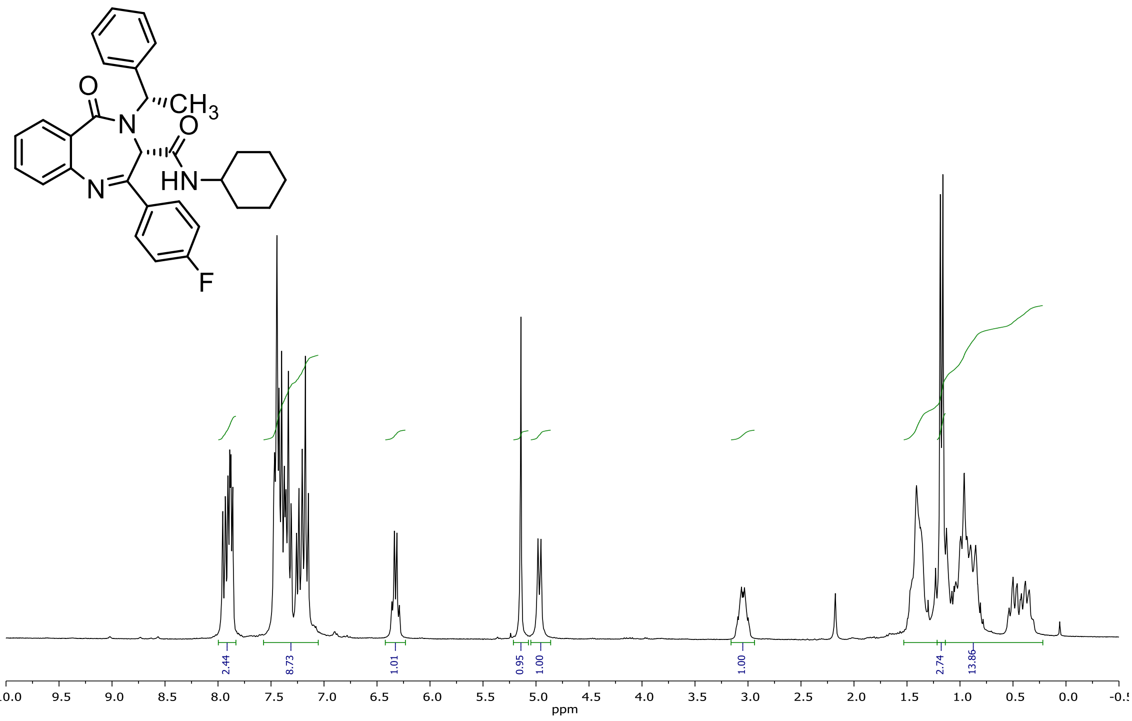

Figure S15. ^{1}H NMR spectrum (300 MHz, $CDCl_3$).

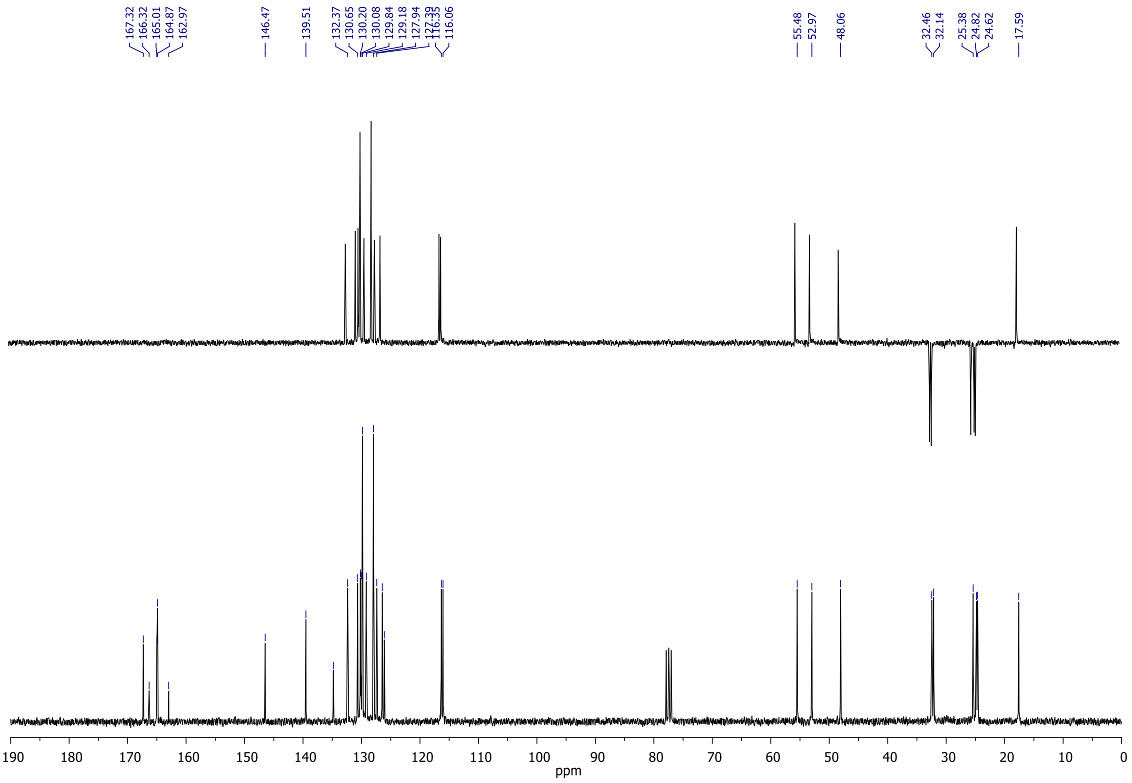

Figure S16. $^{13}C\{^1H\}$ and DEPT-135 NMR spectra (75 MHz, $CDCl_3$).

(6*R,15*S**)-*N*-Cyclohexyl-6-phenyl-5,7,12,14-tetrahydro-6*H*-6,13-methanodibenzo[*d,i*][1,3,7]triazecine-15-carboxamide. (7a)**

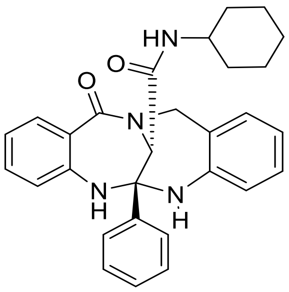

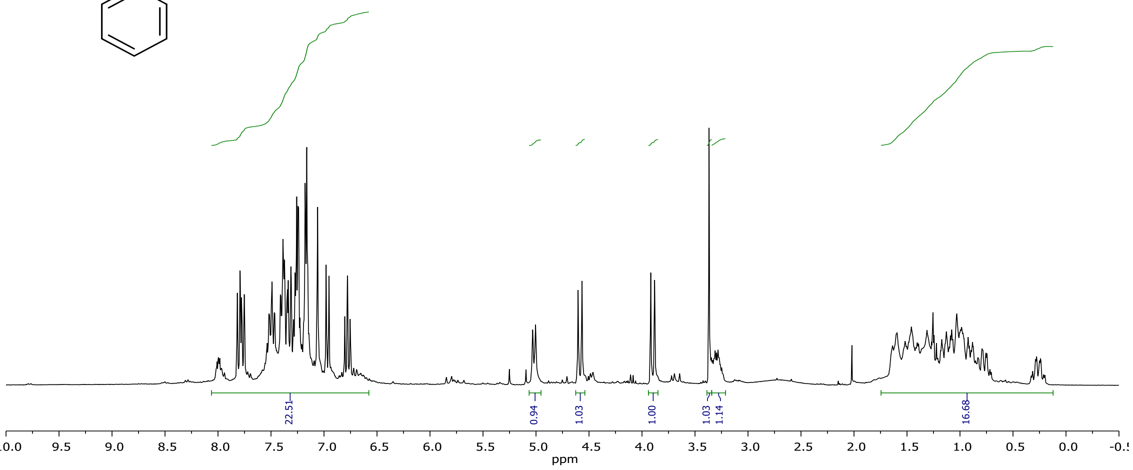

Figure S17. ^{1}H NMR spectrum (300 MHz, $CDCl_3$).

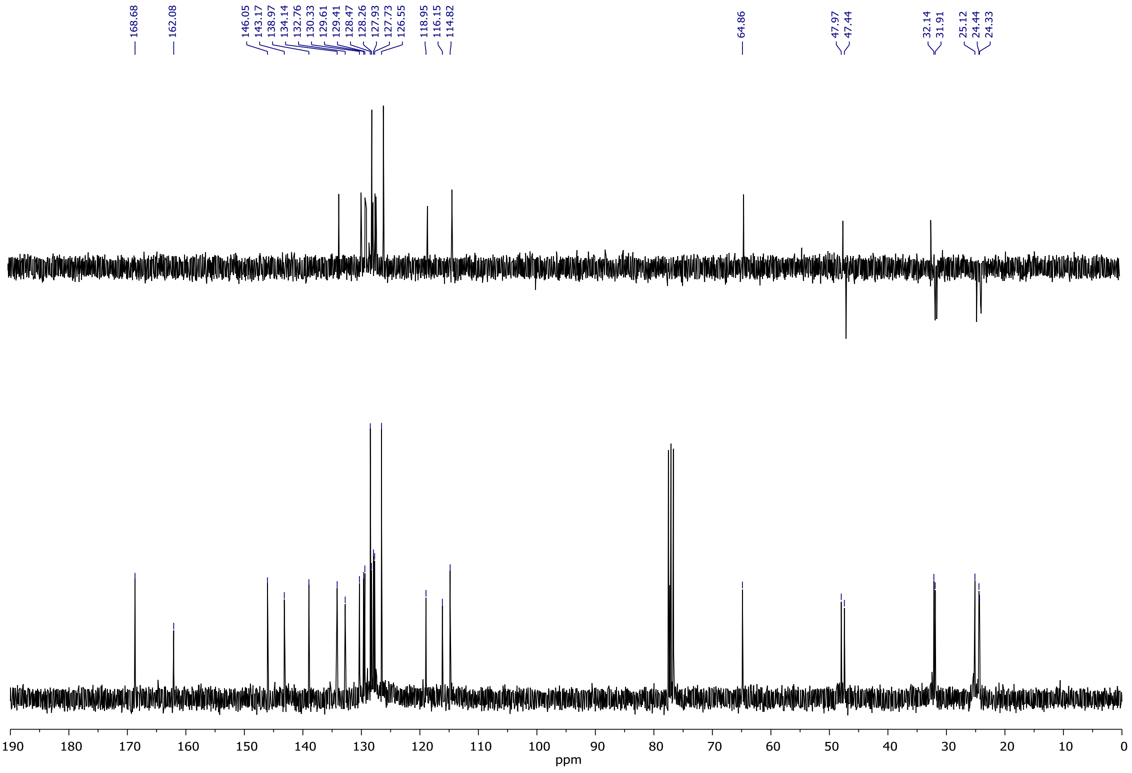

Figure S18. ^{13}C{^{1}H} and DEPT-135 NMR spectra (75 MHz, $CDCl_3$).

(6*R,15*S**)-*N*-Cyclohexyl-6-phenyl-2-iodo-5,7,12,14-tetrahydro-*6H*-6,13-methanodibenzo[*d,i*][1,3,7]triazecine-15-carboxamide. (7b)**

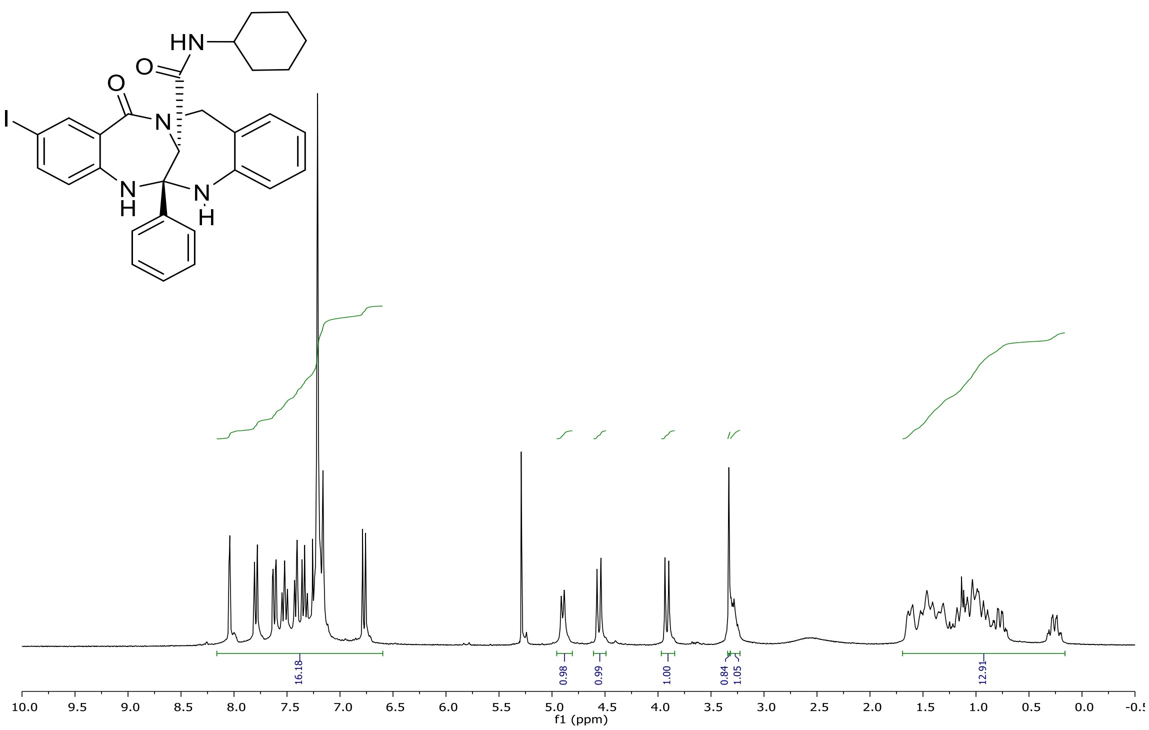

Figure S19. ^{1}H NMR spectrum (300 MHz, $CDCl_3$).

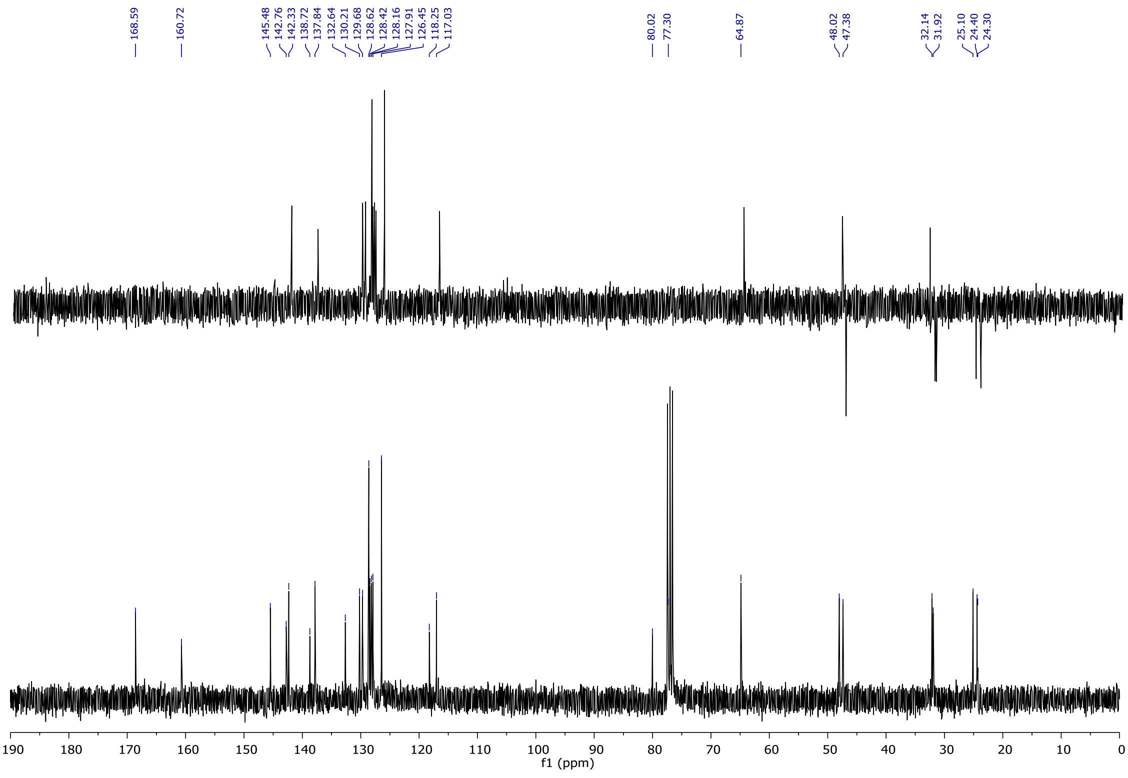

Figure S20. $^{13}C\{^{1}H\}$ and DEPT-135 NMR spectra (75 MHz, $CDCl_3$).

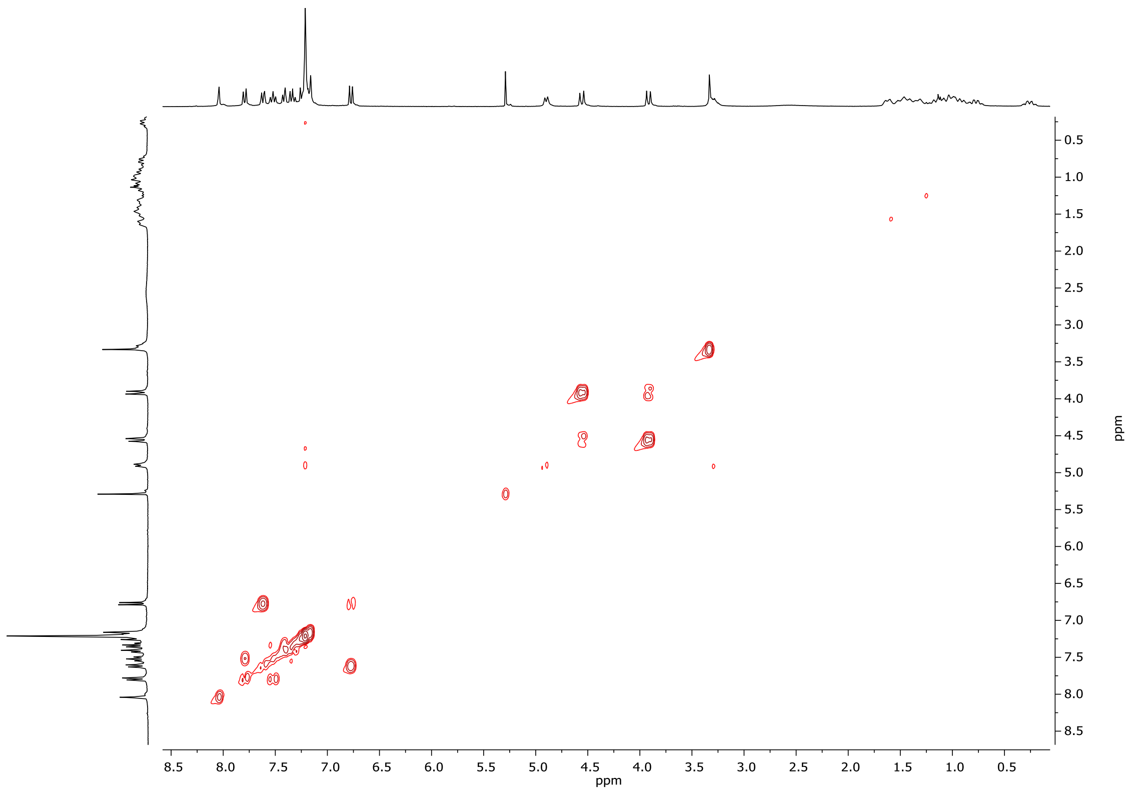

Figure S21. COSY spectrum ($CDCl_3$)

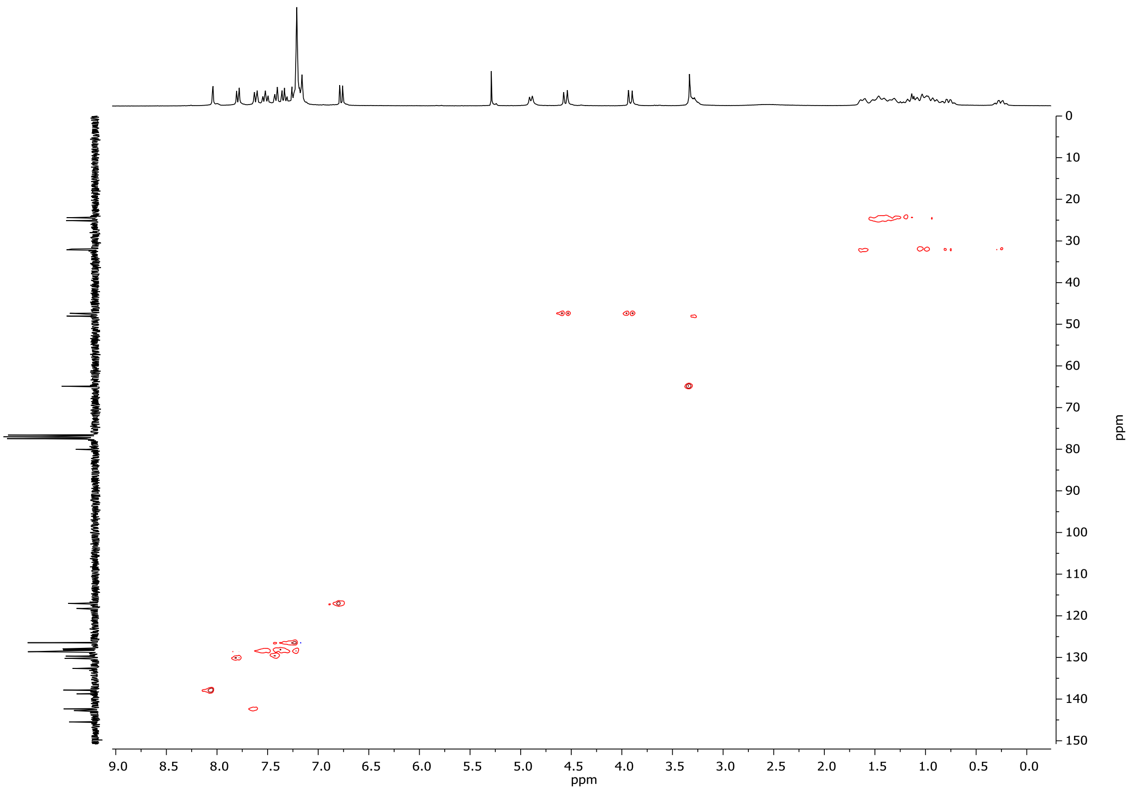

Figure S22. HMQC spectrum ($CDCl_3$)

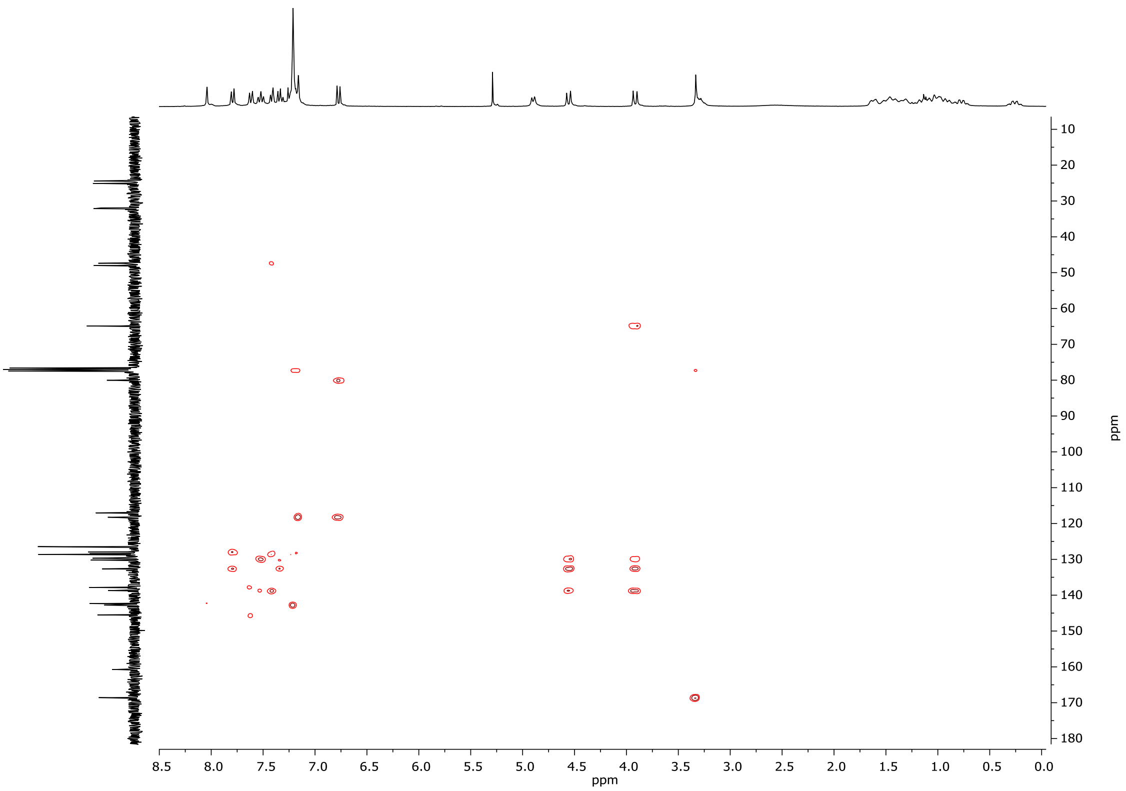

Figure S23. HMBC spectrum ($CDCl_3$)

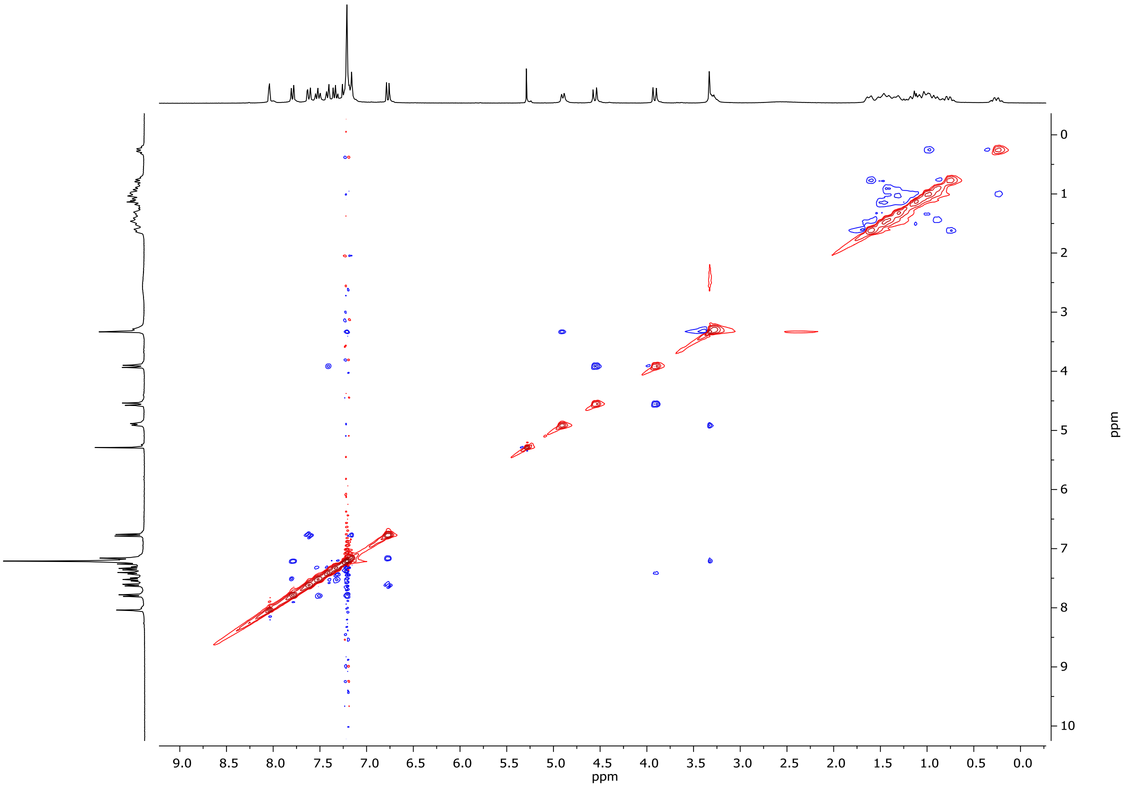

Figure S24. NOESY spectrum ($CDCl_3$)

4.3. Piperazinones

(3*R,4*R**)-2-Benzyl-*N*-(*tert*-butyl)-4-hydroxy-1-oxo-4-phenyl-1,2,3,4-tetrahydropyrrolo[1,2-*a*]pyrazine-3-carboxamide. (9a)**

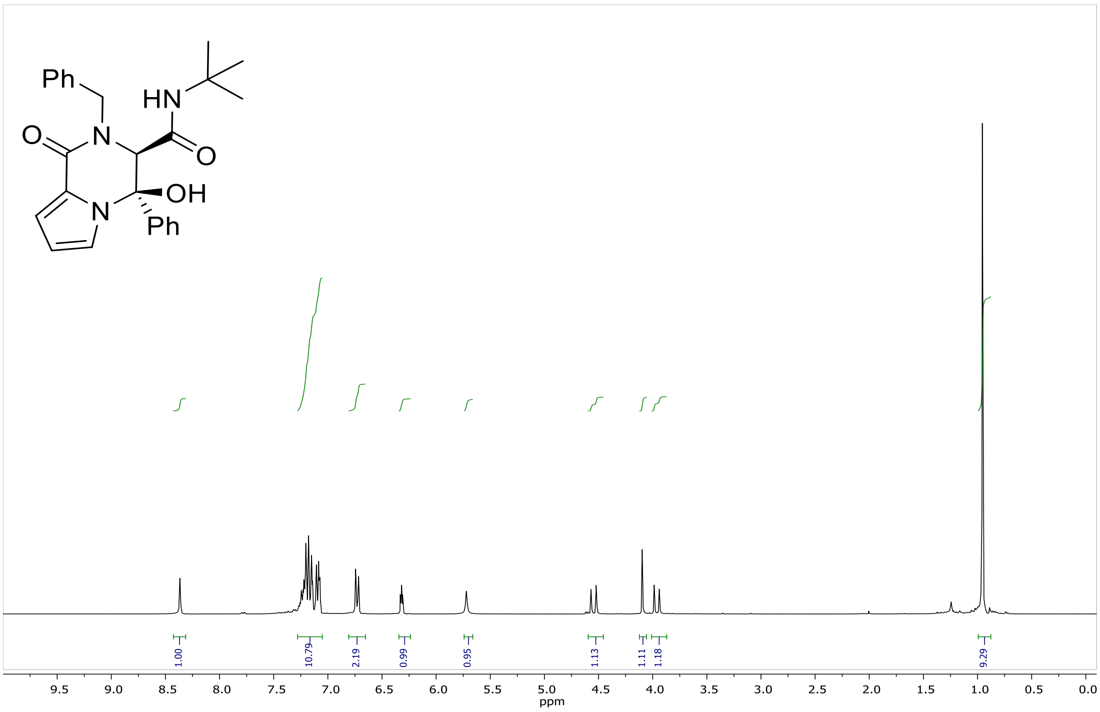

Figure S25. ^{1}H NMR spectrum (300 MHz, $CDCl_3$).

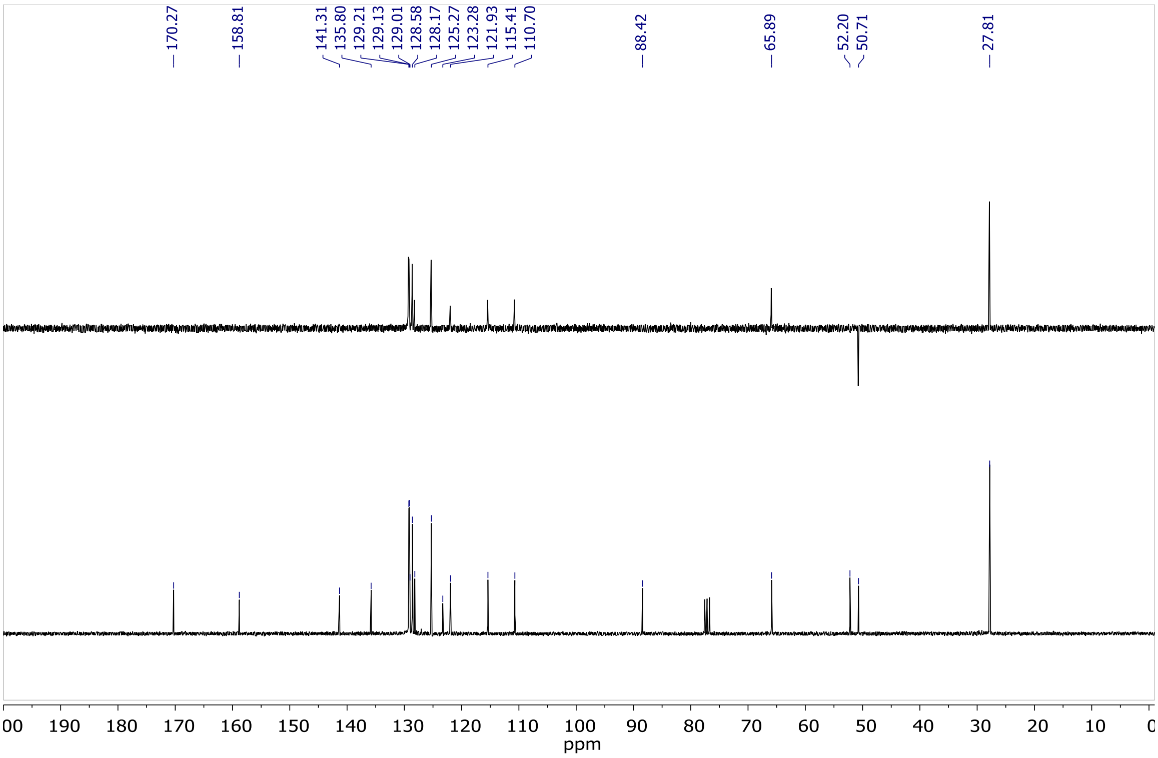

Figure S26. $^{13}C\{^{1}H\}$ and DEPT-135 NMR spectra (75 MHz, $CDCl_3$).

(3*R,4*R**)-2-(*tert*-Butyl)-*N*-cyclohexyl-4-hydroxy-1-oxo-4-phenyl-1,2,3,4-tetrahydropyrrolo[1,2-*a*]pyrazine-3-carboxamide. (9b)**

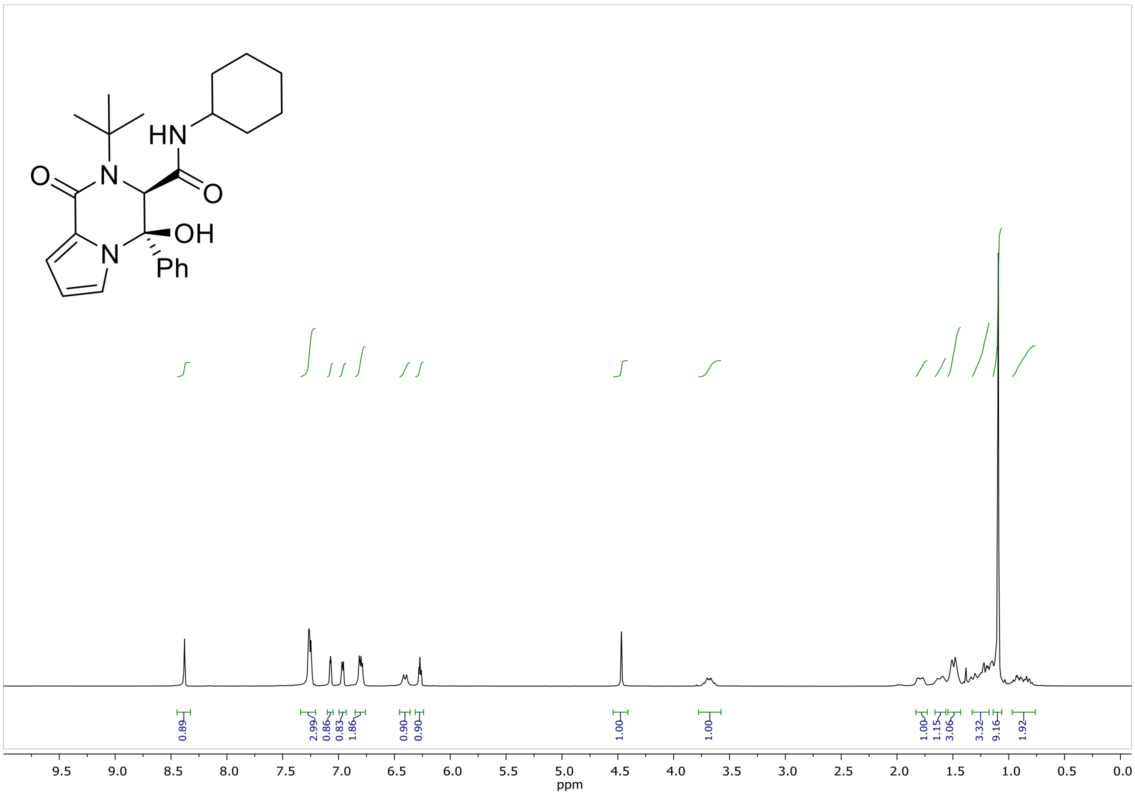

Figure S27. ^{1}H NMR spectrum (300 MHz, $CDCl_3$).

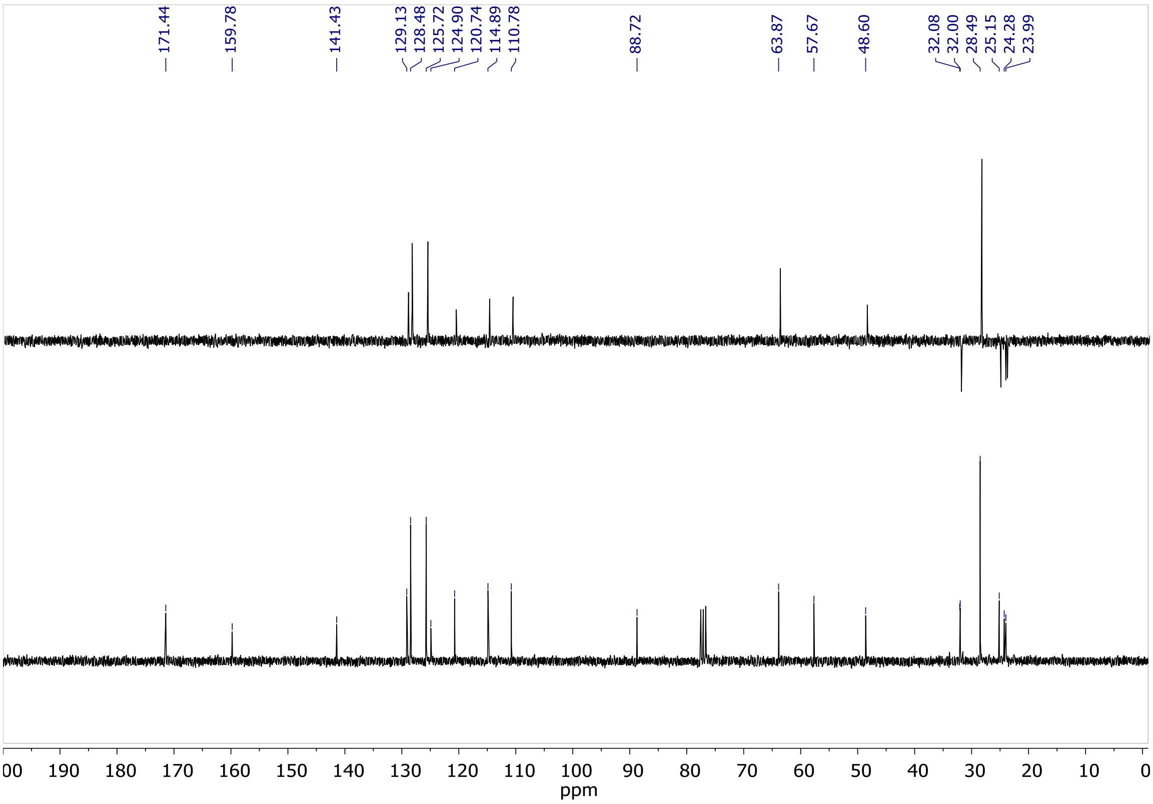

Figure S28. $^{13}C\{^{1}H\}$ and DEPT-135 NMR spectra (75 MHz, $CDCl_3$).

(3*R,4*R**)-2-(3-Bromopropyl)-*N*-cyclohexyl-4-hydroxy-1-oxo-4-phenyl-1,2,3,4-tetrahydropyrrolo[1,2-*a*]pyrazine-3-carboxamide. (9c)**

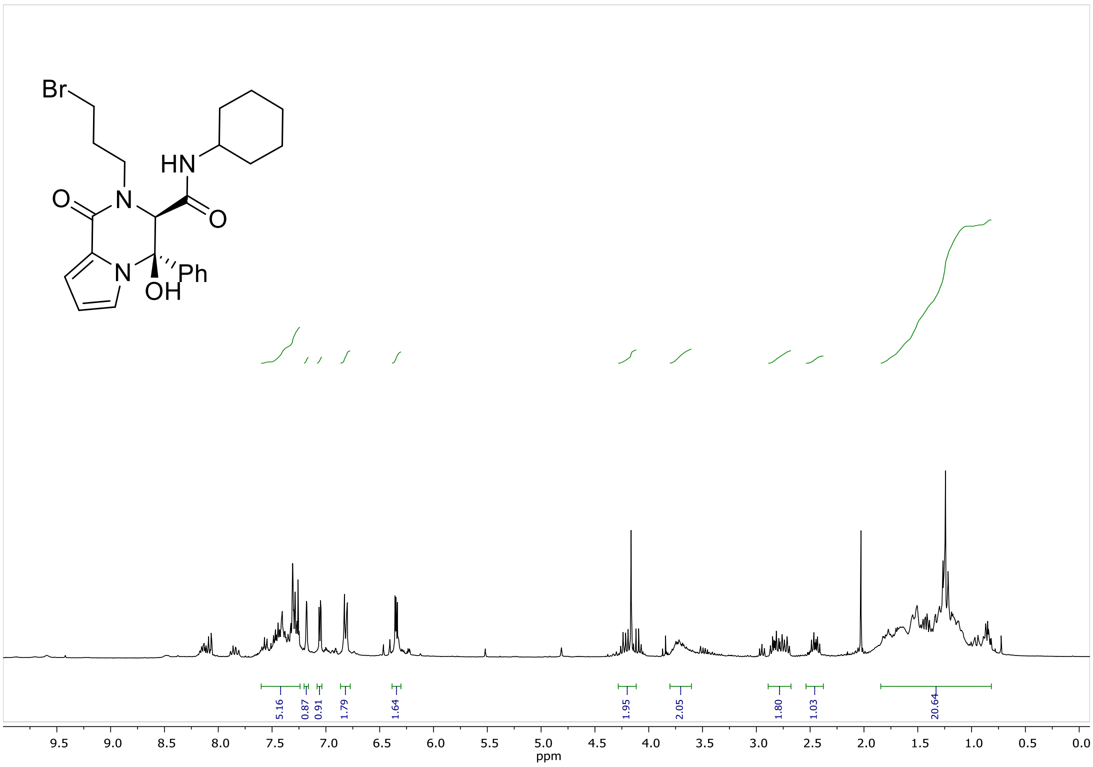

Figure S29. ^{1}H NMR spectrum (300 MHz, $CDCl_3$).

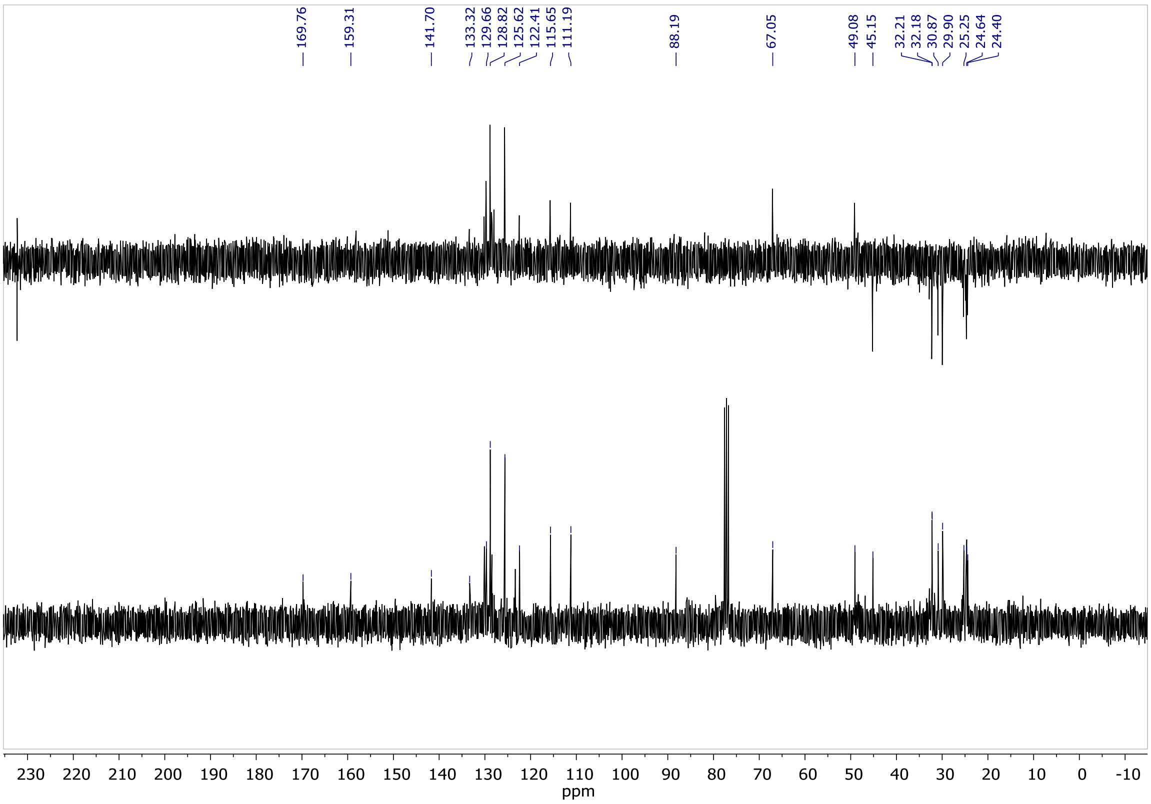

Figure S30. $^{13}C\{^{1}H\}$ and DEPT-135 NMR spectra (75 MHz, $CDCl_3$).

(3*R,4*R**)-2-(3-Bromopropyl)-*N*-(*tert*-butyl)-4-hydroxy-1-oxo-4-phenyl-1,2,3,4-tetrahydropyrrolo[1,2-*a*]pyrazine-3-carboxamide. (9d)**

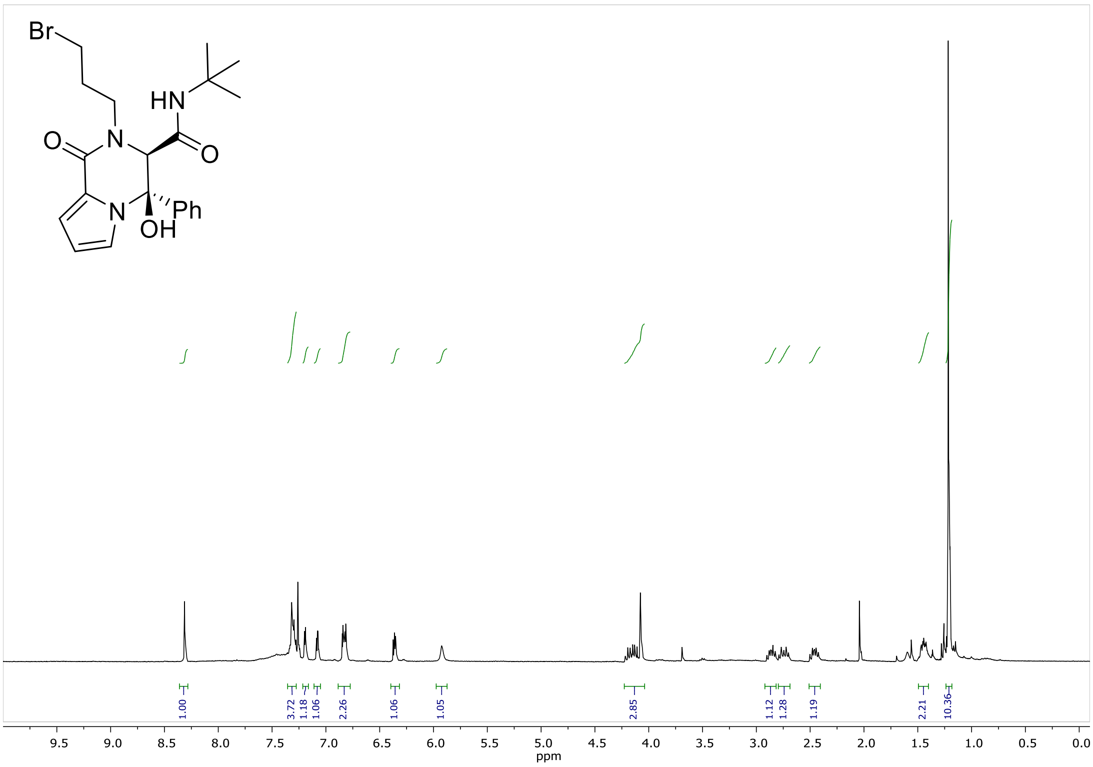

Figure S31. ^{1}H NMR spectrum (300 MHz, $CDCl_3$).

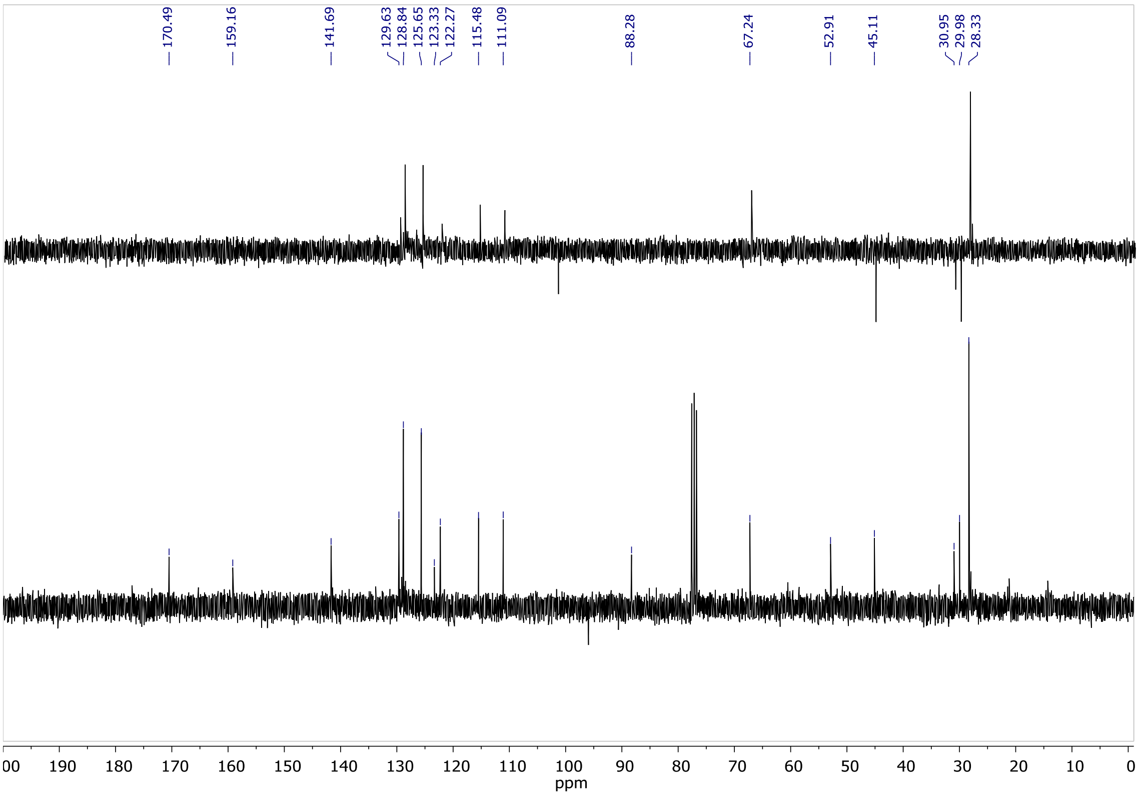

Figure S32. $^{13}C\{^{1}H\}$ and DEPT-135 NMR spectra (75 MHz, $CDCl_3$).

(3*R,4*R**)-2-(3-Bromopropyl)-*N*-(*tert*-butyl)-4-(4-fluorophenyl)-4-hydroxy-1-oxo-1,2,3,4-tetrahydropyrrolo[1,2-*a*]pyrazine-3-carboxamide. (9e)**

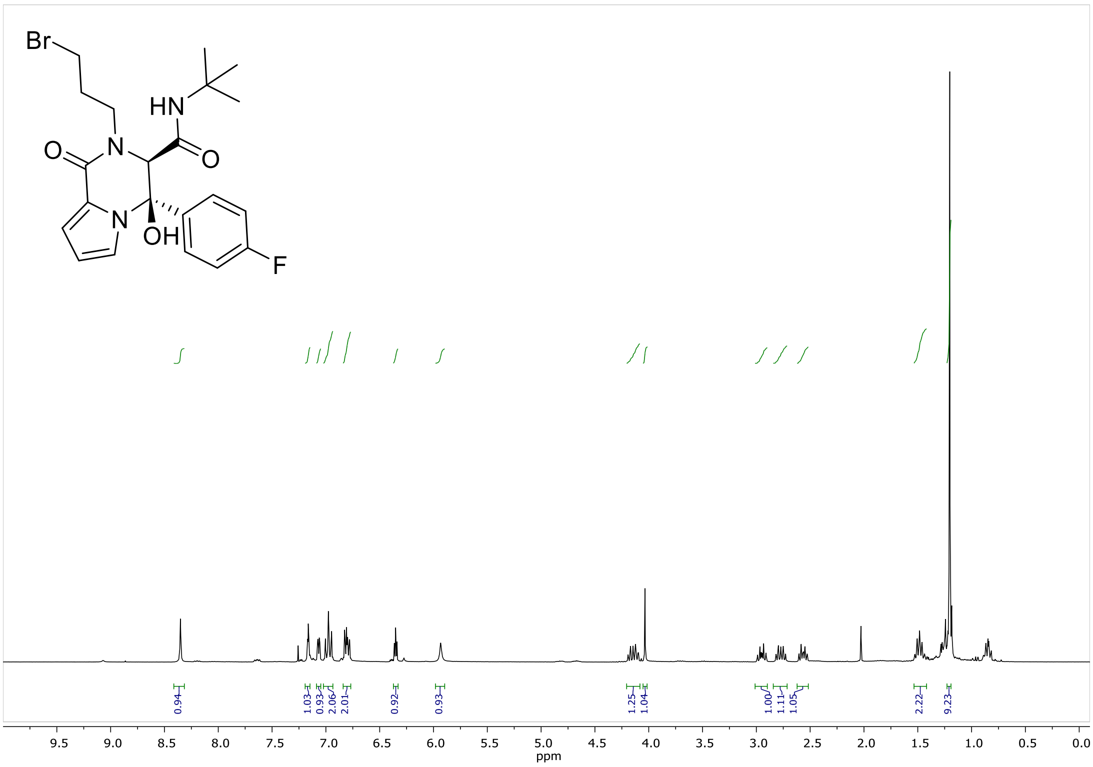

Figure S33. ^{1}H NMR spectrum (300 MHz, $CDCl_3$).

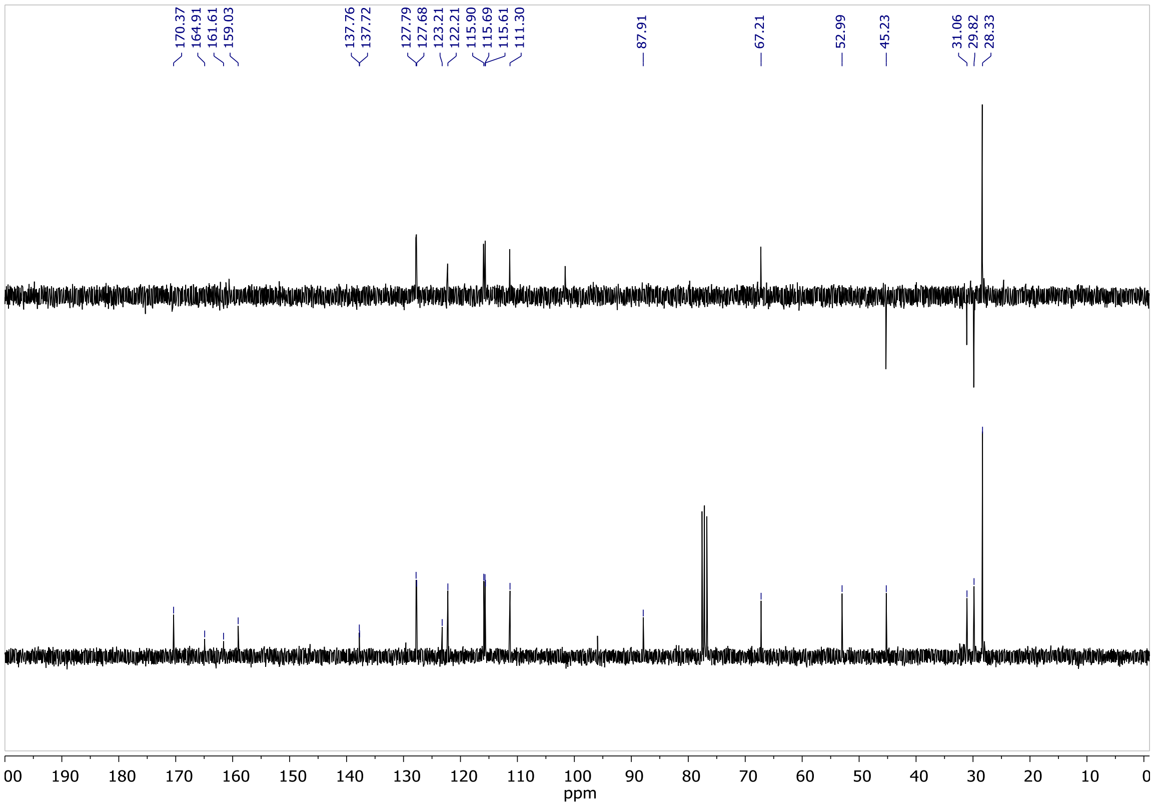

Figure S34. $^{13}C\{^{1}H\}$ and DEPT-135 NMR spectra (75 MHz, $CDCl_3$).

(3*R,4*R**)-*N*-Cyclohexyl-4-hydroxy-2-(2-nitrobenzyl)-1-oxo-4-phenyl-1,2,3,4-tetrahydropyrrolo[1,2-*a*]pyrazine-3-carboxamide. (9f)**

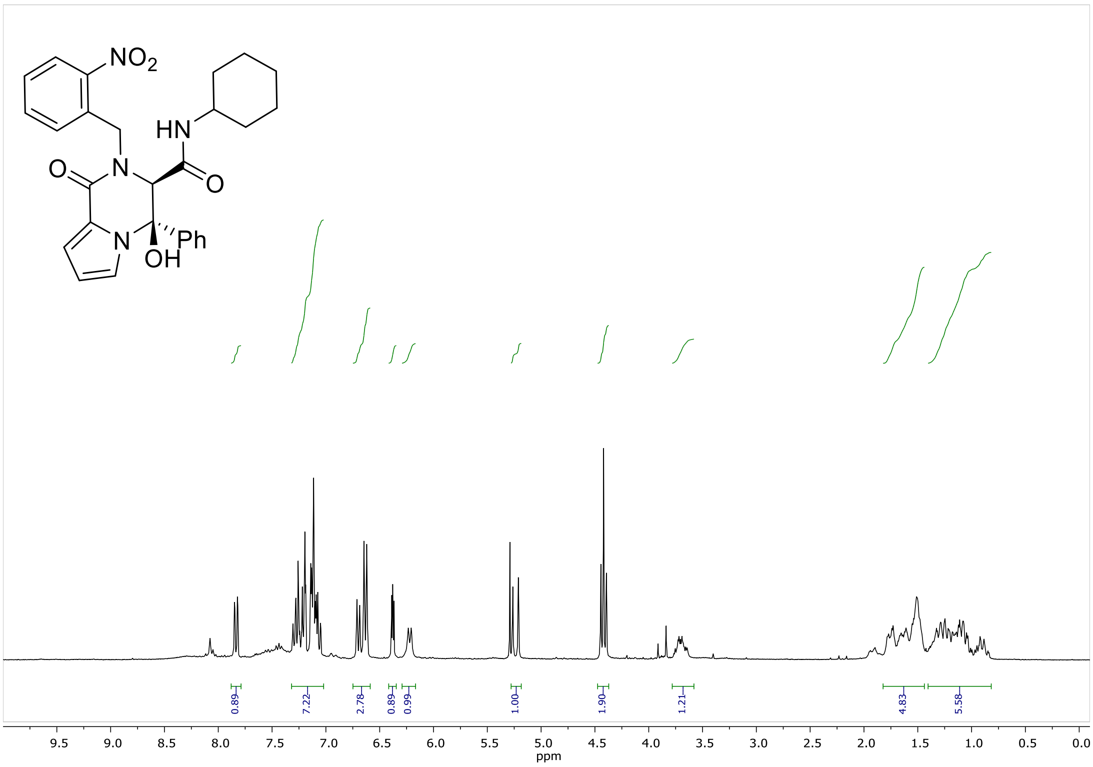

Figure S35. ^{1}H NMR spectrum (300 MHz, $CDCl_3$).

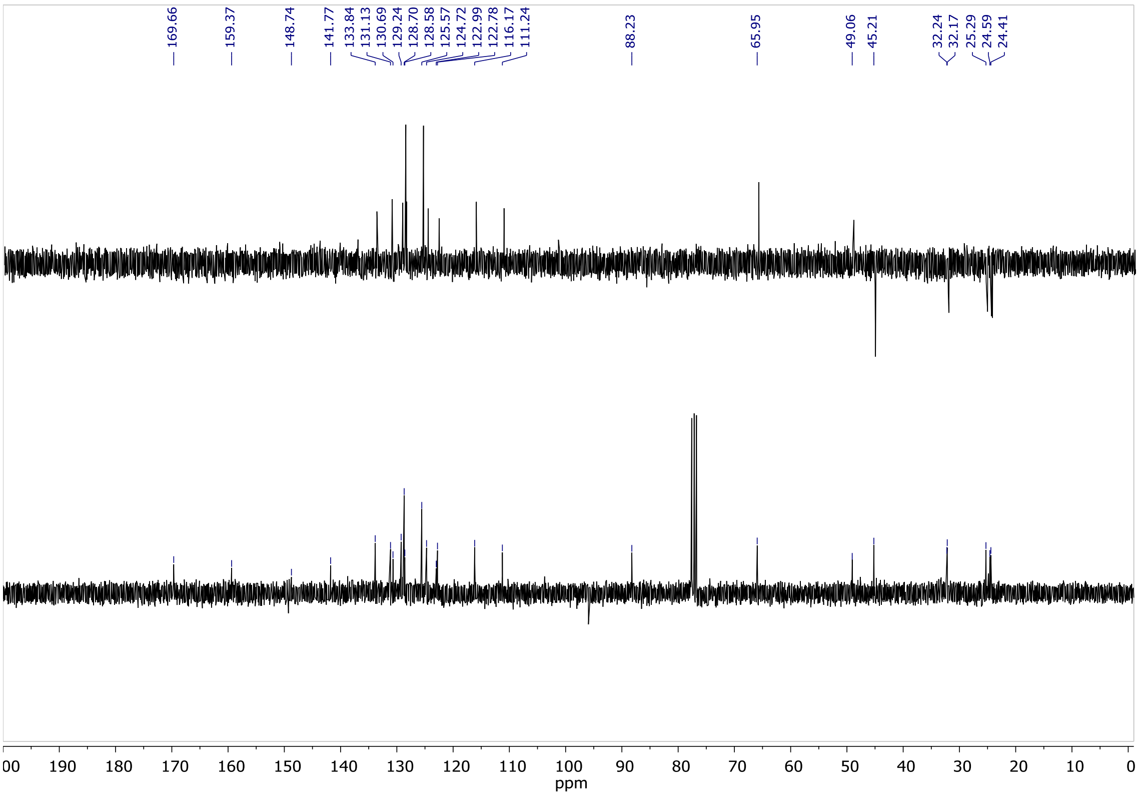

Figure S36. $^{13}C\{^{1}H\}$ and DEPT-135 NMR spectra (75 MHz, $CDCl_3$).

(3*R,4*R**)-*N*-(*tert*-Butyl)-4-hydroxy-2-(2-nitrobenzyl)-1-oxo-4-(4-(trifluoromethyl)phenyl)-1,2,3,4-tetrahydropyrrolo[1,2-*a*]pyrazine-3-carboxamide. (9g)**

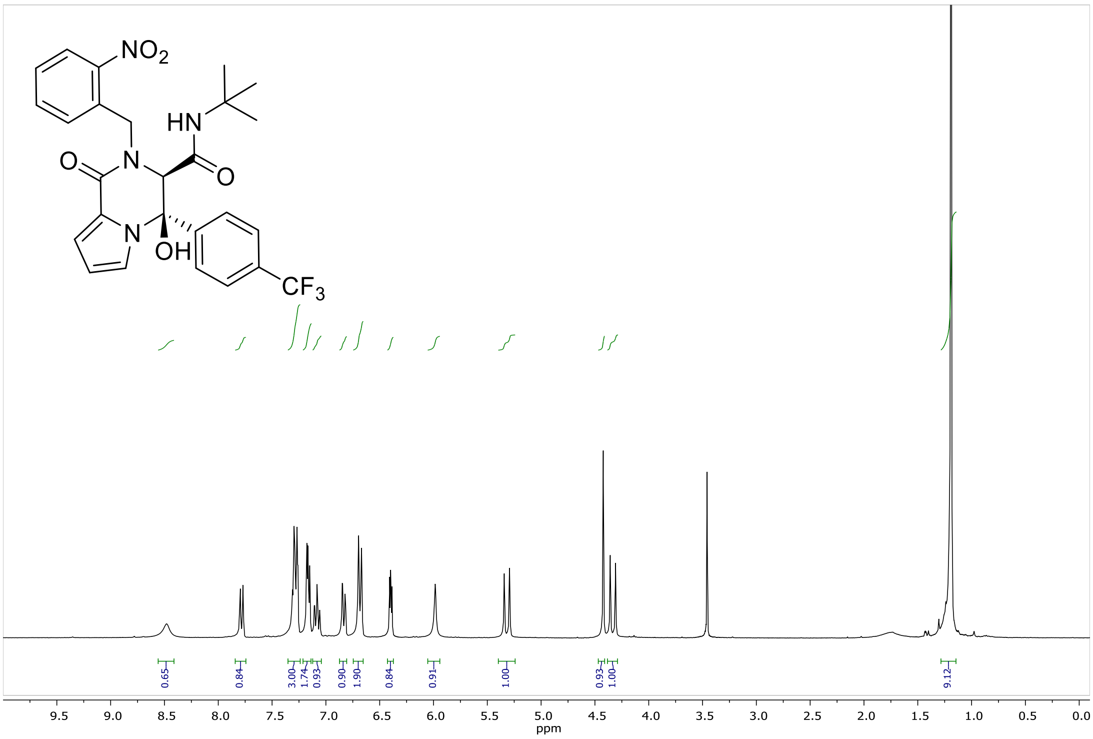

Figure S37. ^{1}H NMR spectrum (300 MHz, $CDCl_3$).

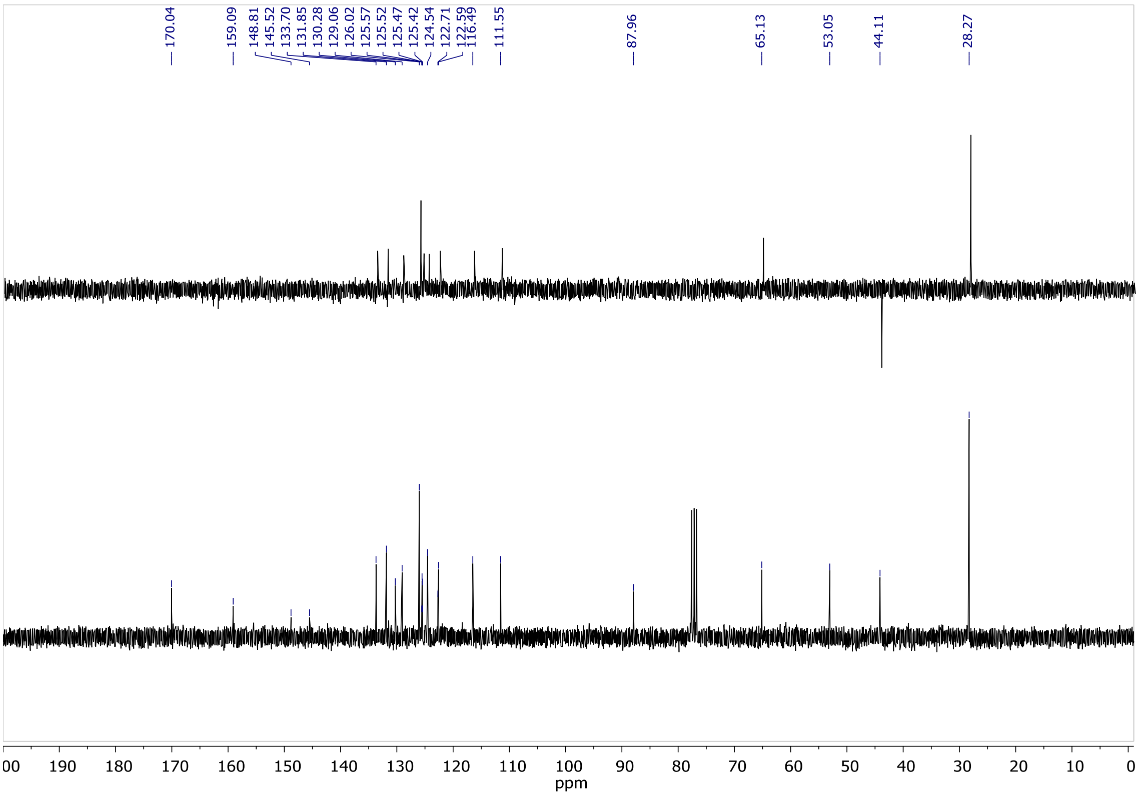

Figure S38. $^{13}C\{^{1}H\}$ and DEPT-135 NMR spectra (75 MHz, $CDCl_3$).

(*3R*,4R)-2-Benzyl-*N*-cyclohexyl-4-hydroxy-1-oxo-4-phenyl-1,2,3,4-tetrahydropyrazino[1,2-*a*]indole-3-carboxamide. (9h)**

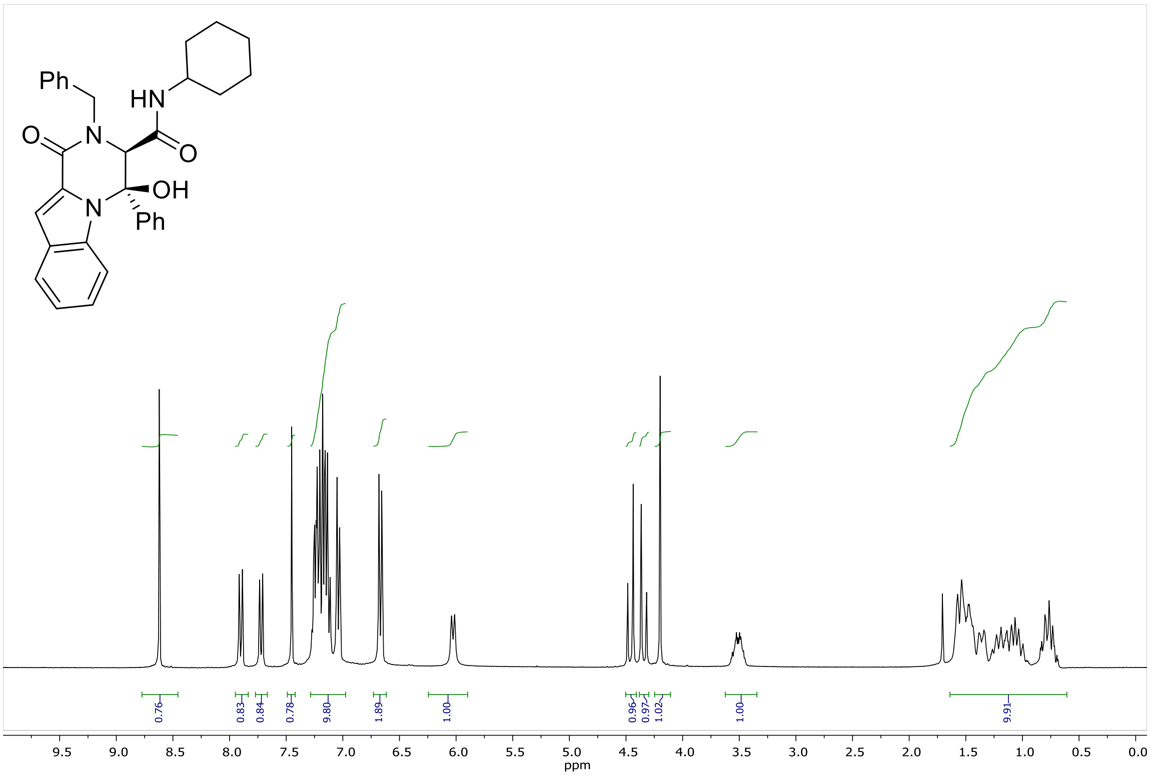

Figure S39. ^{1}H NMR spectrum (300 MHz, $CDCl_3$)

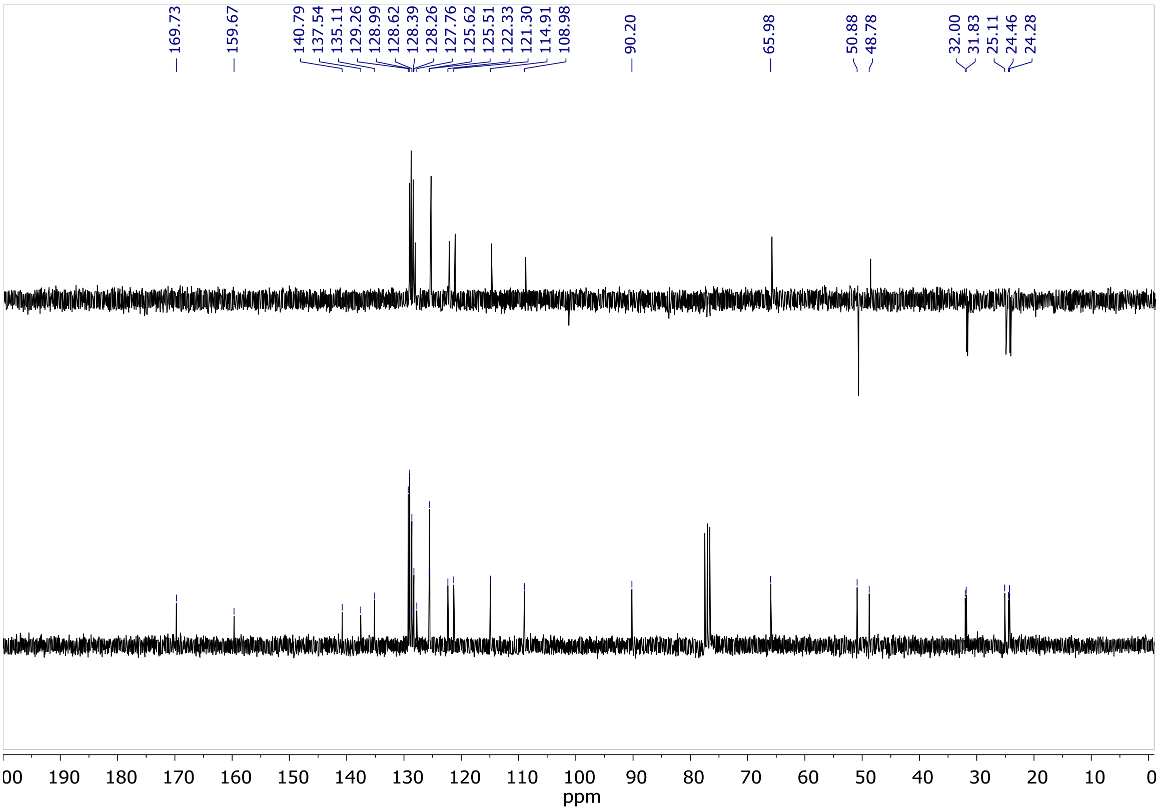

Figure S40. $^{13}C\{^{1}H\}$ and DEPT-135 NMR spectra (75 MHz, $CDCl_3$)

(*3R,*4R**)-*N*-Cyclohexyl-4-(4-fluorophenyl)-4-hydroxy-1-oxo-1,2,3,4-tetrahydropyrazino[1,2-*a*]indole-3-carboxamide. (9i)**

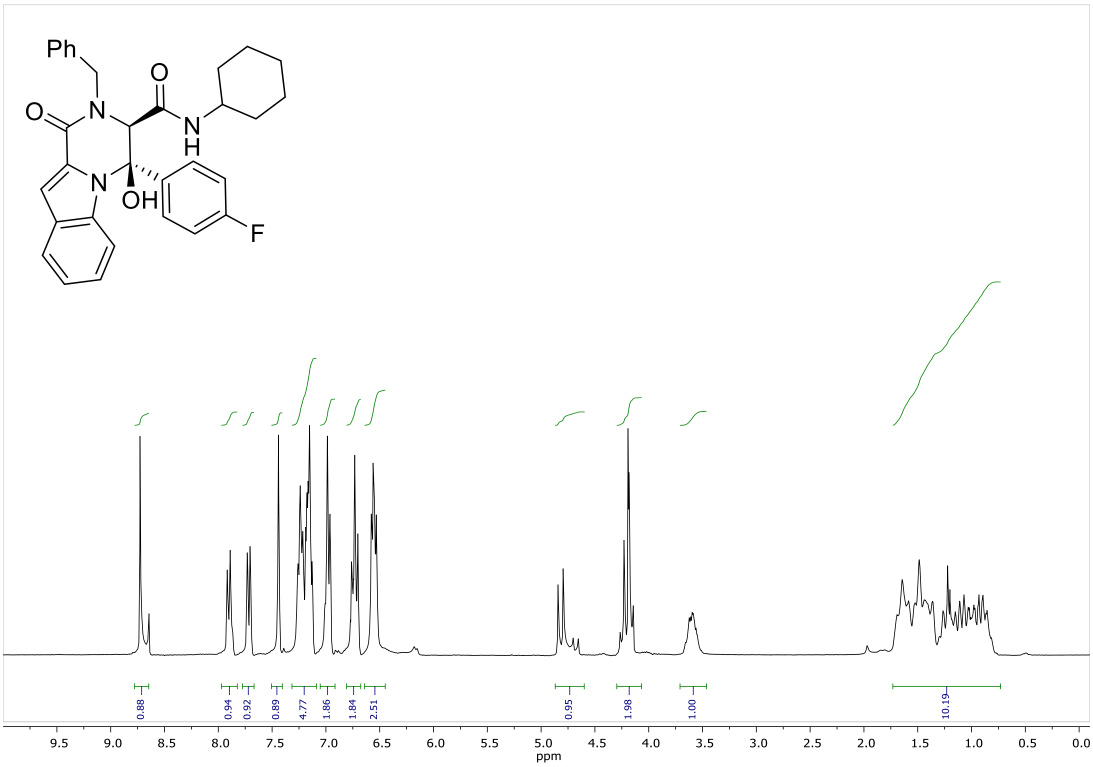

Figure S41. ^{1}H NMR spectrum (300 MHz, $CDCl_3$)

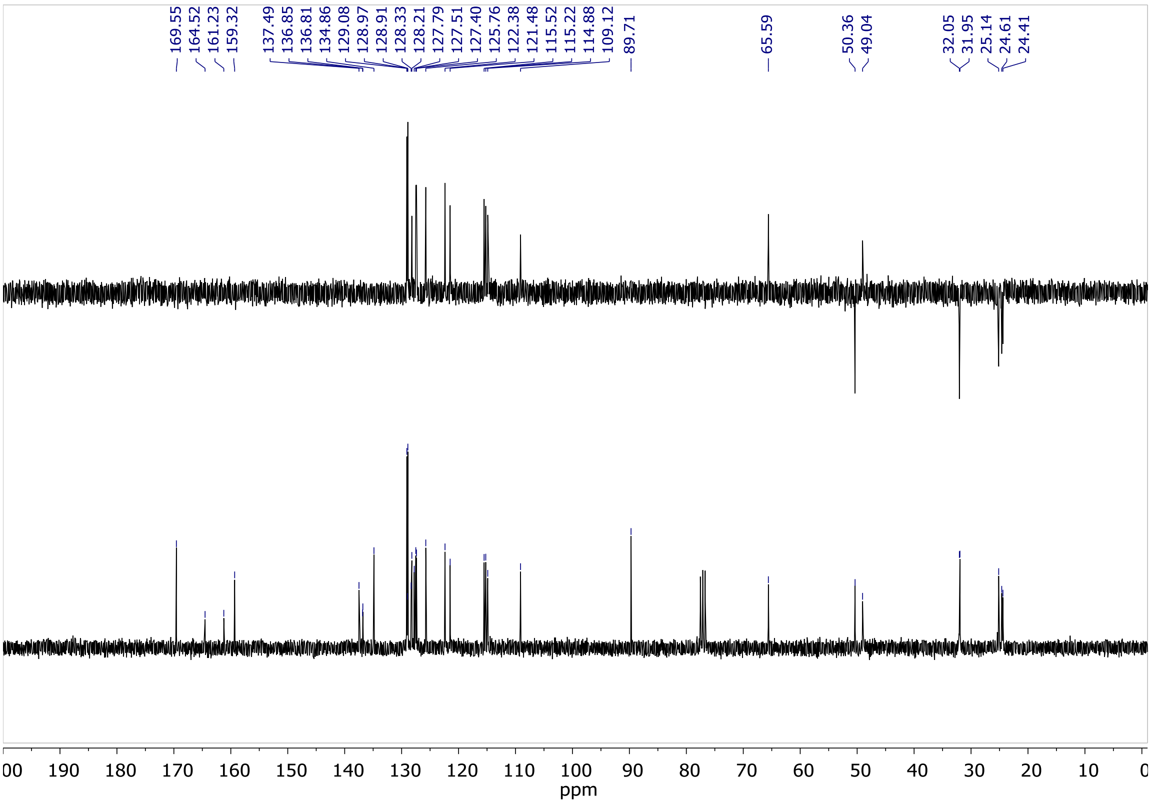

Figure S42. $^{13}C\{^{1}H\}$ and DEPT-135 NMR spectra (75 MHz, $CDCl_3$)

(3*R,4*R**)-*N*,2-di-*tert*-Butyl-4-hydroxy-1-oxo-4-phenyl-1,2,3,4-tetrahydropyrazino[1,2-*a*]indole-3-carboxamide. (9j)**

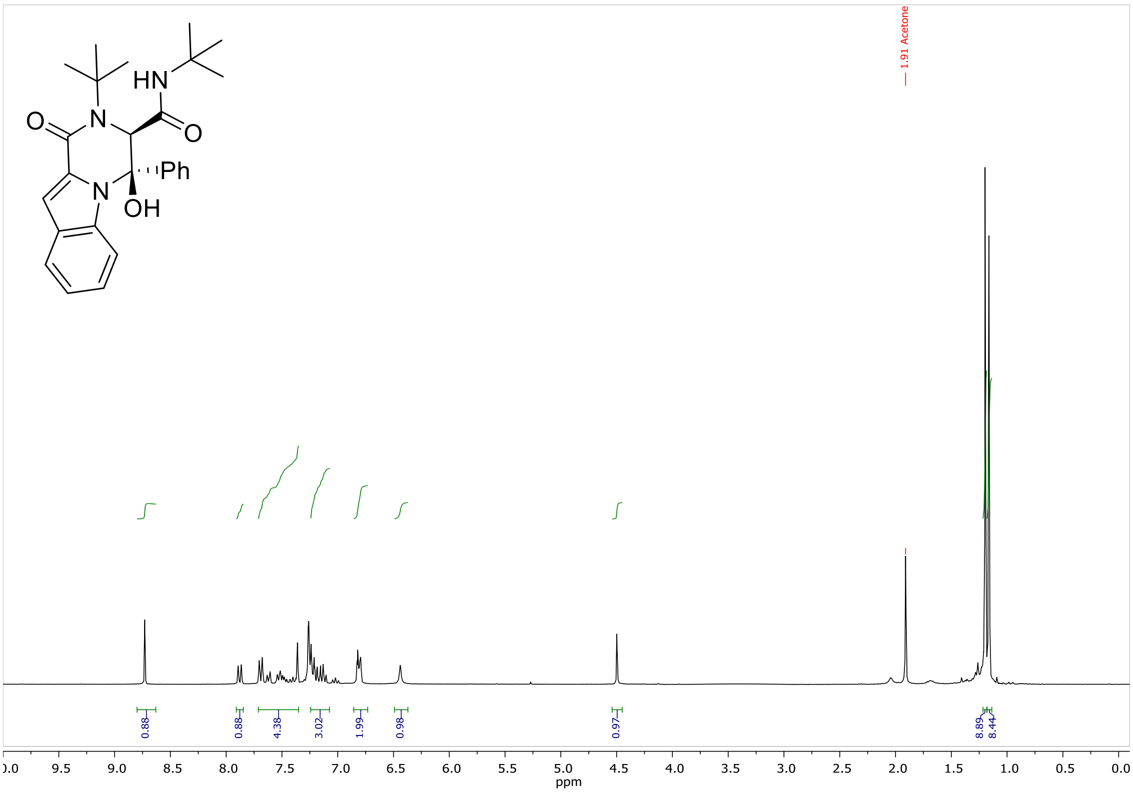

Figure S43. ^{1}H NMR spectrum (300 MHz, $CDCl_3$)

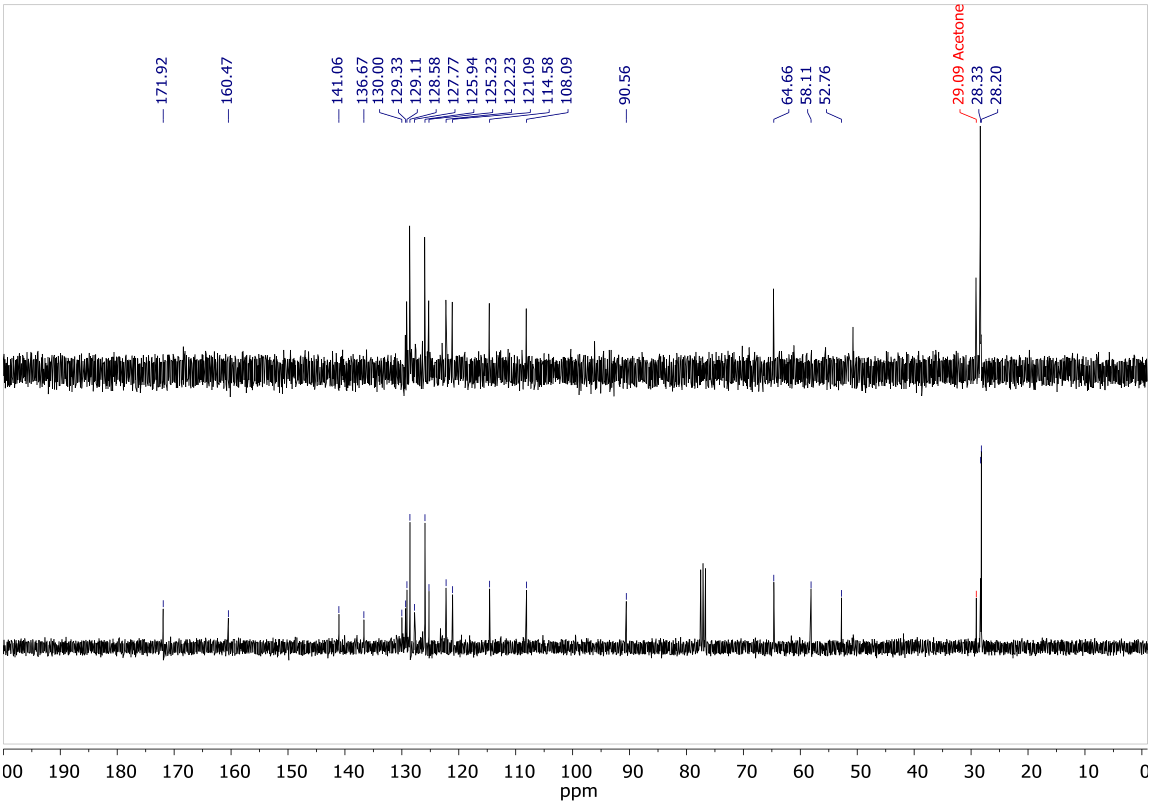

Figure S44. $^{13}C\{^{1}H\}$ and DEPT-135 NMR spectra (75 MHz, $CDCl_3$)

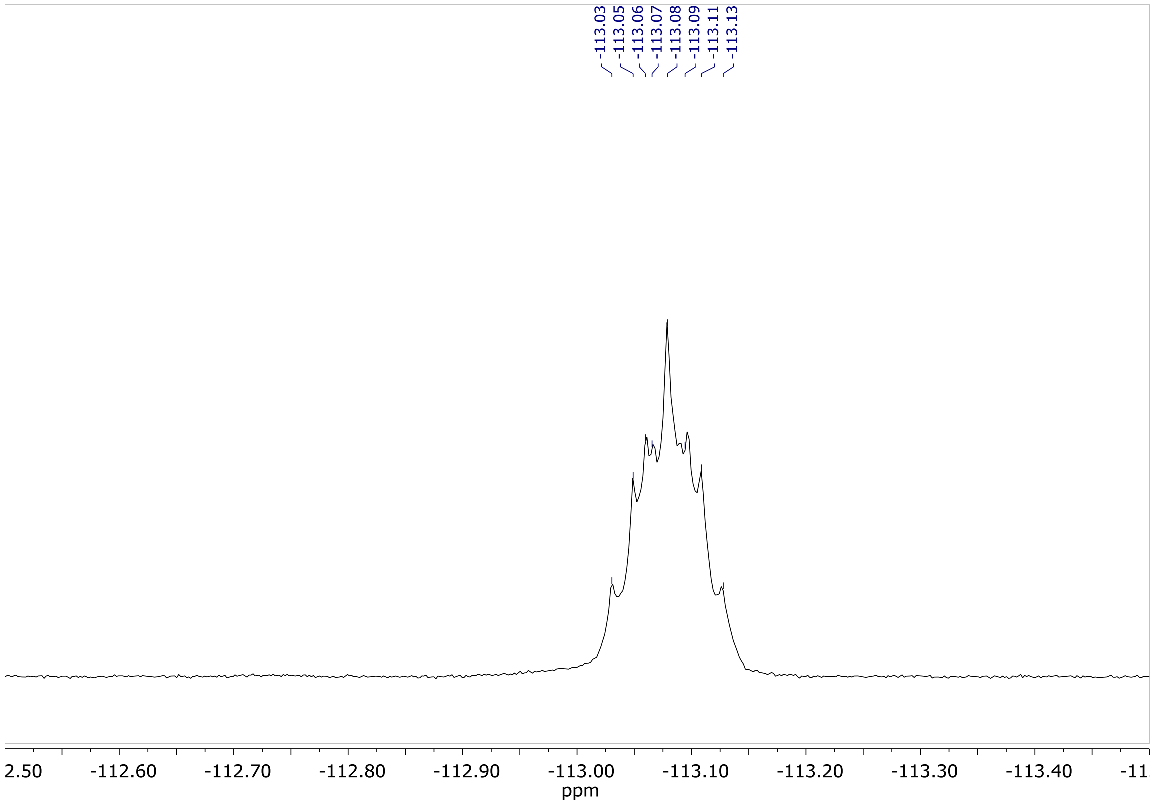

Figure S45. ^{19}F NMR spectrum (282 MHz, $CDCl_3$)

(3*R,4*R**)-2-(3-Bromopropyl)-*N*-(*tert*-butyl)-4-hydroxy-1-oxo-4-phenyl-1,2,3,4-tetrahydropyrazino[1,2-*a*]indole-3-carboxamide. (9k)**

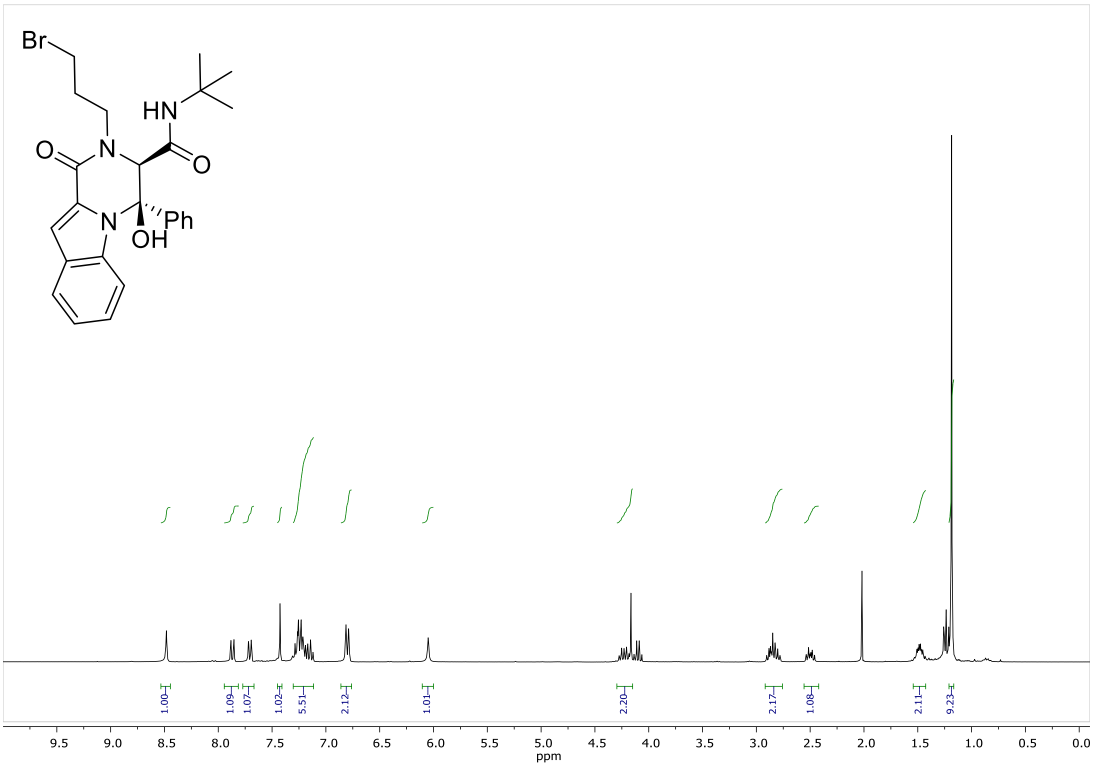

Figure S46. ^{1}H NMR spectrum (300 MHz, $CDCl_3$).

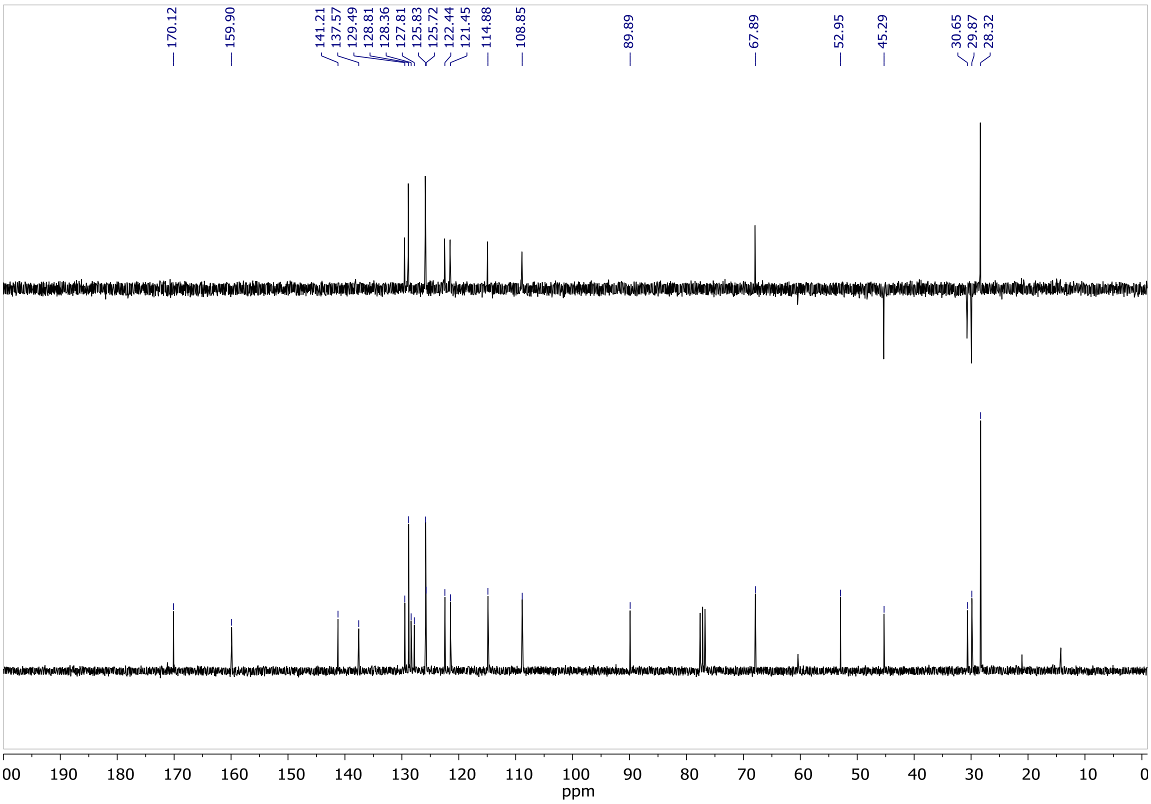

Figure S47. $^{13}C\{^{1}H\}$ and DEPT-135 NMR spectra (75 MHz, $CDCl_3$).

(3*R,4*R**)-*N*-Cyclohexyl-4-hydroxy-2-(2-nitrobenzyl)-1-oxo-4-phenyl-1,2,3,4-tetrahydropyrazino[1,2-*a*]indole-3-carboxamide. (9l)**

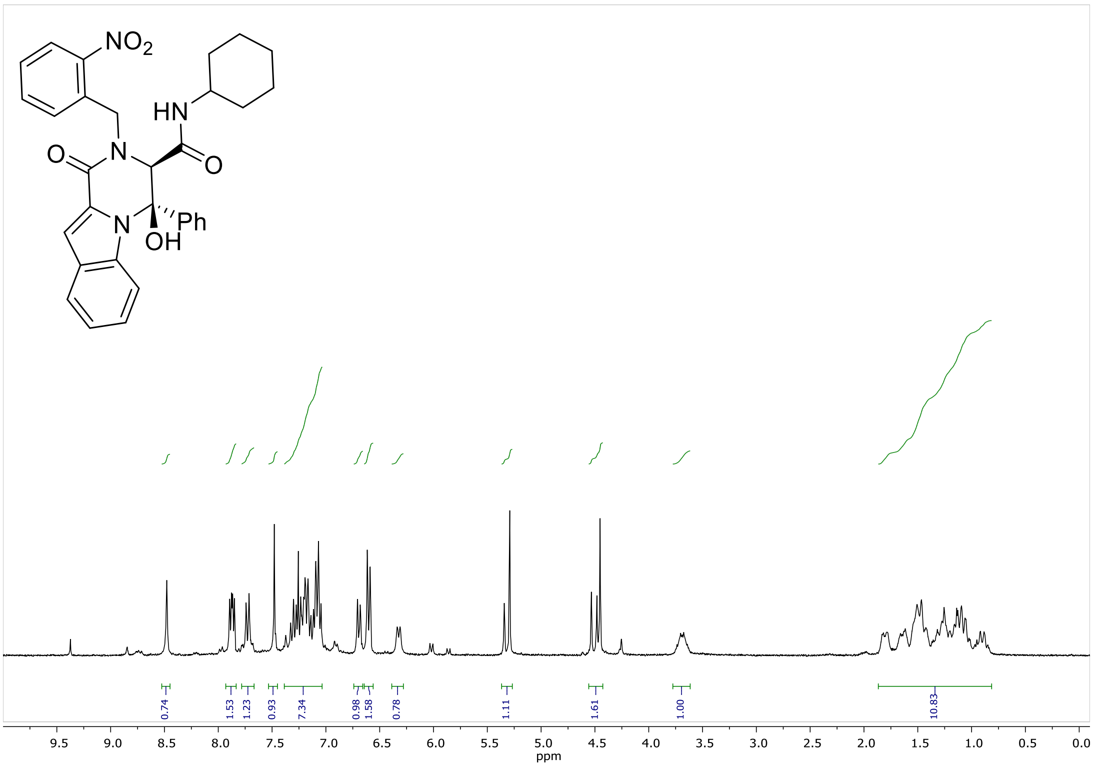

Figure S48. ^{1}H NMR spectrum (300 MHz, $CDCl_3$).

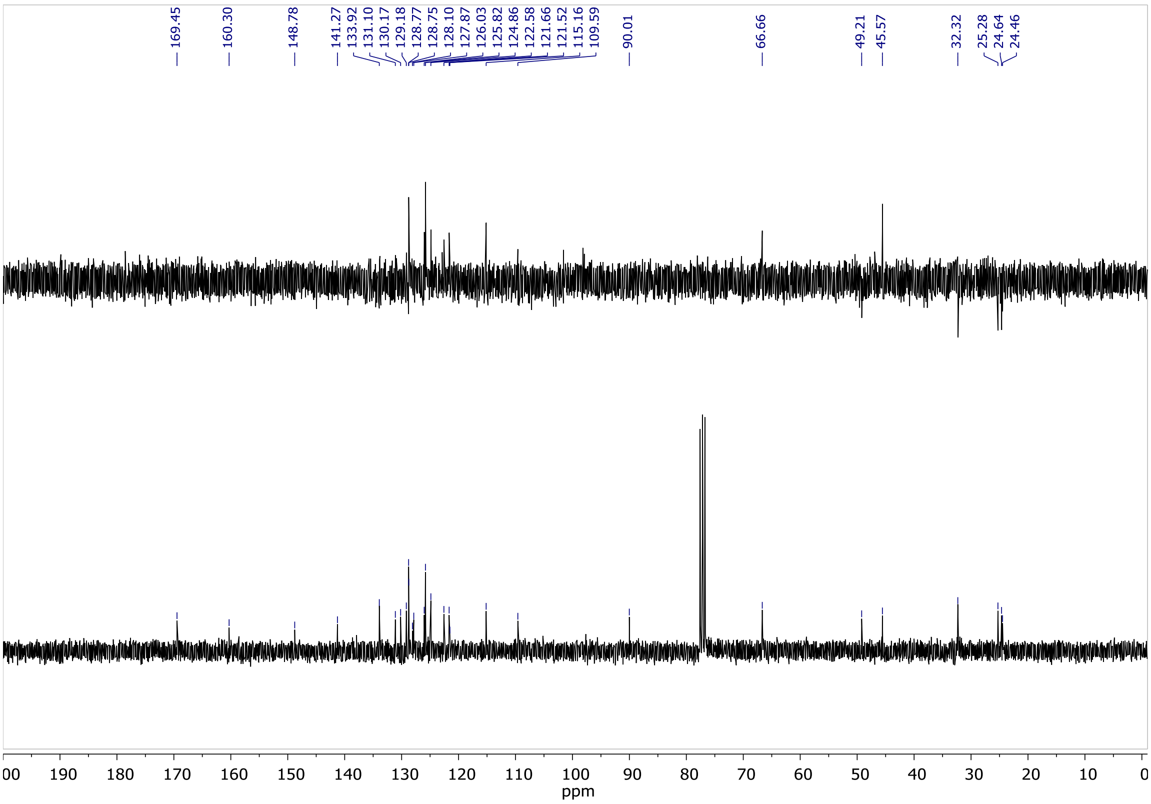

Figure S49. $^{13}C\{^{1}H\}$ and DEPT-135 NMR spectra (75 MHz, $CDCl_3$).

(3*R**,4*R**)-*N*-Cyclohexyl-4-(4-fluorophenyl)-4-hydroxy-2-(2-nitrobenzyl)-1-oxo-1,2,3,4-tetrahydropyrazino[1,2-*a*]indole-3-carboxamide. (9m)

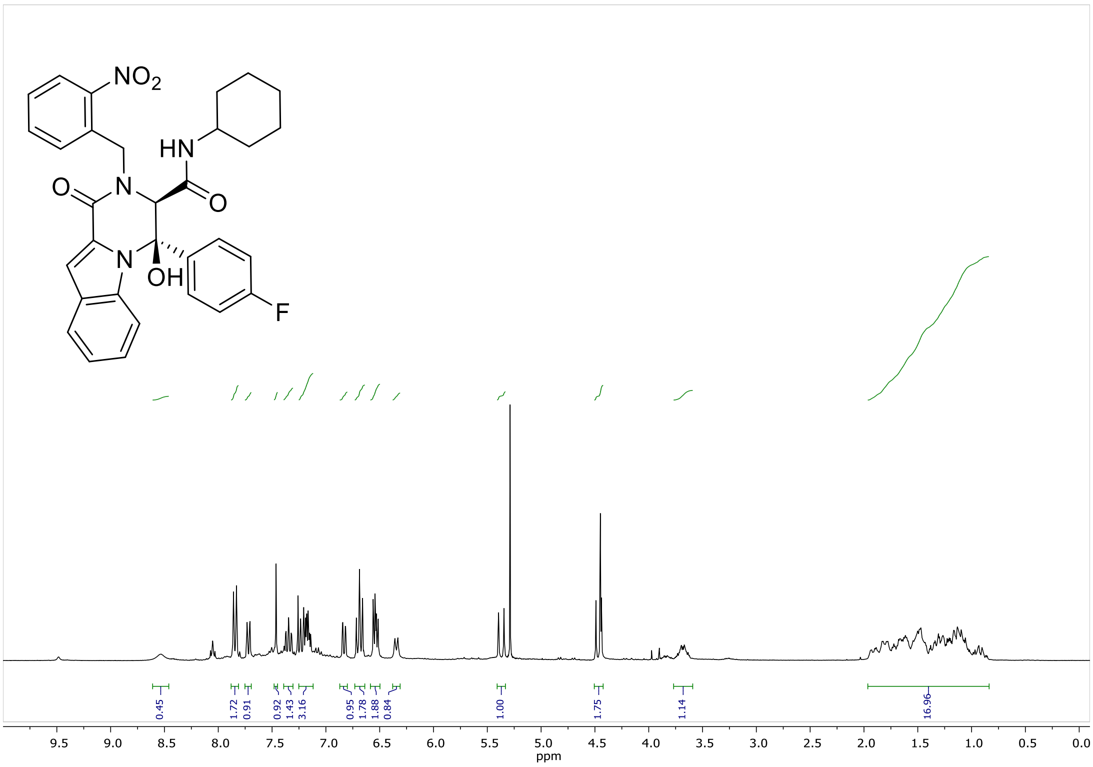

Figure S50. ^{1}H NMR spectrum (300 MHz, $CDCl_3$)

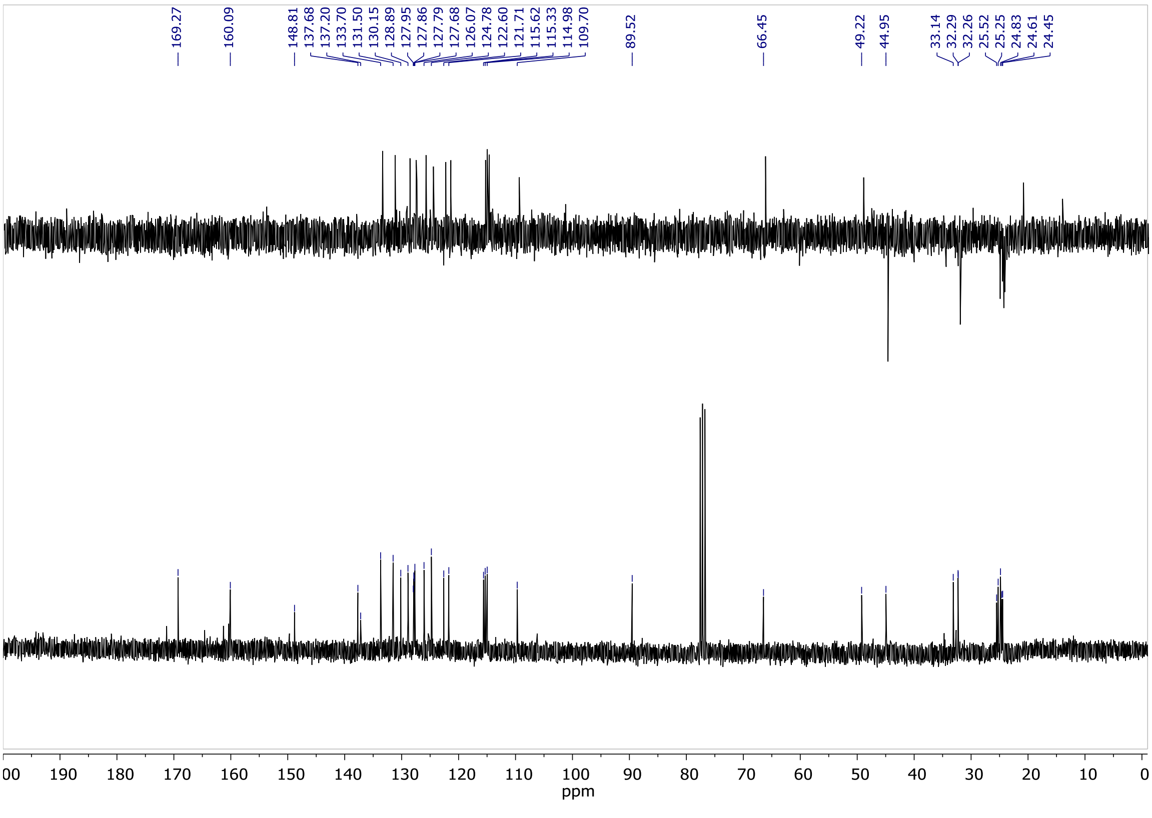

Figure S51. $^{13}C\{^{1}H\}$ and DEPT-135 NMR spectra (75 MHz, $CDCl_3$)

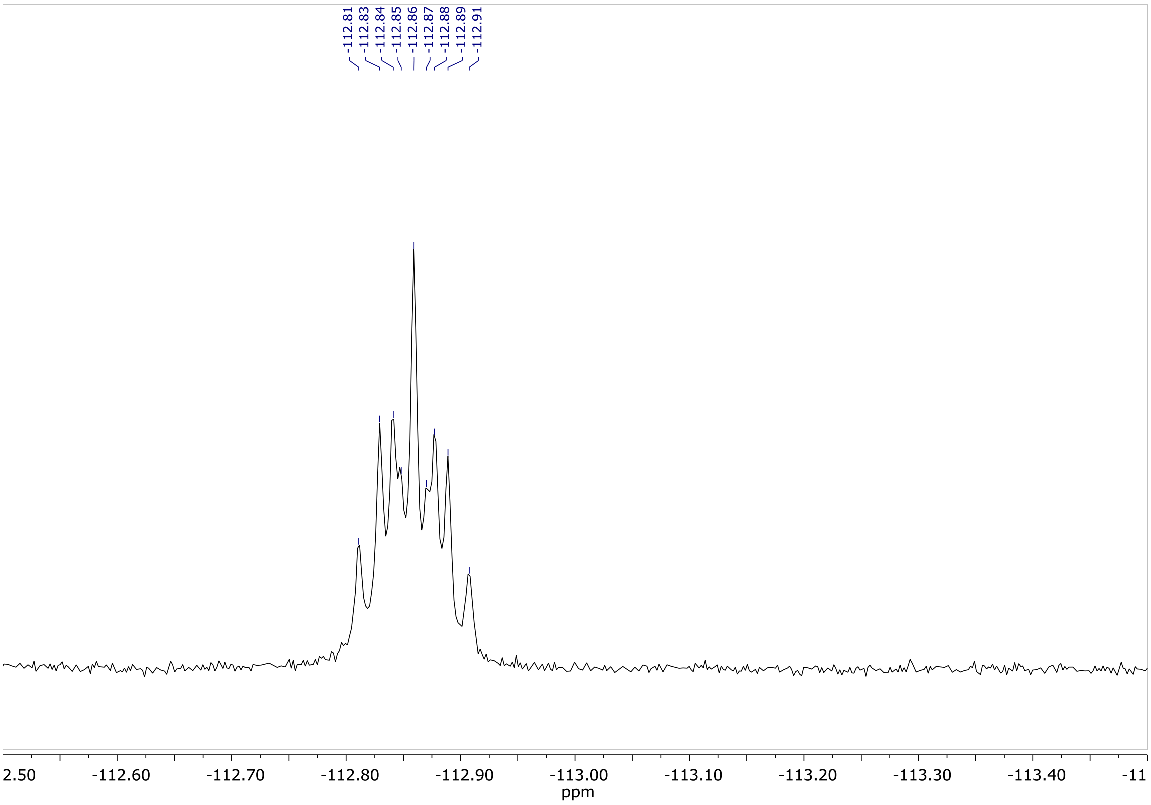

Figure S52. ^{19}F NMR spectrum (282 MHz, $CDCl_3$)

(3*R,4*R**)-*N*-(*tert*-Butyl)-4-hydroxy-4-(4-methoxyphenyl)-2-(2-nitrobenzyl)-1-oxo-1,2,3,4-tetrahydropyrazino[1,2-*a*]indole-3-carboxamide. (9n)**

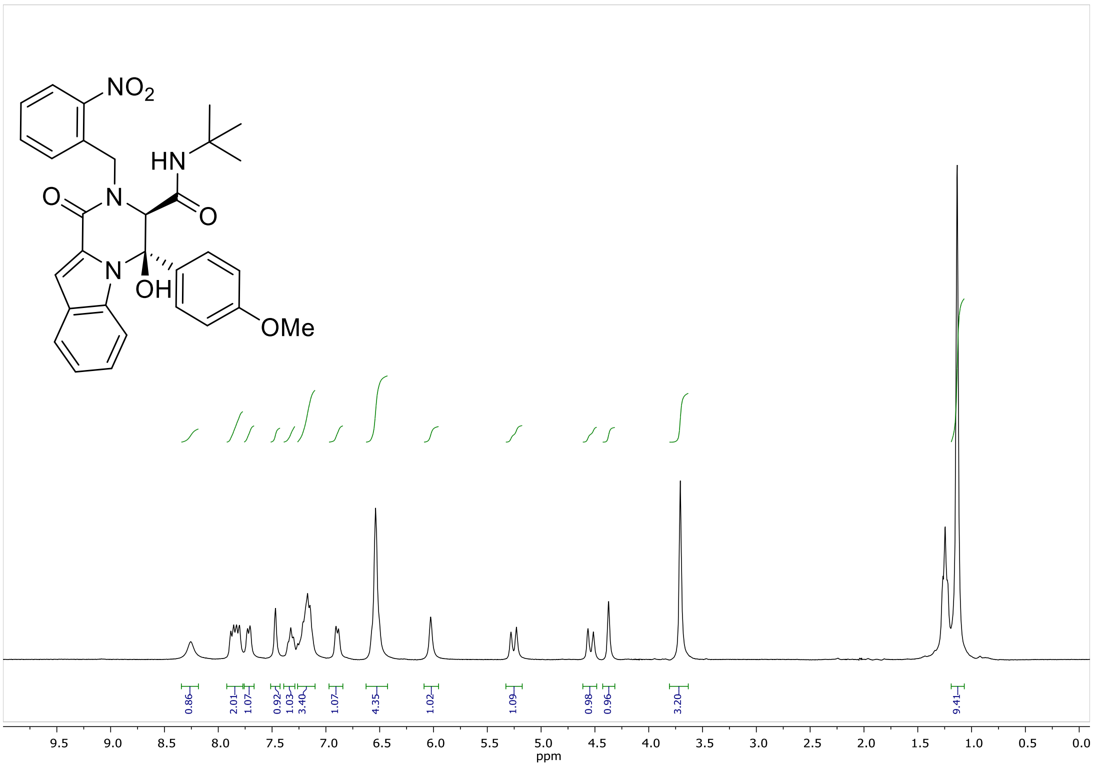

Figure S53. ^{1}H NMR spectrum (300 MHz, $CDCl_3$).

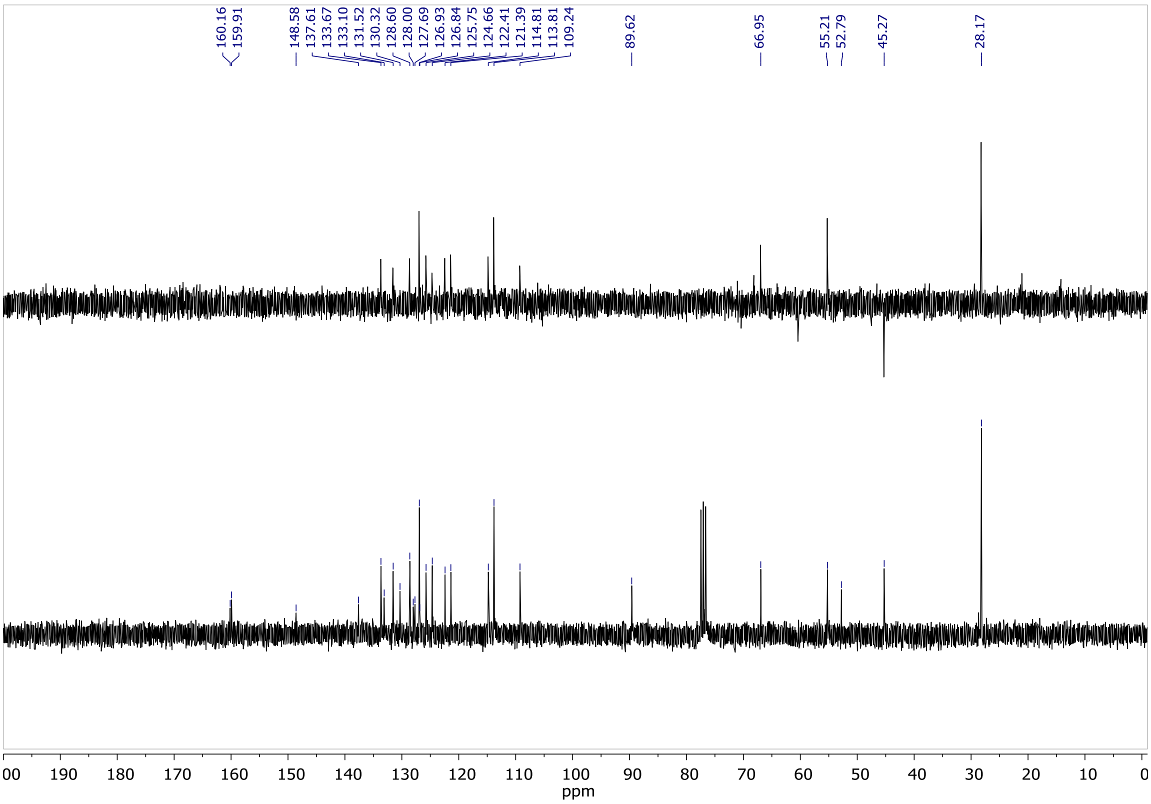

Figure S54. $^{13}C\{^{1}H\}$ and DEPT-135 NMR spectra (75 MHz, $CDCl_3$).

(3*R,4*R**)-*N*-(*tert*-Butyl)-4-hydroxy-2-(2-nitrobenzyl)-1-oxo-4-(4-(trifluoromethyl)phenyl)-1,2,3,4-tetrahydropyrazino[1,2-*a*]indole-3-carboxamide. (9o)**

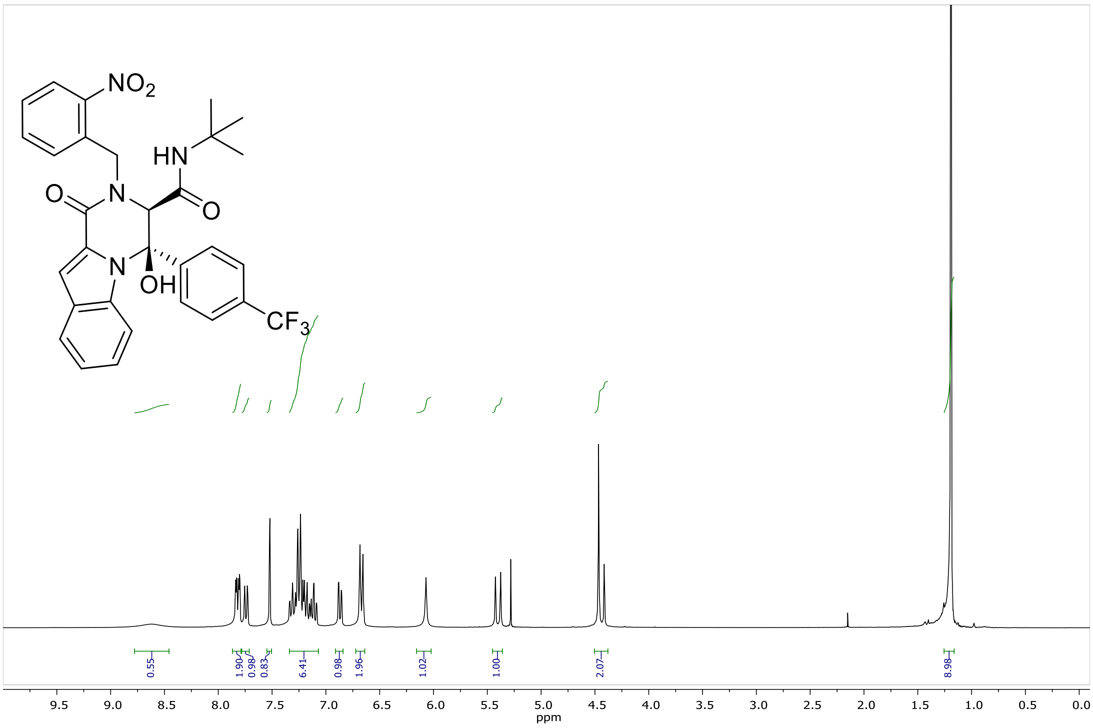

Figure S55. ^{1}H NMR spectrum (300 MHz, $CDCl_3$).

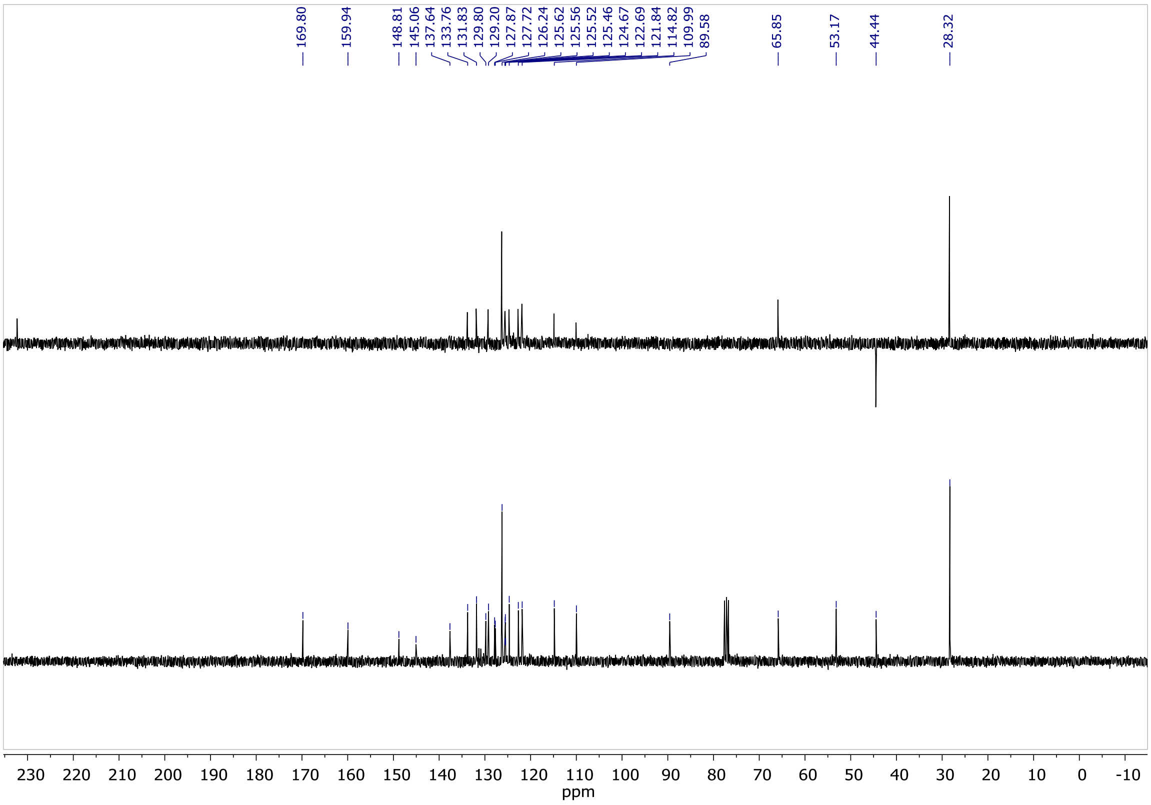

Figure S56. $^{13}C\{^{1}H\}$ and DEPT-135 NMR spectra (75 MHz, $CDCl_3$).

1-Benzyl-*N*-cyclohexyl-6-oxo-3,4-diphenyl-1,4,5,6-tetrahydropyrazine-2-carboxamide. (10a)

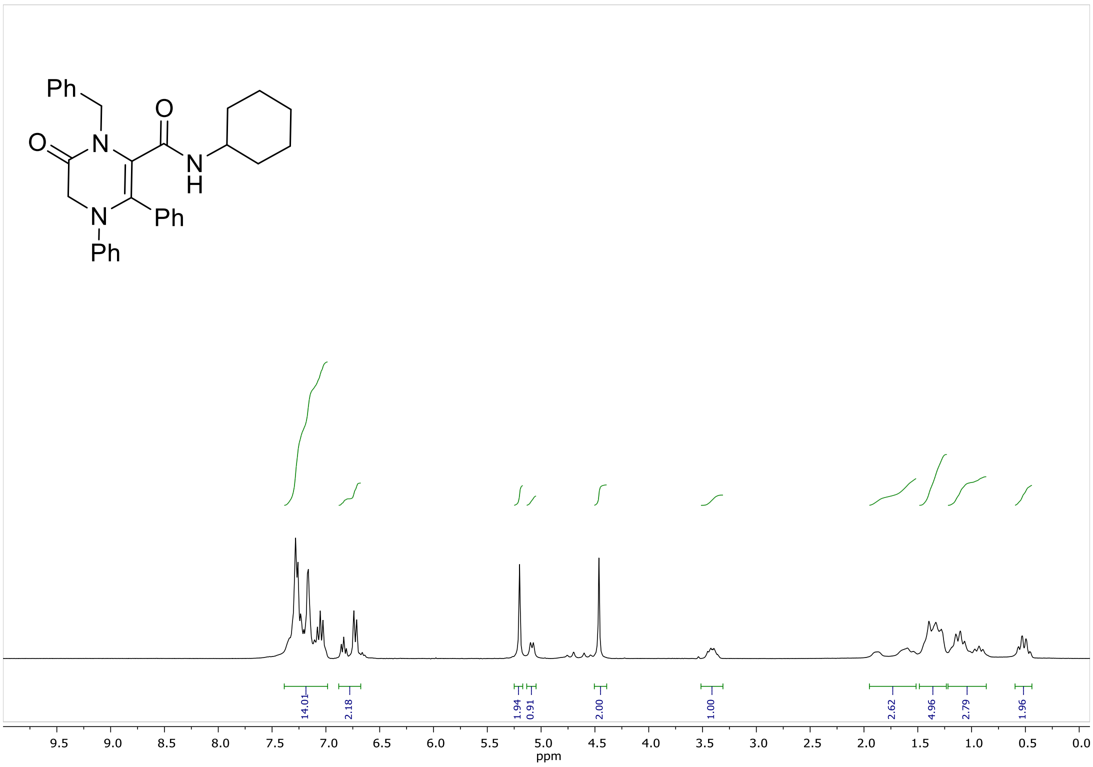

Figure S57. ^{1}H NMR spectrum (300 MHz, $CDCl_3$).

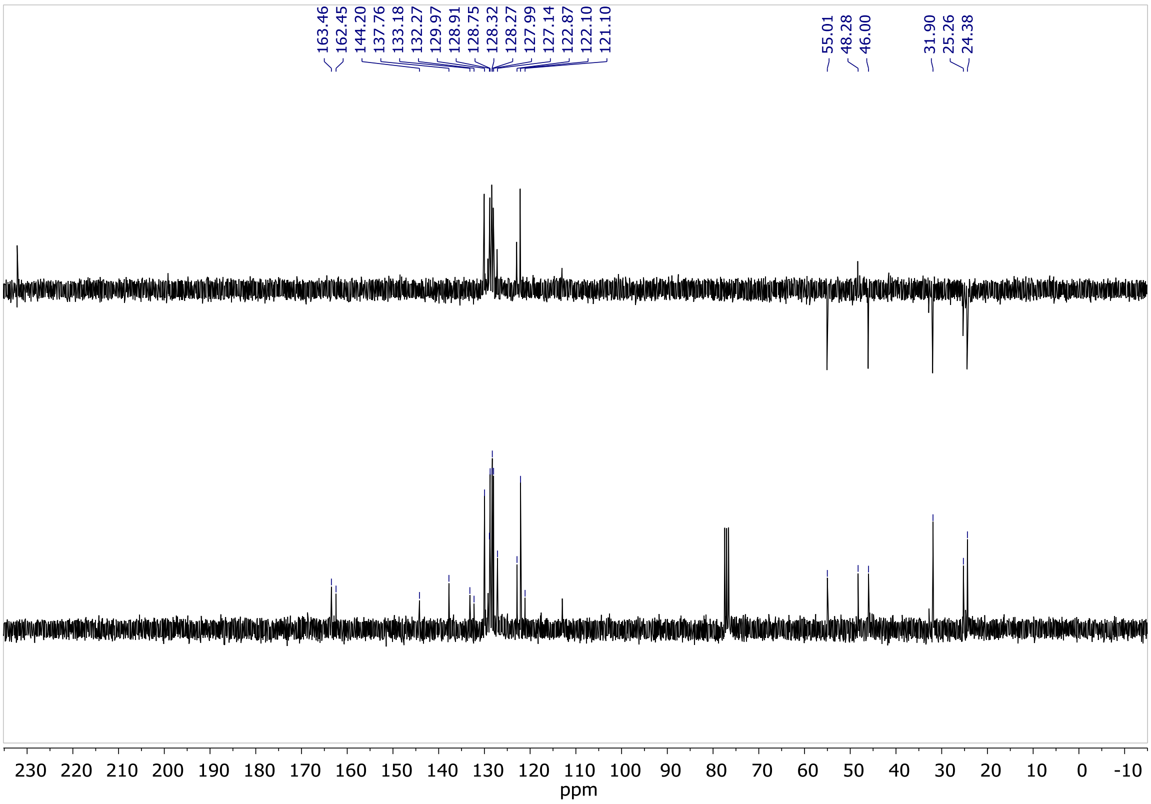

Figure S58. $^{13}C\{^1H\}$ and DEPT-135 NMR spectra (75 MHz, $CDCl_3$).

1-Benzyl-*N*-cyclohexyl-3-(4-fluorophenyl)-6-oxo-4-phenyl-1,4,5,6-tetrahydropyrazine-2-carboxamide. (10b)

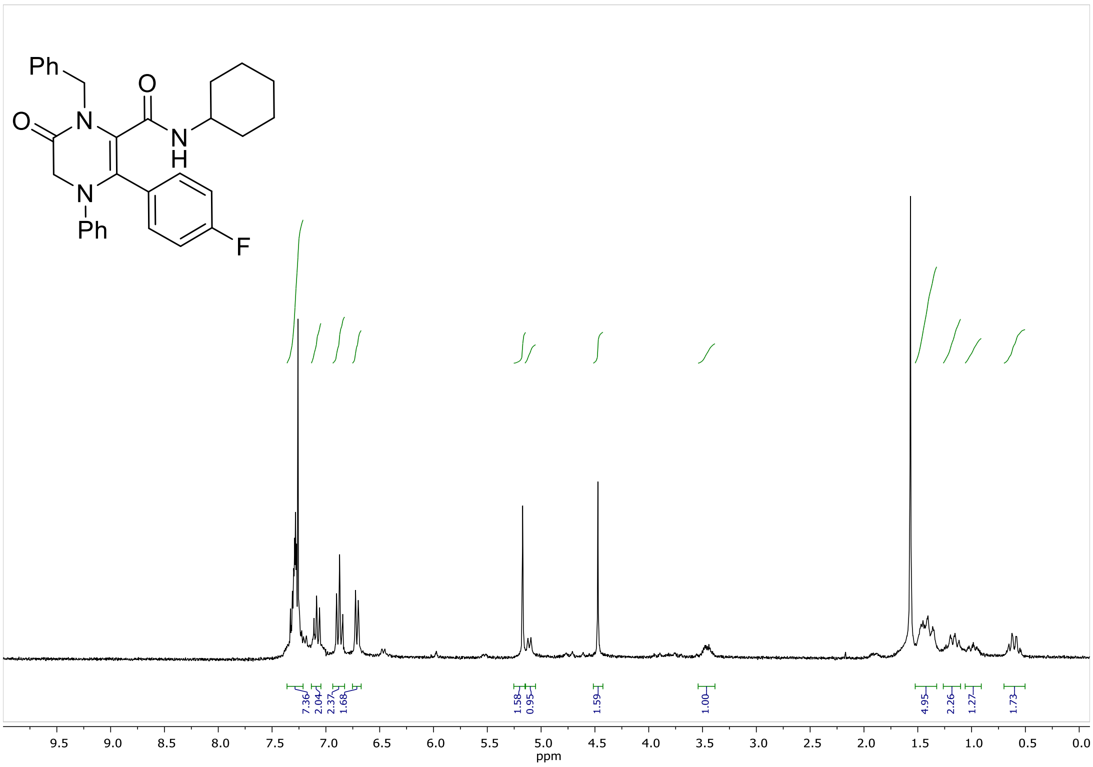

Figure S59. ^{1}H NMR spectrum (300 MHz, $CDCl_3$).

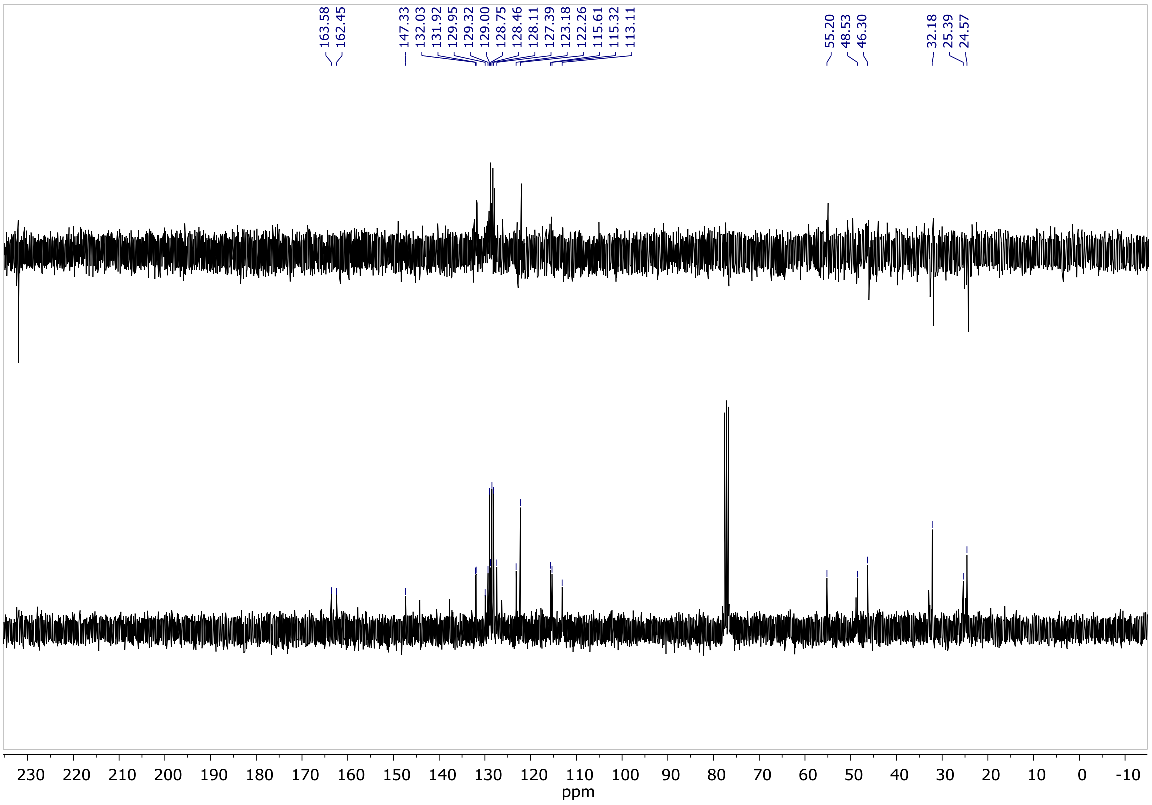

Figure S560. $^{13}C\{^{1}H\}$ and DEPT-135 NMR spectra (75 MHz, $CDCl_3$).

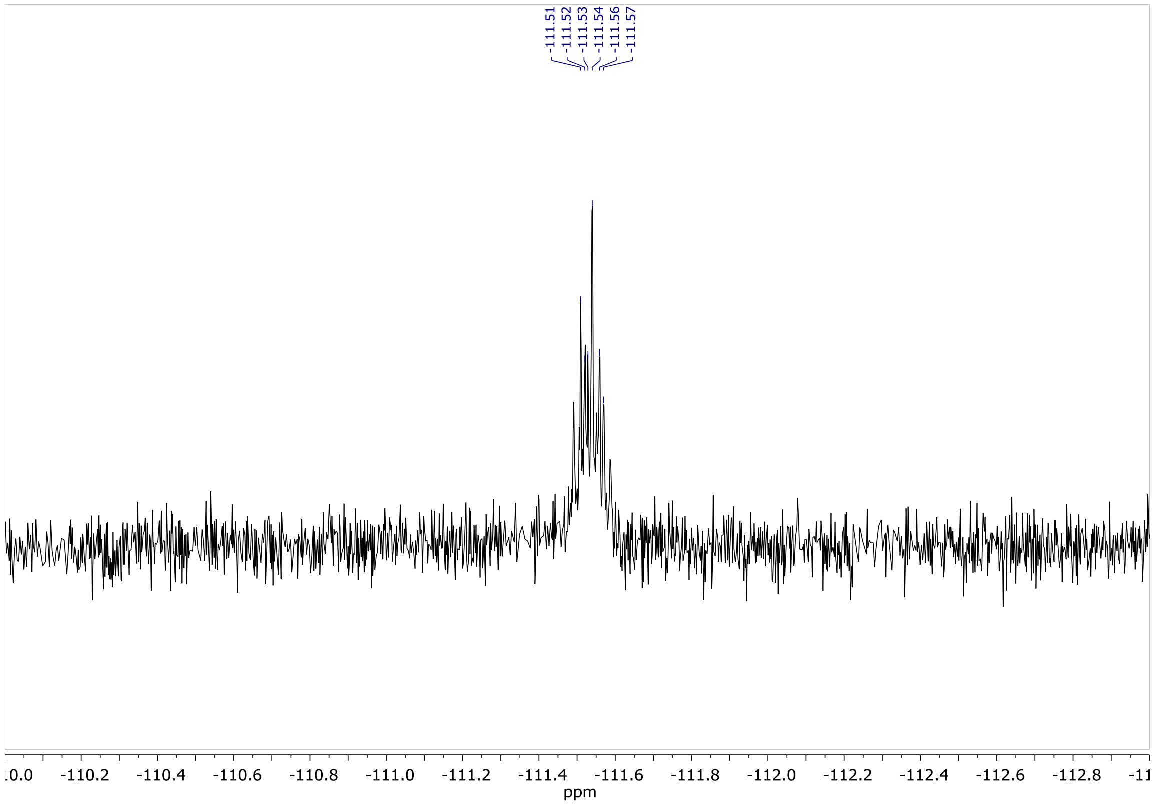

Figure S61. ^{19}F NMR spectrum (282 MHz, $CDCl_3$)

(3*R,4*R**)-*N*-(*tert*-Butyl)-4-hydroxy-2-((1*S*)-methylbenzyl)-1-oxo-4-phenyl-1,2,3,4-tetrahydropyrazino[1,2-*a*]indole-3-carboxamide. (11)**

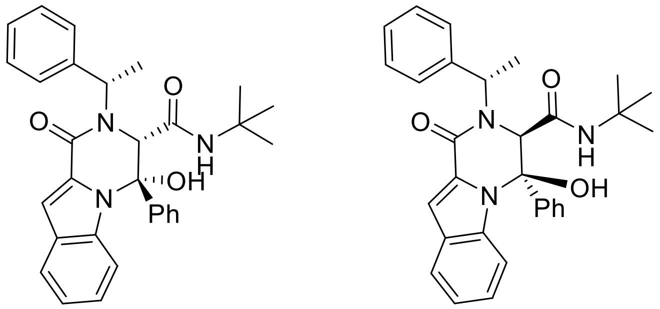

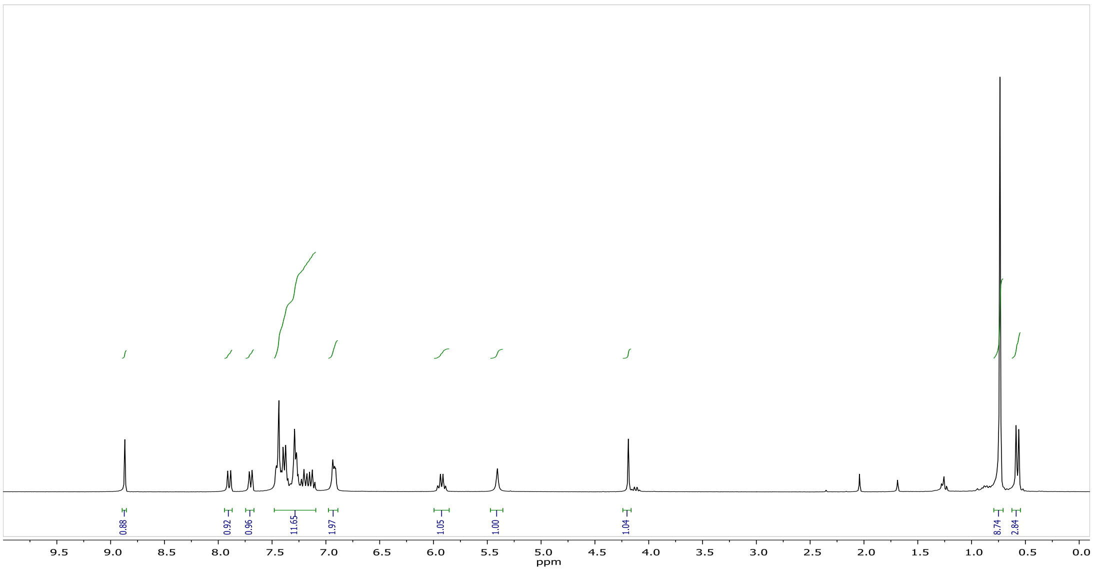

Figure S62. ^{1}H NMR spectrum (300 MHz, $CDCl_3$).

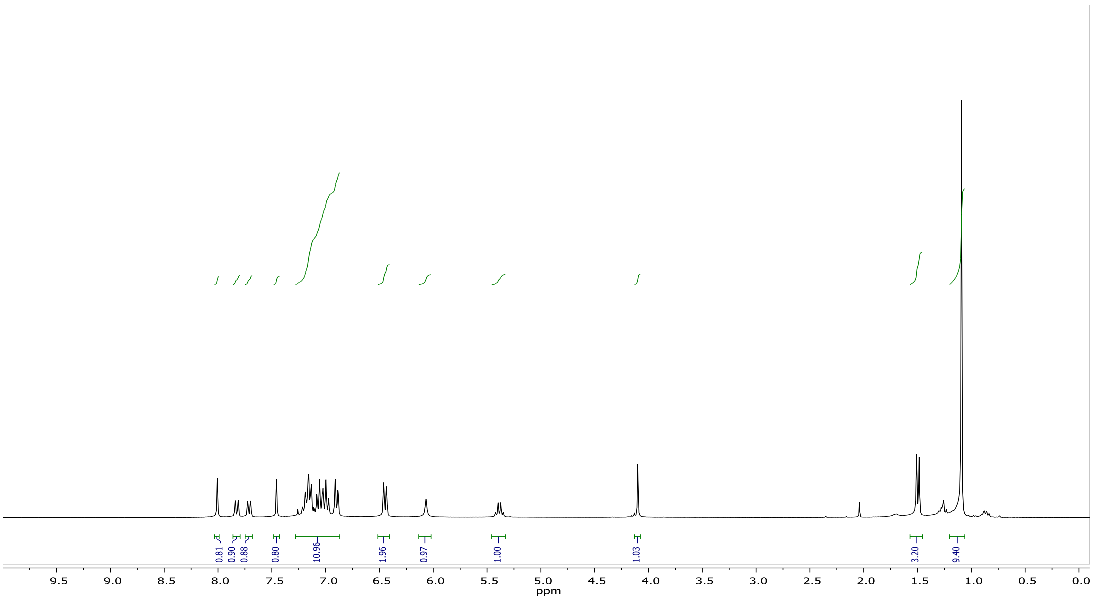

Figure S63. ^{1}H NMR spectrum (300 MHz, $CDCl_3$).

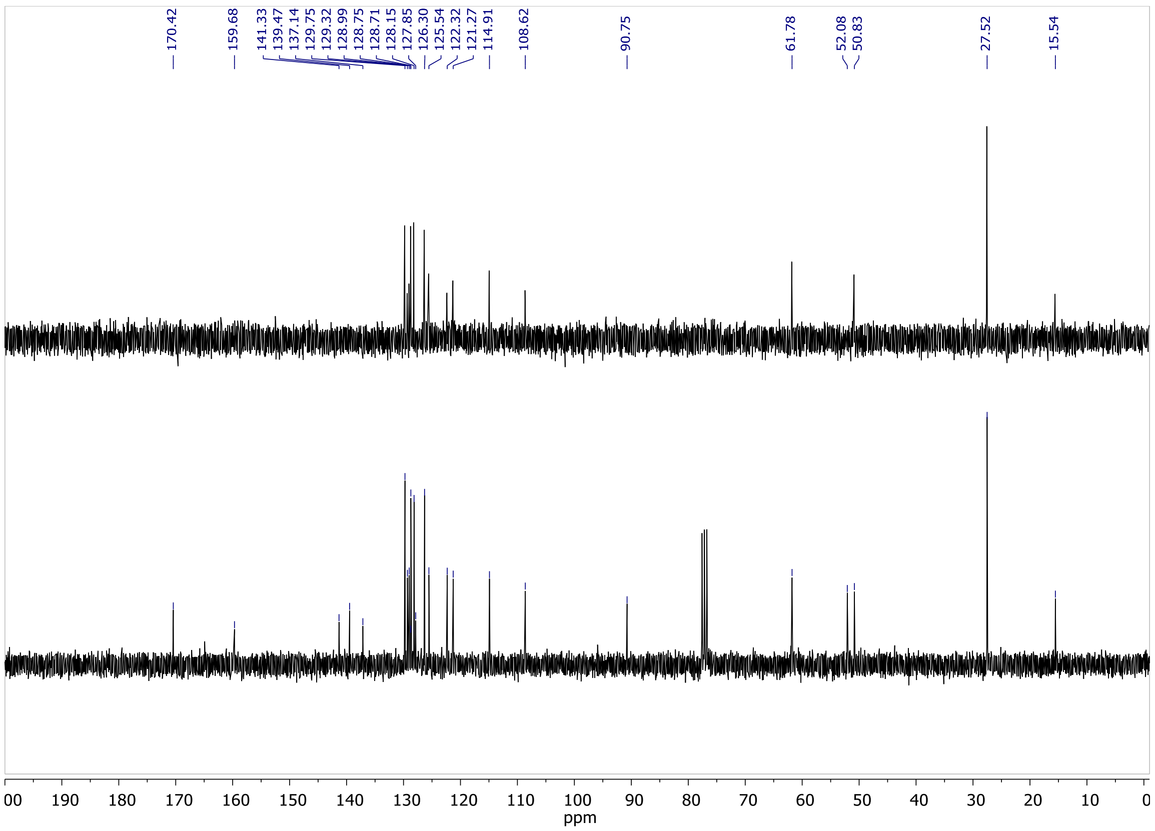

Figure S64. $^{13}C\{^{1}H\}$ and DEPT-135 NMR spectra (75 MHz, $CDCl_3$).

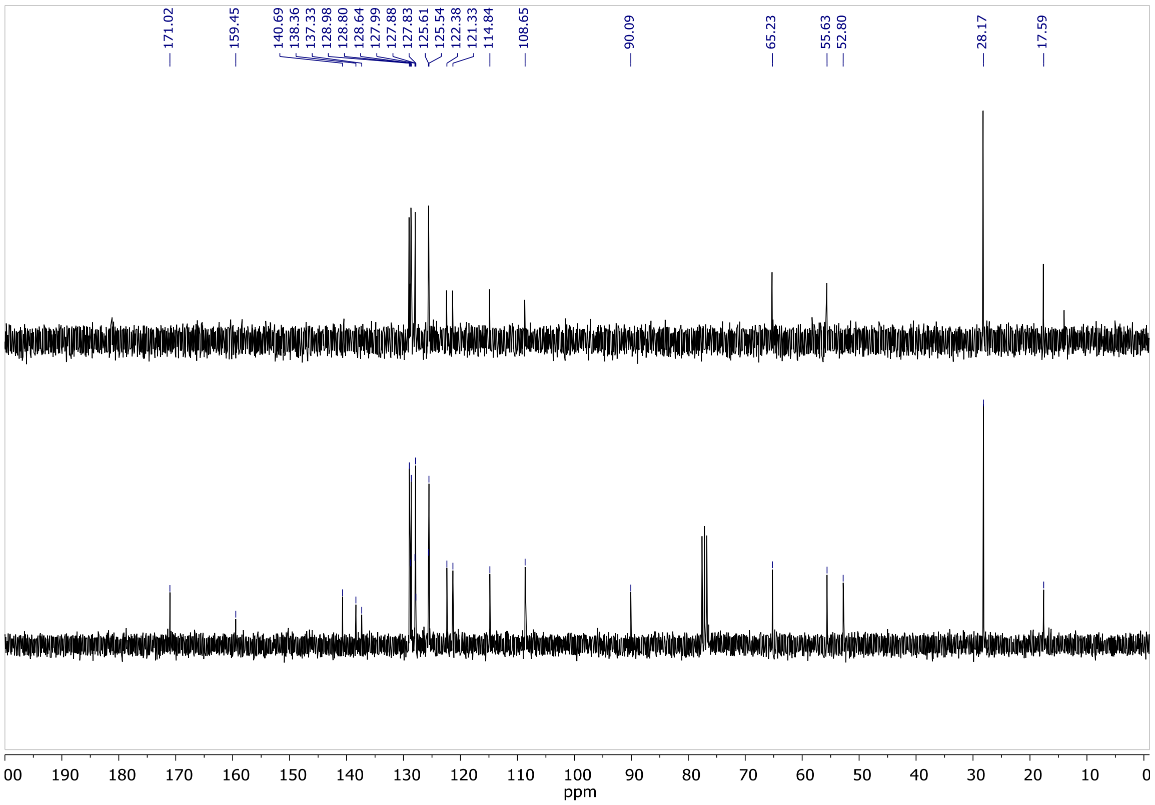

Figure S65. $^{13}C\{^{1}H\}$ and DEPT-135 NMR spectra (75 MHz, $CDCl_3$).

(5*R,6*R**)-*N*-Cyclohexyl-5-hydroxy-5-phenyl-5,6-dihydro-8*H*-pyrrolo[2',1':3,4]pyrazino[2,1-*b*]quinazoline-6-carboxamide. (13a)**

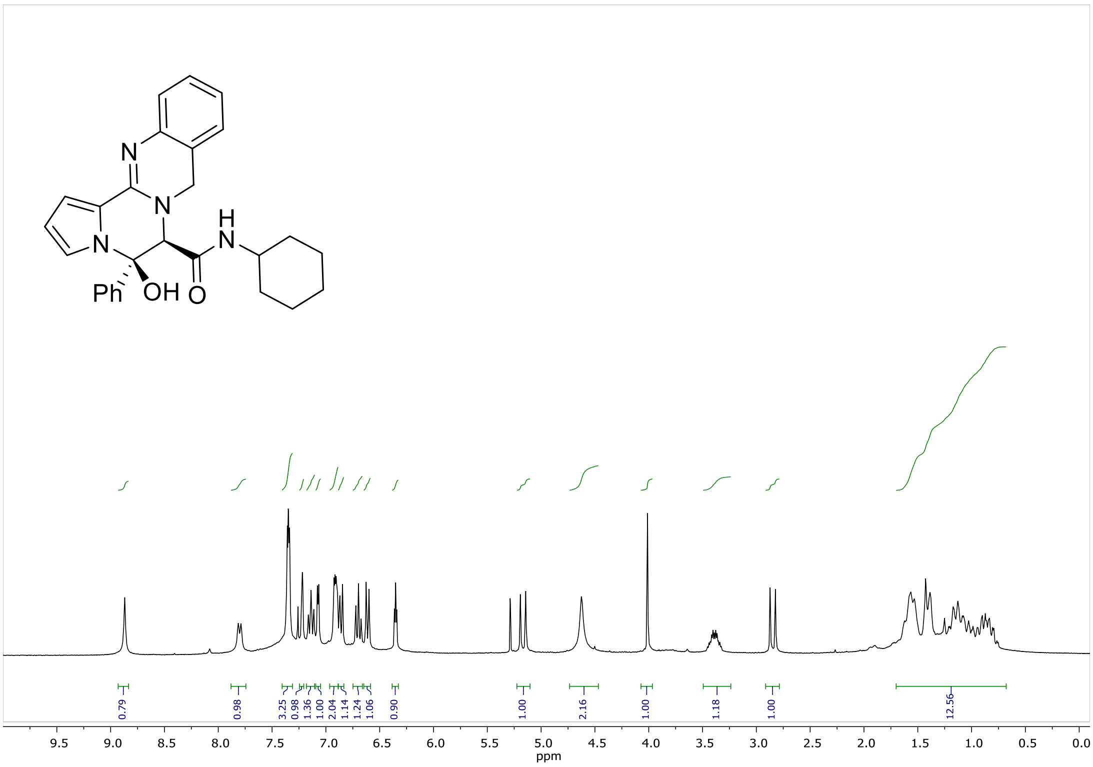

Figure S66. ^{1}H NMR spectrum (300 MHz, $CDCl_3$).

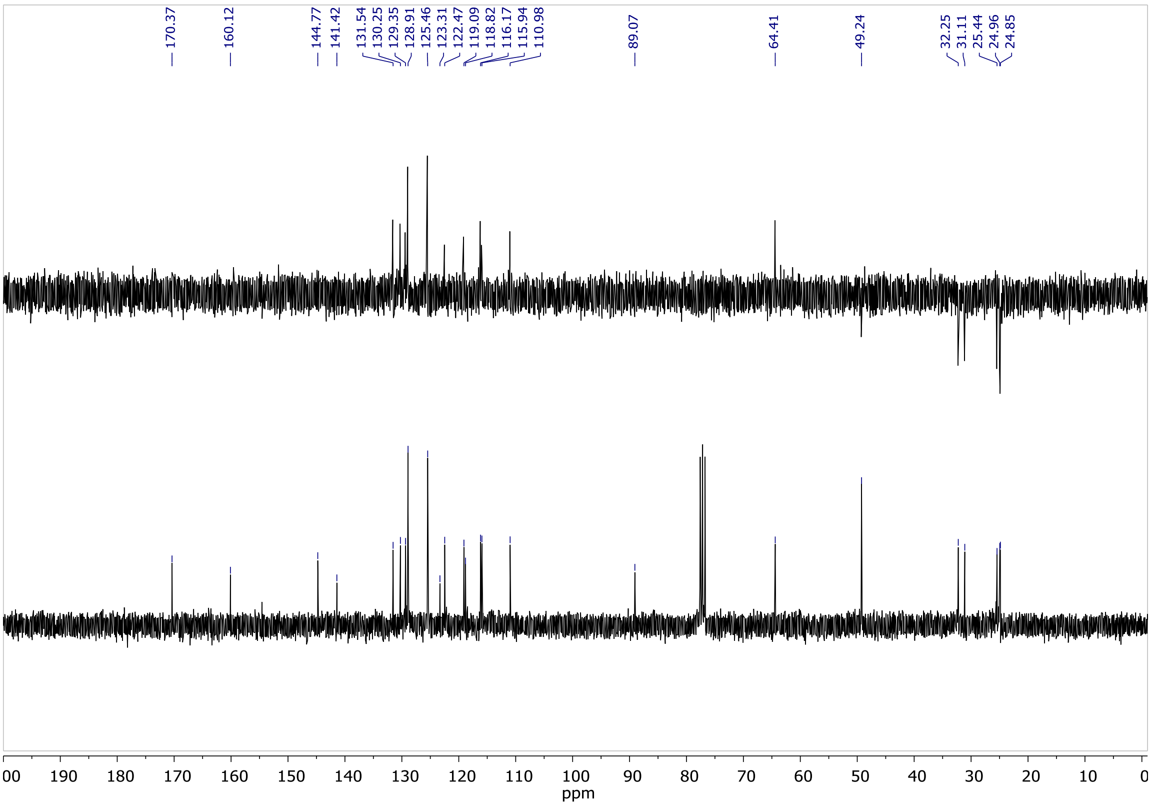

Figure S67. $^{13}C\{^1H\}$ and DEPT-135 NMR spectra (75 MHz, $CDCl_3$).

(5*R,6*R**)-*N*-(*tert*-Butyl)-5-hydroxy-5-(4-(trifluoromethyl)phenyl)-5,6-dihydro-8*H*-pyrrolo[2',1':3,4]pyrazino[2,1-*b*]quinazoline-6-carboxamide. (13b)**

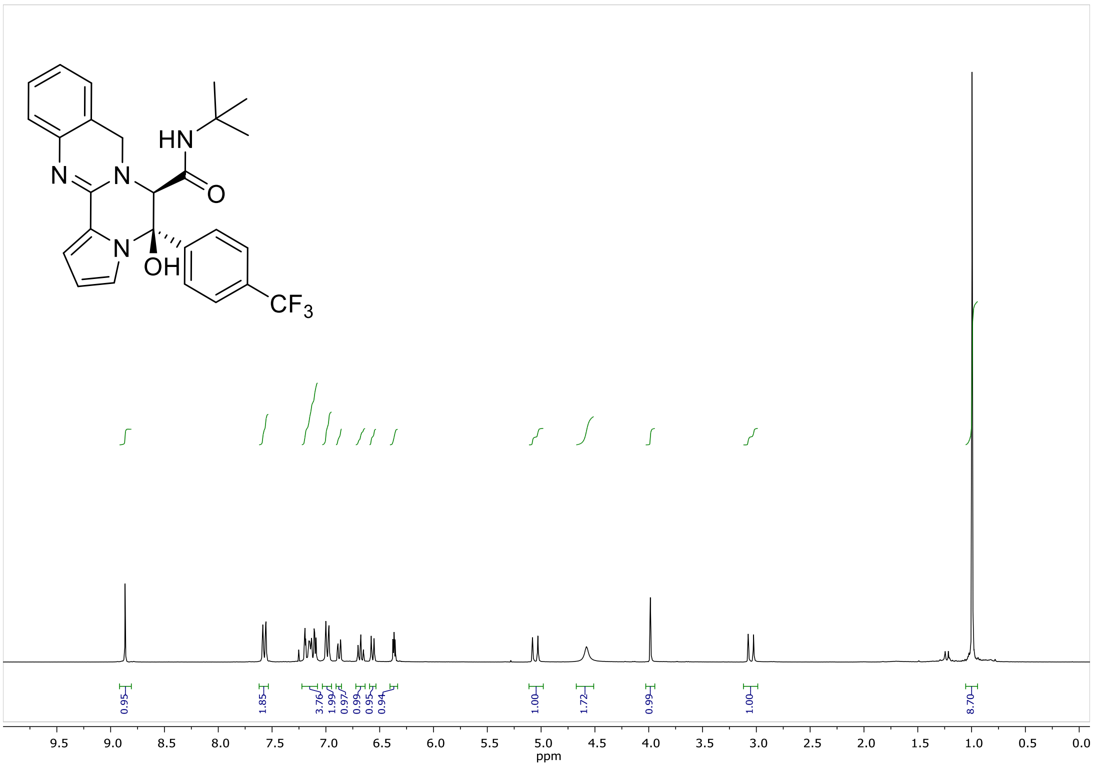

Figure S68. ^{1}H NMR spectrum (300 MHz, $CDCl_3$).

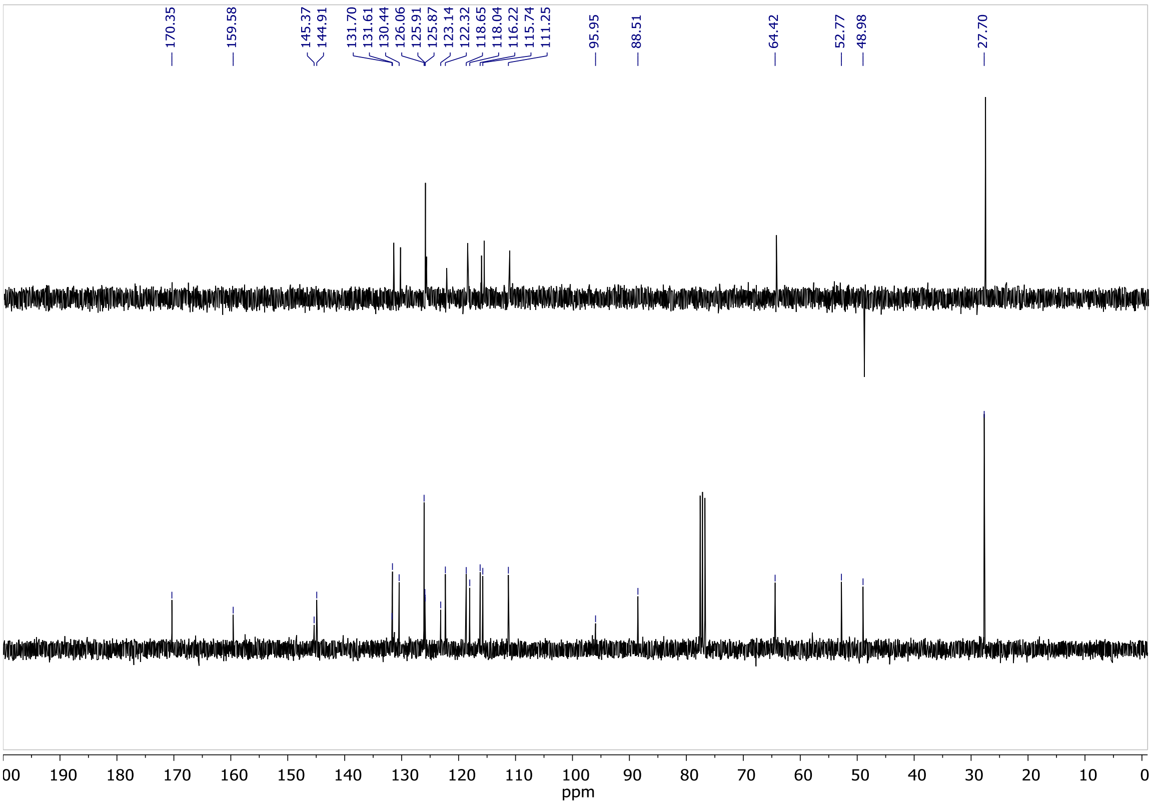

Figure S69. $^{13}C\{^{1}H\}$ and DEPT-135 NMR spectra (75 MHz, $CDCl_3$).

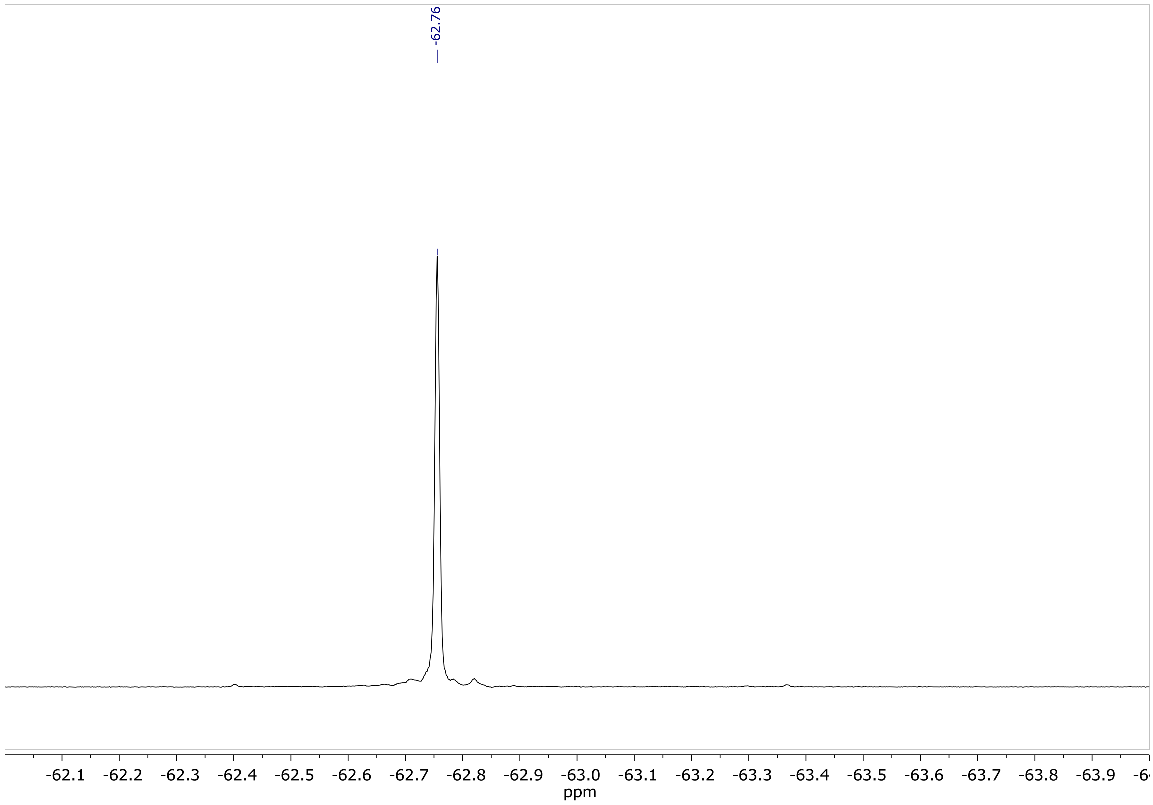

Figure S70. ^{19}F NMR spectrum (282 MHz, $CDCl_3$).

(6*R,7*R**)-*N*-Cyclohexyl-6-hydroxy-6-phenyl-6,7-dihydro-9*H*-indolo[2',1':3,4]pyrazino[2,1-*b*]quinazoline-7-carboxamide. (13c)**

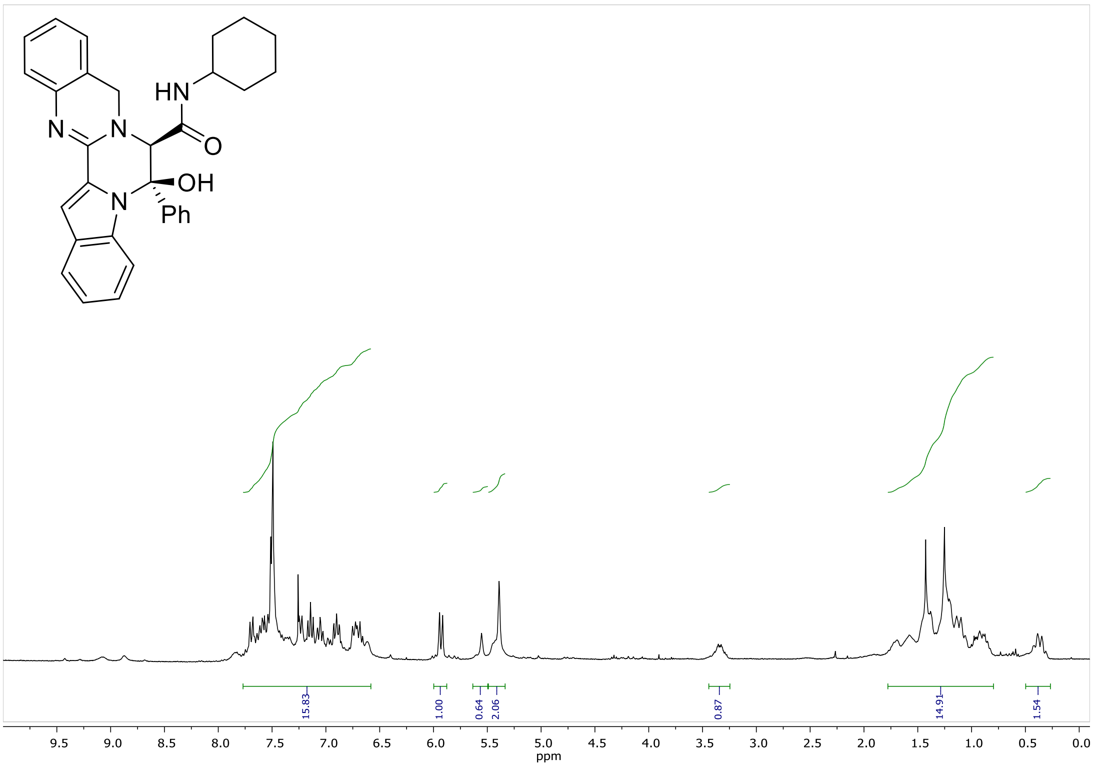

Figure S71. ^{1}H NMR spectrum (300 MHz, $CDCl_3$).

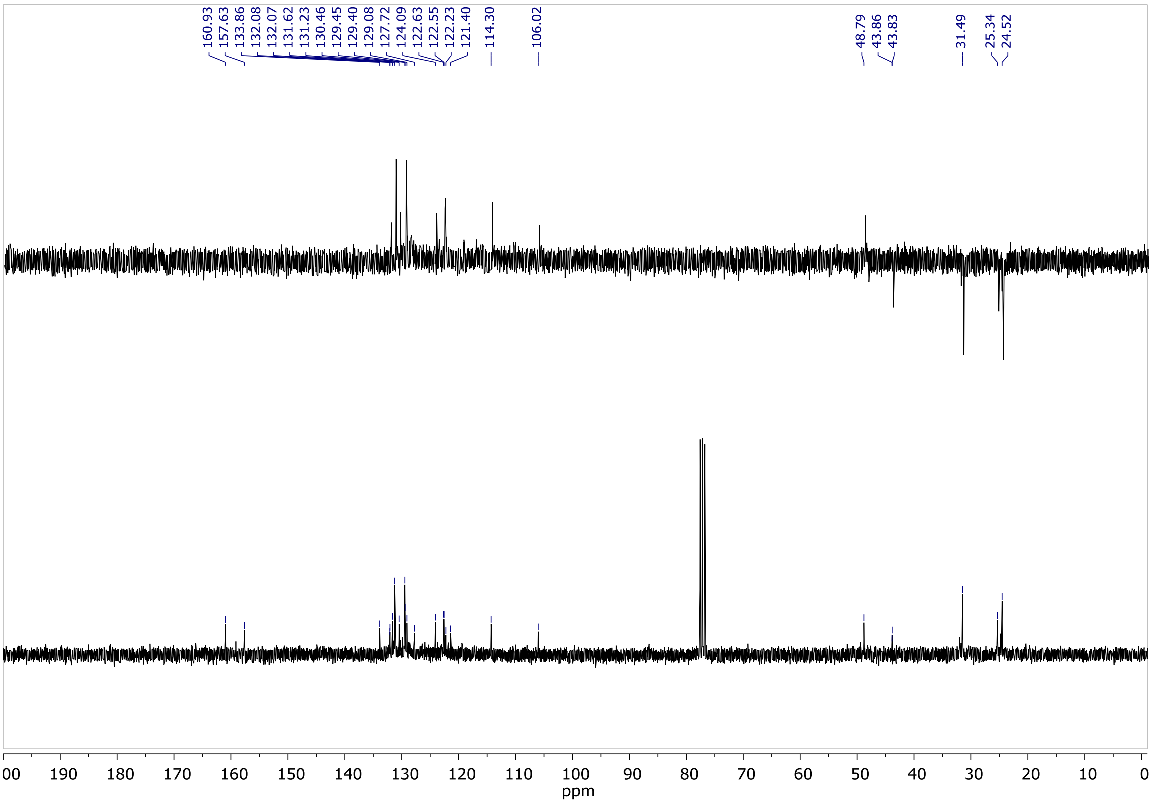

Figure S72. $^{13}C\{^{1}H\}$ and DEPT-135 NMR spectra (75 MHz, $CDCl_3$).

(6*R,7*R**)-*N*-Cyclohexyl-6-(4-fluorophenyl)-6-hydroxy-6,7-dihydro-9*H*-indolo[2',1':3,4]pyrazino[2,1-*b*]quinazoline-7-carboxamide. (13d)**

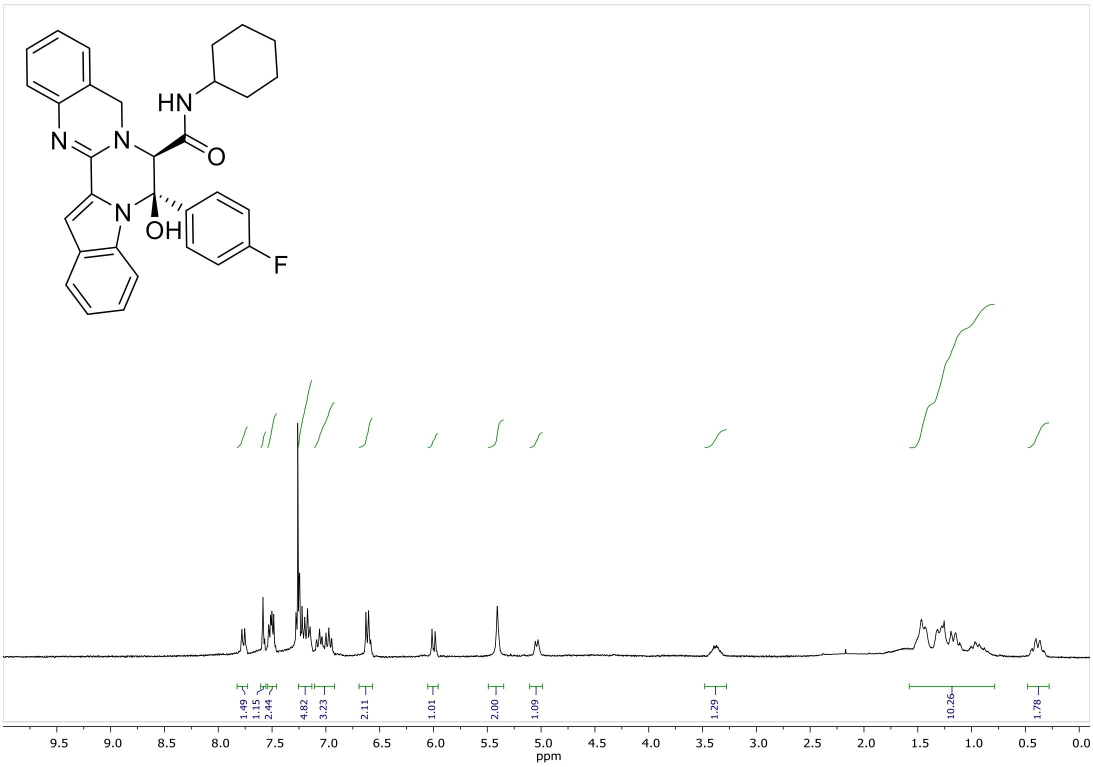

Figure S73. ^{1}H NMR spectrum (300 MHz, $CDCl_3$).

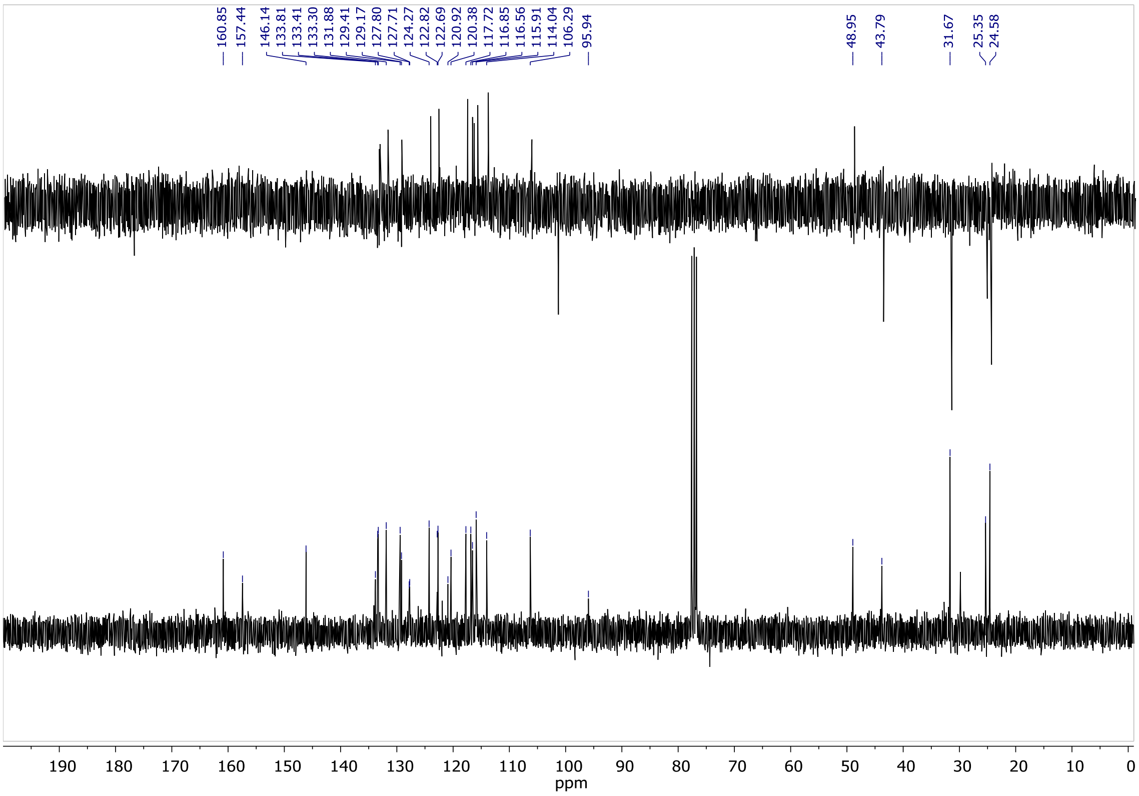

Figure S74. $^{13}C\{^1H\}$ and DEPT-135 NMR spectra (75 MHz, $CDCl_3$).

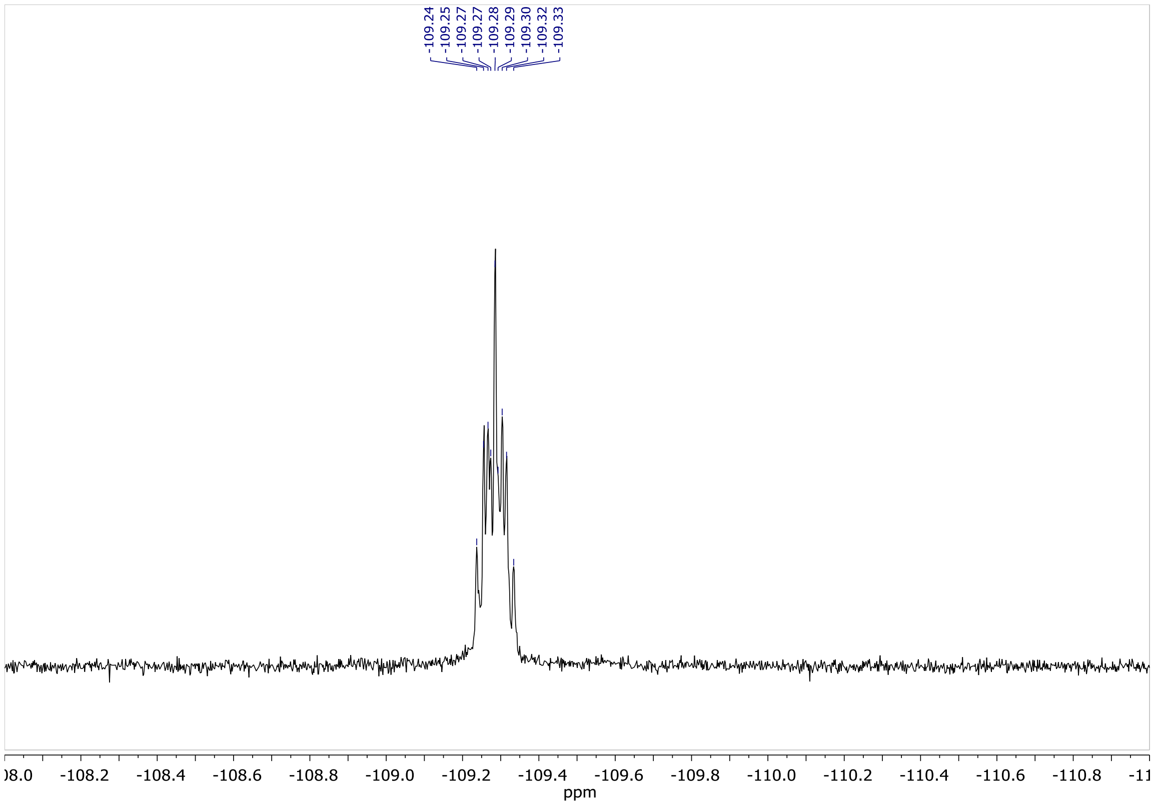

Figure S75. ^{19}F NMR spectrum (282 MHz, $CDCl_3$).

(6*R,7*R**)-*N*-(*tert*-Butyl)-6-hydroxy-6-(4-methoxyphenyl)-6,7-dihydro-9*H*-indolo[2',1':3,4]pyrazino[2,1-*b*]quinazoline-7-carboxamide. (13e)**

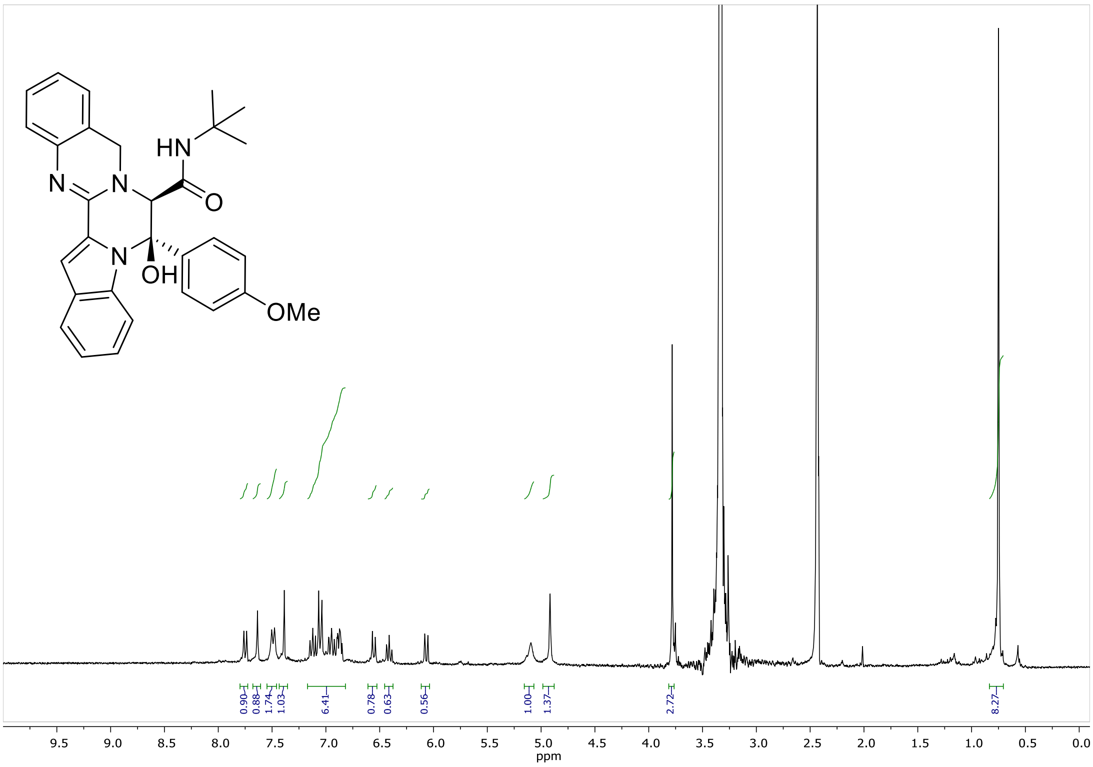

Figure S76. ^{1}H NMR spectrum (300 MHz, DMSO-d_6).

(6*R,7*R**)-*N*-(*tert*-Butyl)-6-hydroxy-6-(4-(trifluoromethyl)phenyl)-6,7-dihydro-9*H*-indolo[2',1':3,4]pyrazino[2,1-*b*]quinazoline-7-carboxamide. (13f)**

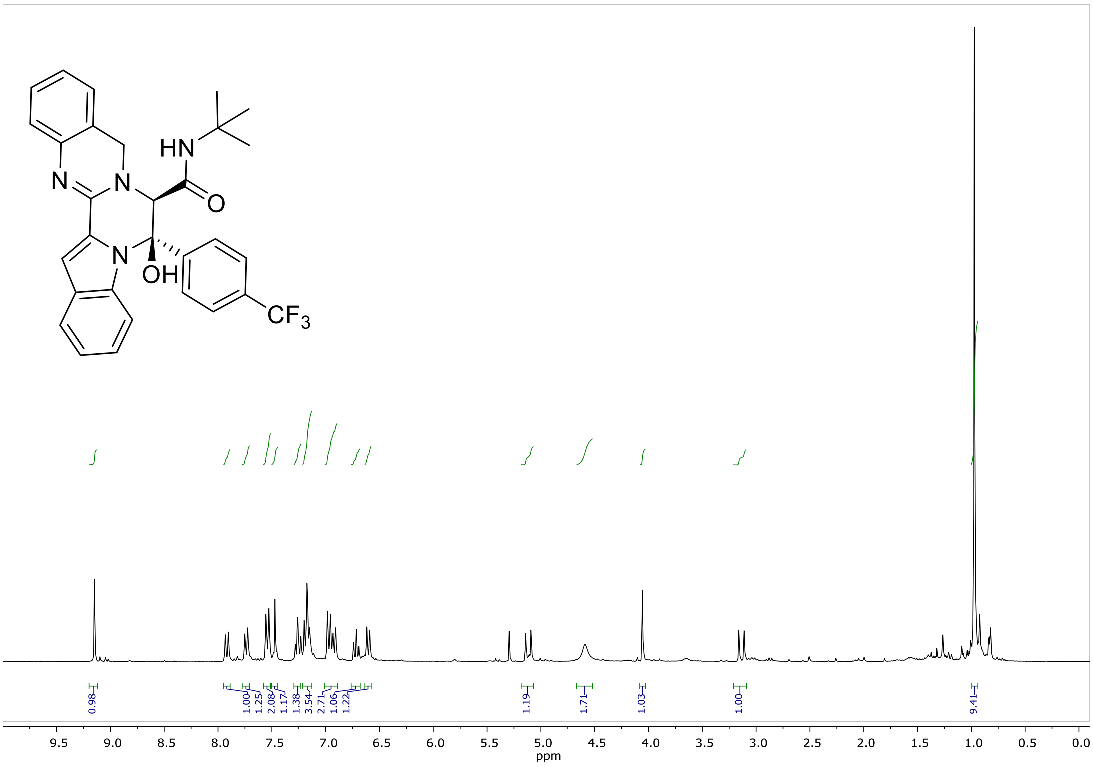

Figure S77. ^{1}H NMR spectrum (300 MHz, $CDCl_3$).

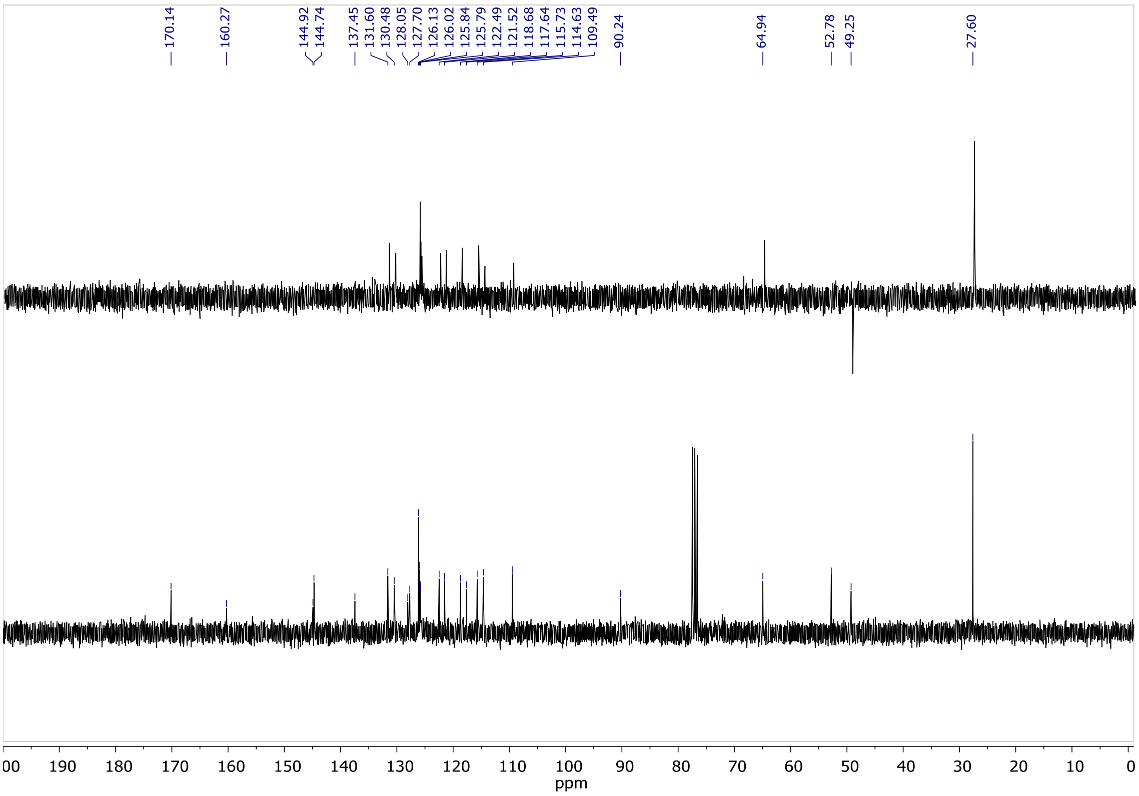

Figure S78. $^{13}C\{^{1}H\}$ and DEPT-135 NMR spectra (75 MHz, $CDCl_3$).

***N*-Cyclohexyl-2,3-diphenyl-1,6-dihydro-*2H*-pyrazino[2,1-*b*]quinazoline-4-carboxamide. (14)**

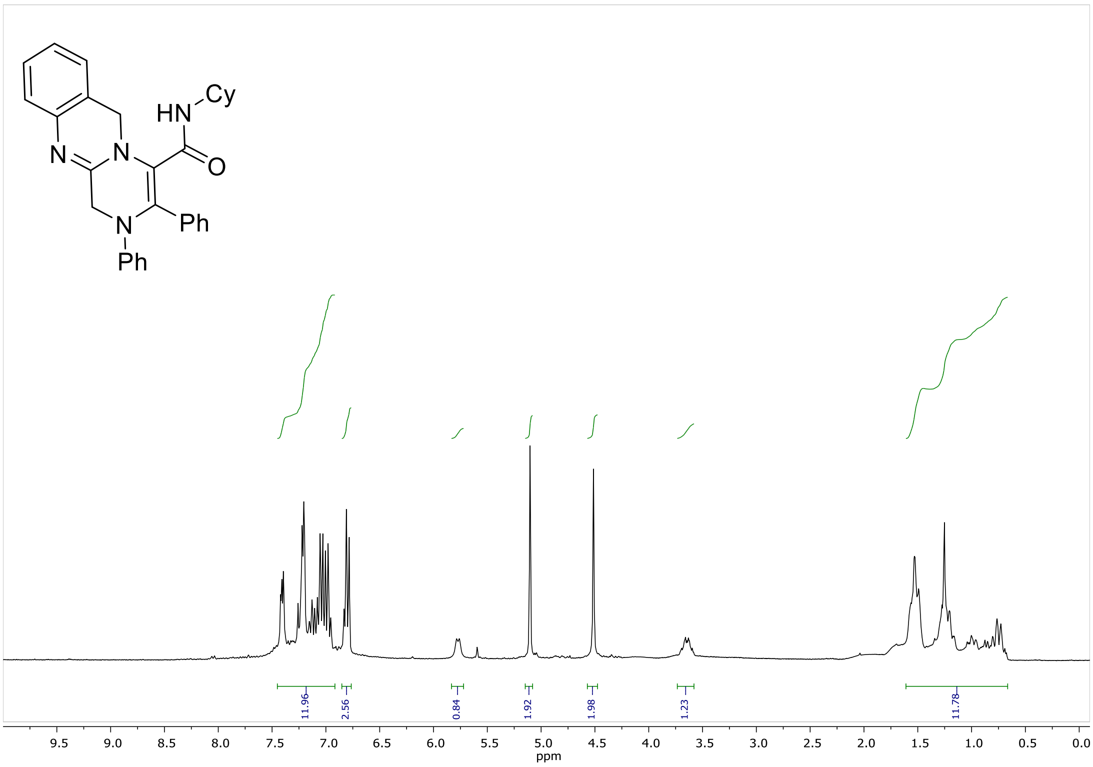

Figure S79. ^{1}H NMR spectrum (300 MHz, $CDCl_3$).

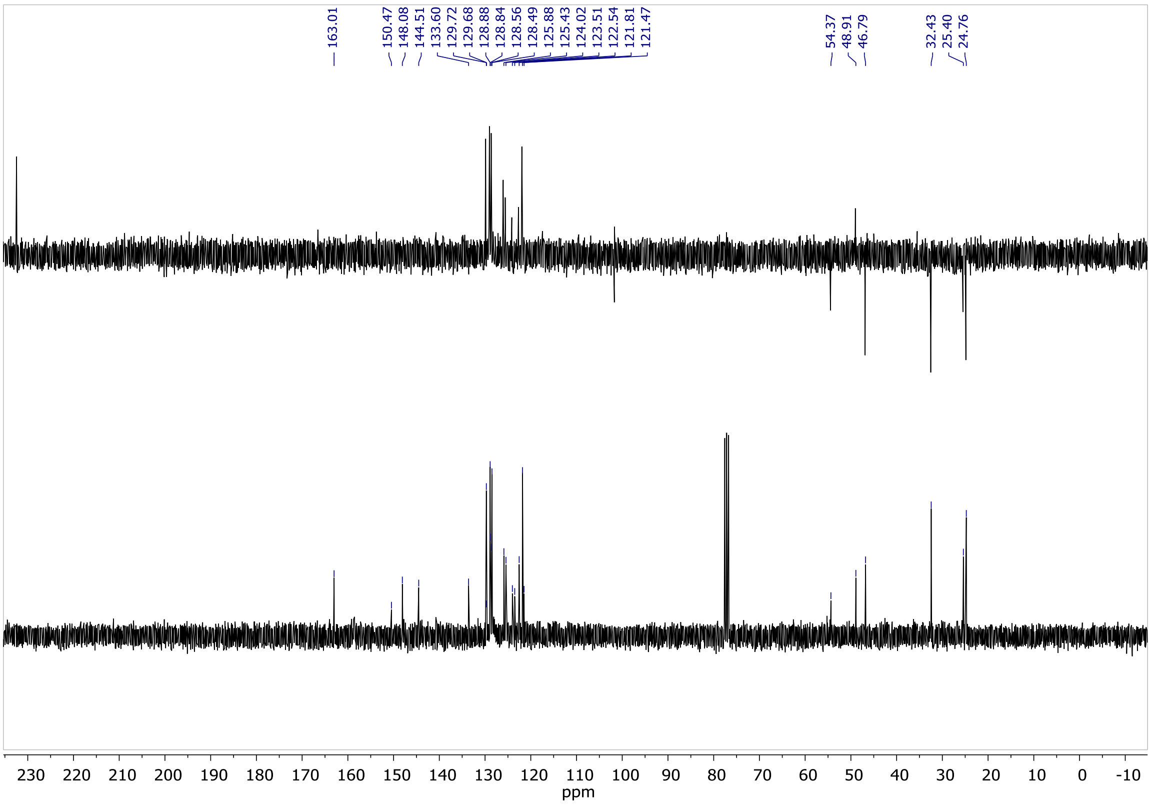

Figure S80. $^{13}C\{^{1}H\}$ and DEPT-135 NMR spectra (75 MHz, $CDCl_3$).

(10*R,10*aR**)-*N*-Cyclohexyl-10-hydroxy-5-oxo-10-phenyl-2,3-dihydro-1*H*,5*H*-dipyrrolo[1,2-*a*:1',2'-*d*]pyrazine-10a(10*H*)-carboxamide. (12a)**

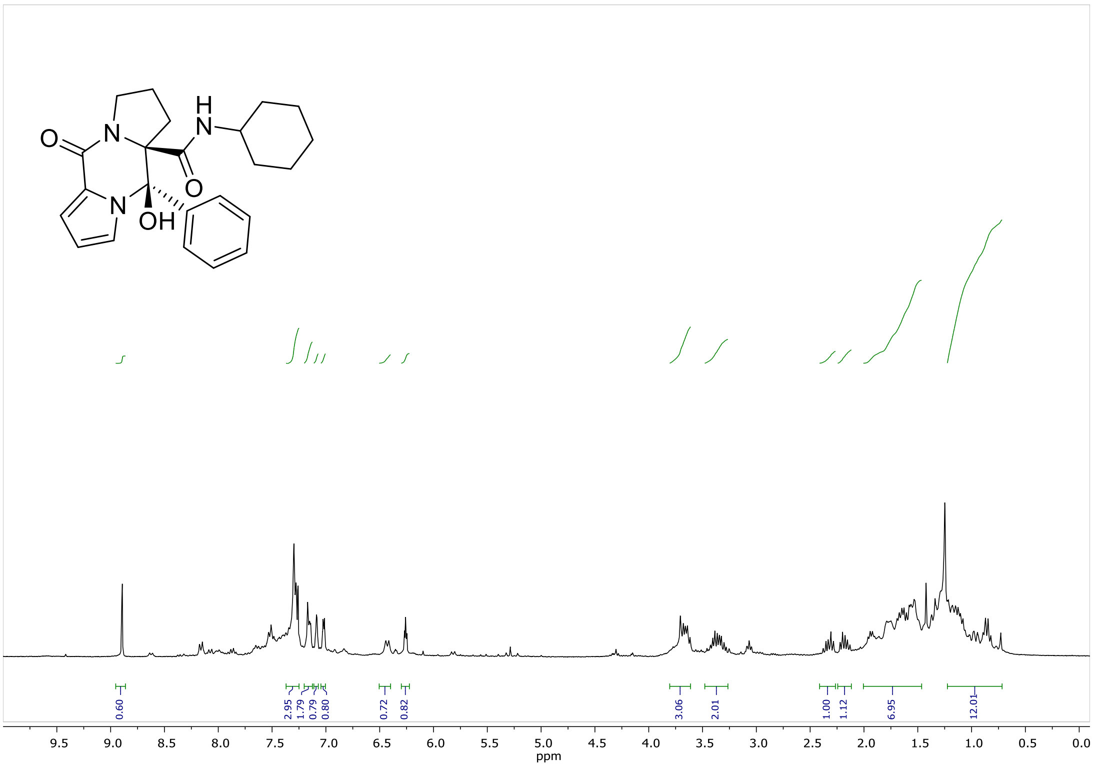

Figure S81. ^{1}H NMR spectrum (300 MHz, $CDCl_3$).

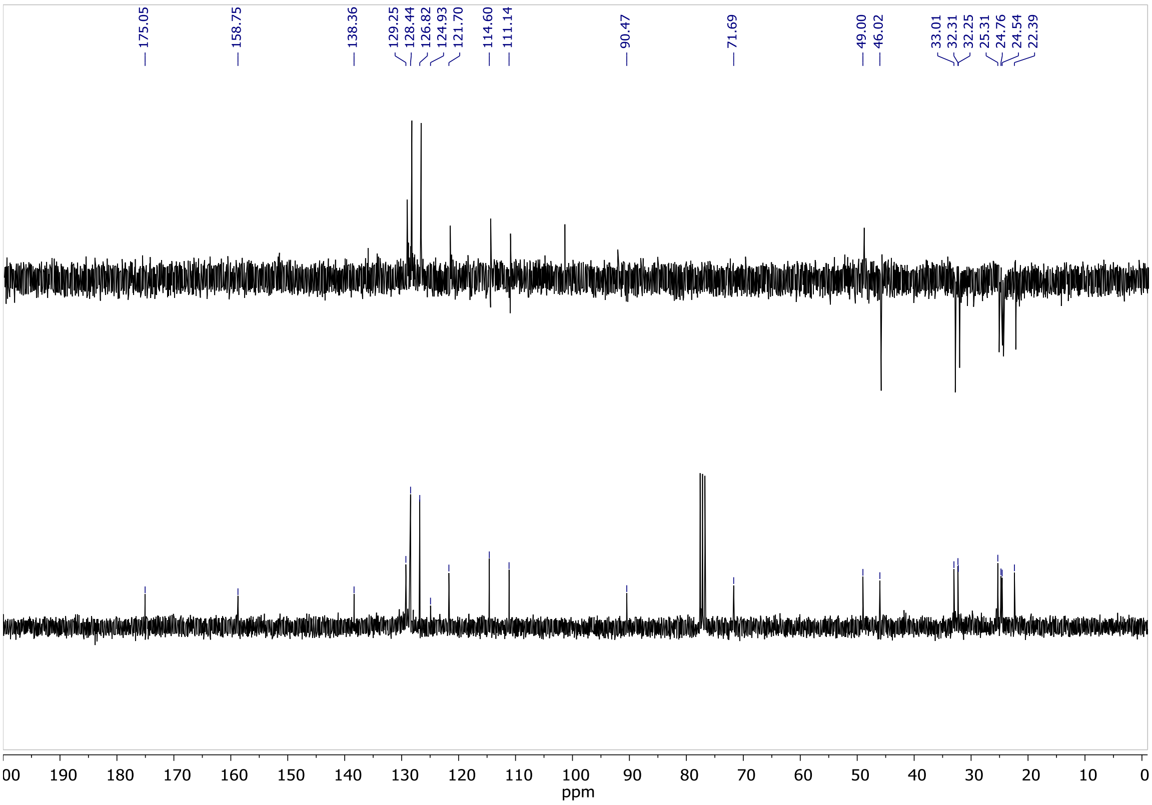

Figure S82. $^{13}C\{^{1}H\}$ and DEPT-135 NMR spectra (75 MHz, $CDCl_3$).

(10*R,10a*R**)-*N*-(*tert*-Butyl)-10-hydroxy-5-oxo-10-phenyl-2,3-dihydro-1*H*,5*H*-dipyrrolo[1,2-*a*:1',2'-*d*]pyrazine-10a(10*H*)-carboxamide. (12b)**

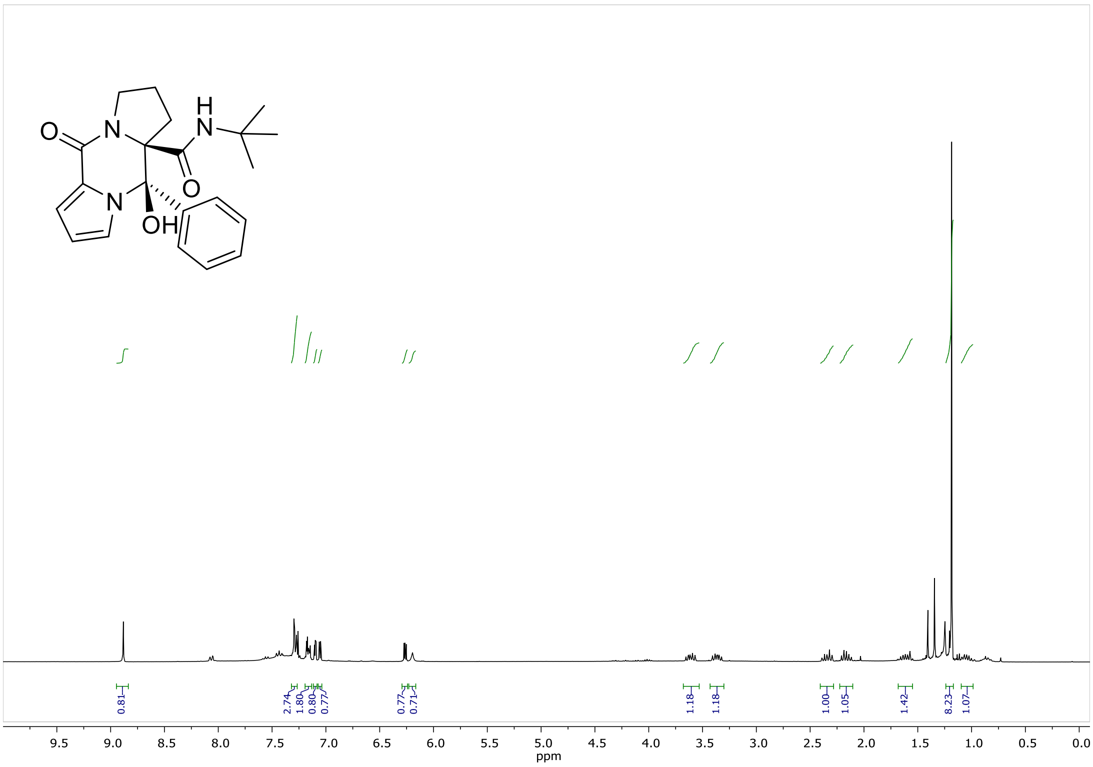

Figure S83. ^{1}H NMR spectrum (300 MHz, $CDCl_3$).

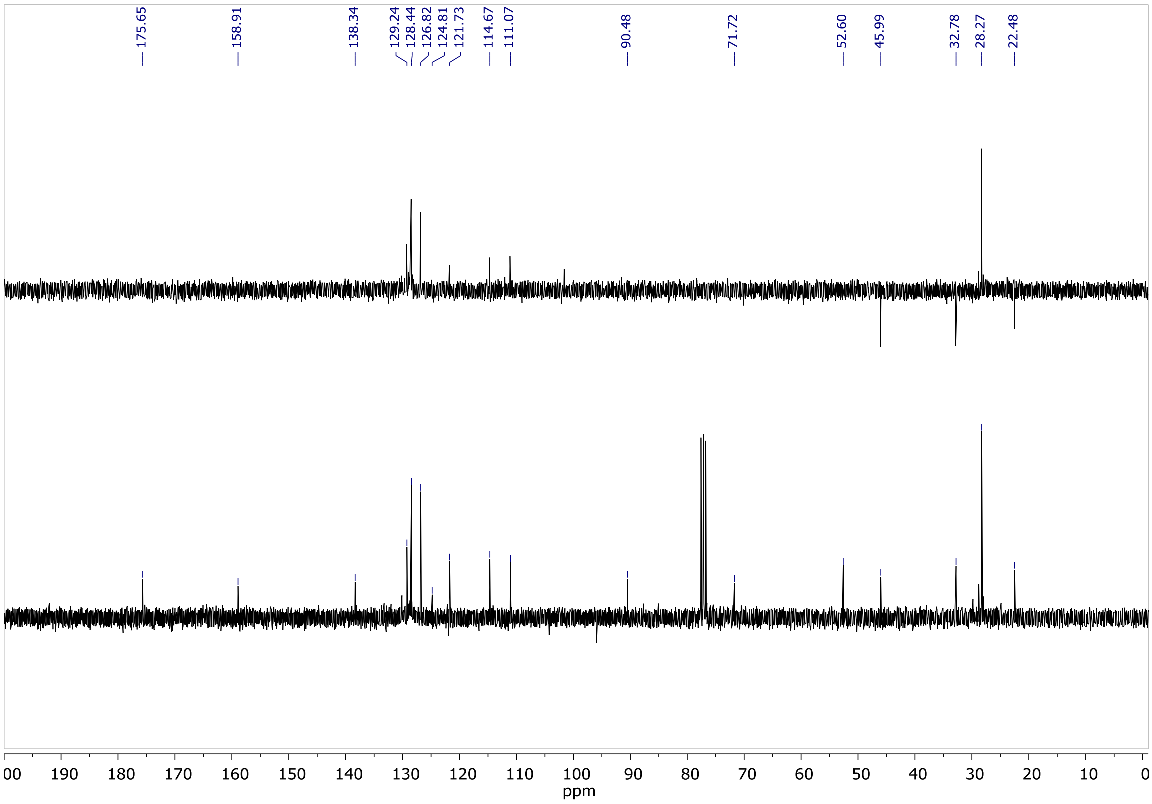

Figure S84. $^{13}C\{^{1}H\}$ and DEPT-135 NMR spectra (75 MHz, $CDCl_3$).

(10*R,10a*R**)-*N*-(*tert*-Butyl)-10-hydroxy-5-oxo-10-(4-fluorophenyl)-2,3-dihydro-1*H*,5*H*-dipyrrolo[1,2-*a*:1',2'-*d*]pyrazine-10a(10*H*)-carboxamide. (12c)**

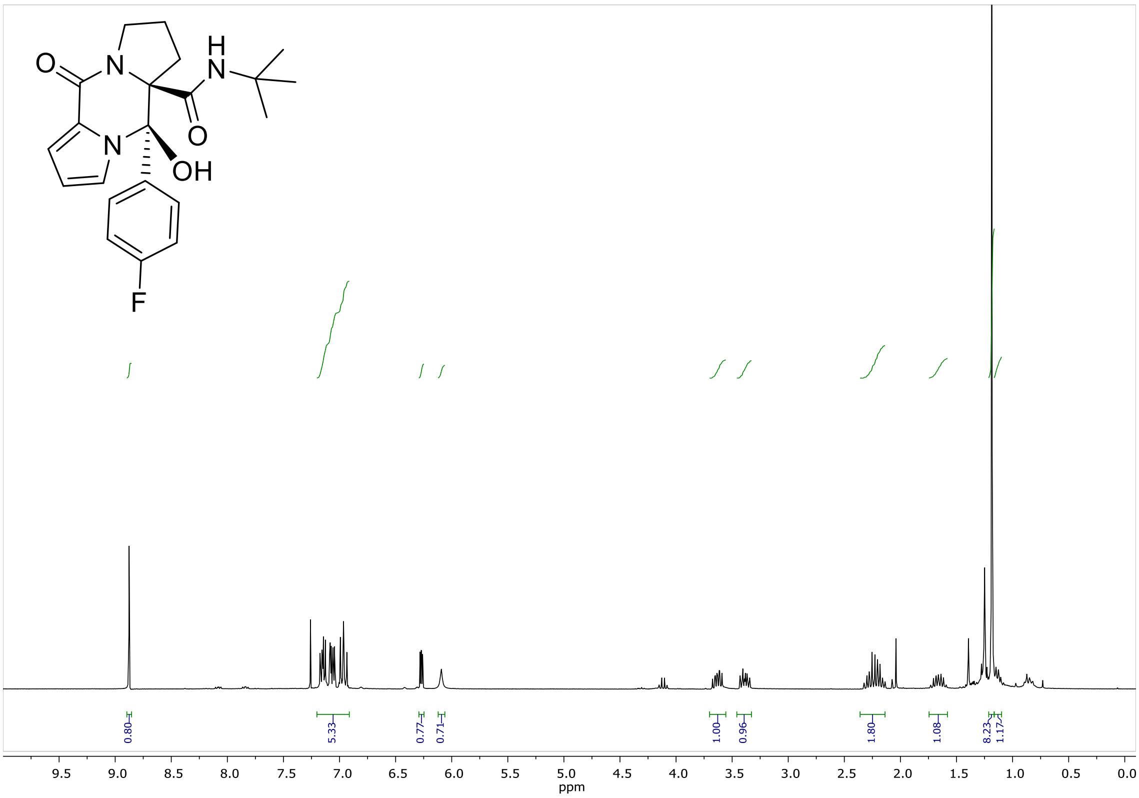

Figure S85. ^{1}H NMR spectrum (300 MHz, $CDCl_3$).

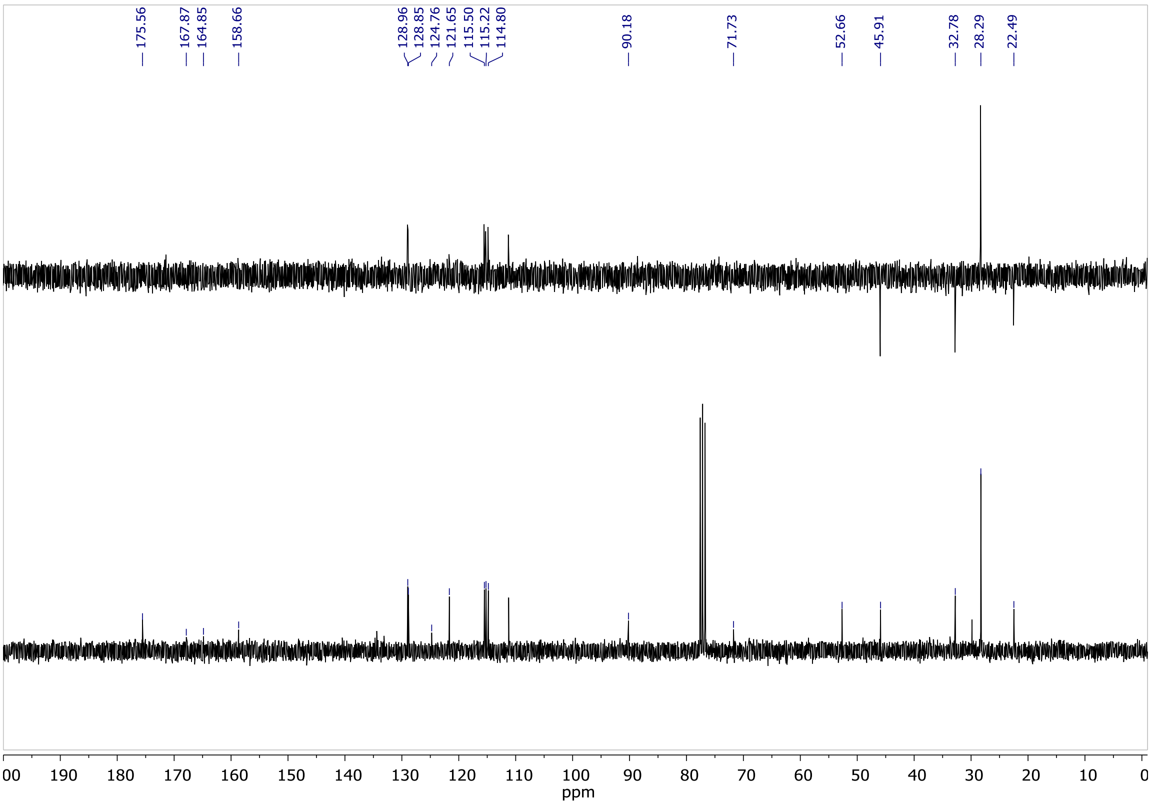

Figure S86. $^{13}C\{^{1}H\}$ and DEPT-135 NMR spectra (75 MHz, $CDCl_3$).

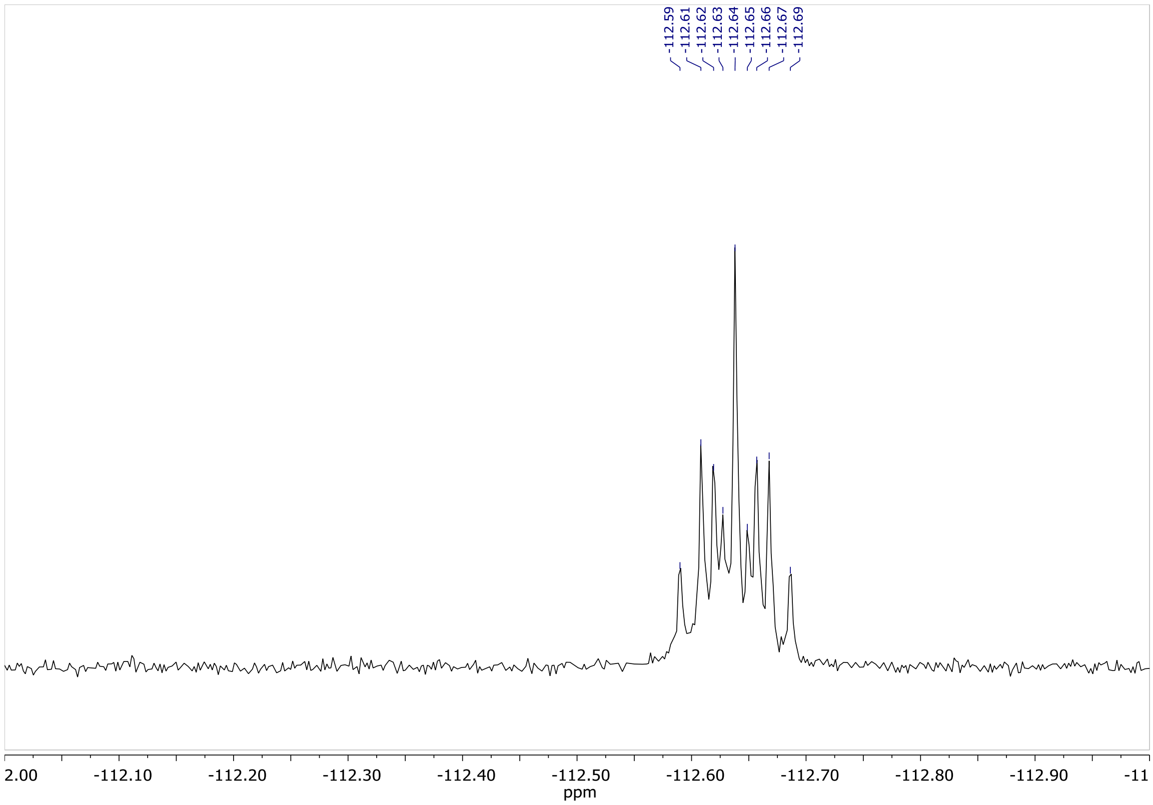

Figure S87. ^{19}F NMR spectrum (282 MHz, $CDCl_3$).

(12*R,12*aR**)-*N*-(*tert*-Butyl)-12-hydroxy-5-oxo-12-phenyl-2,3-dihydro-1*H*,5*H*-pyrrolo[1',2':4,5]pyrazino[1,2-*a*]indole-12a(12*H*)-carboxamide. (12d)**

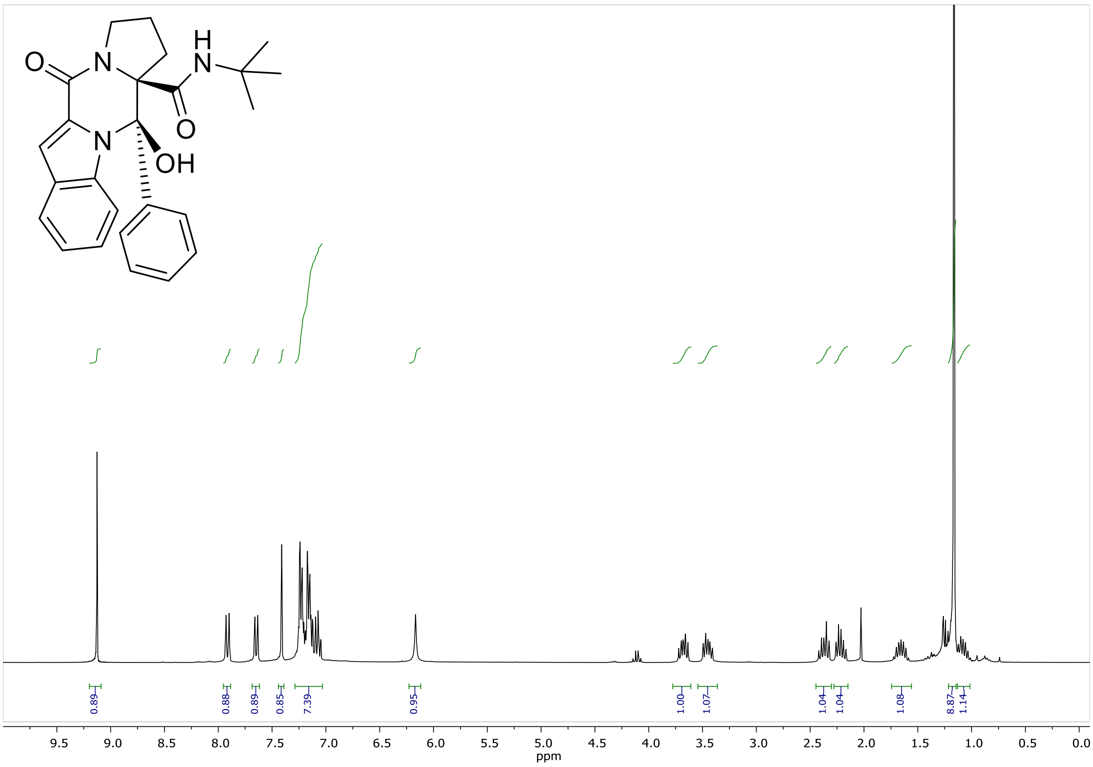

Figure S88. ^{1}H NMR spectrum (300 MHz, $CDCl_3$).

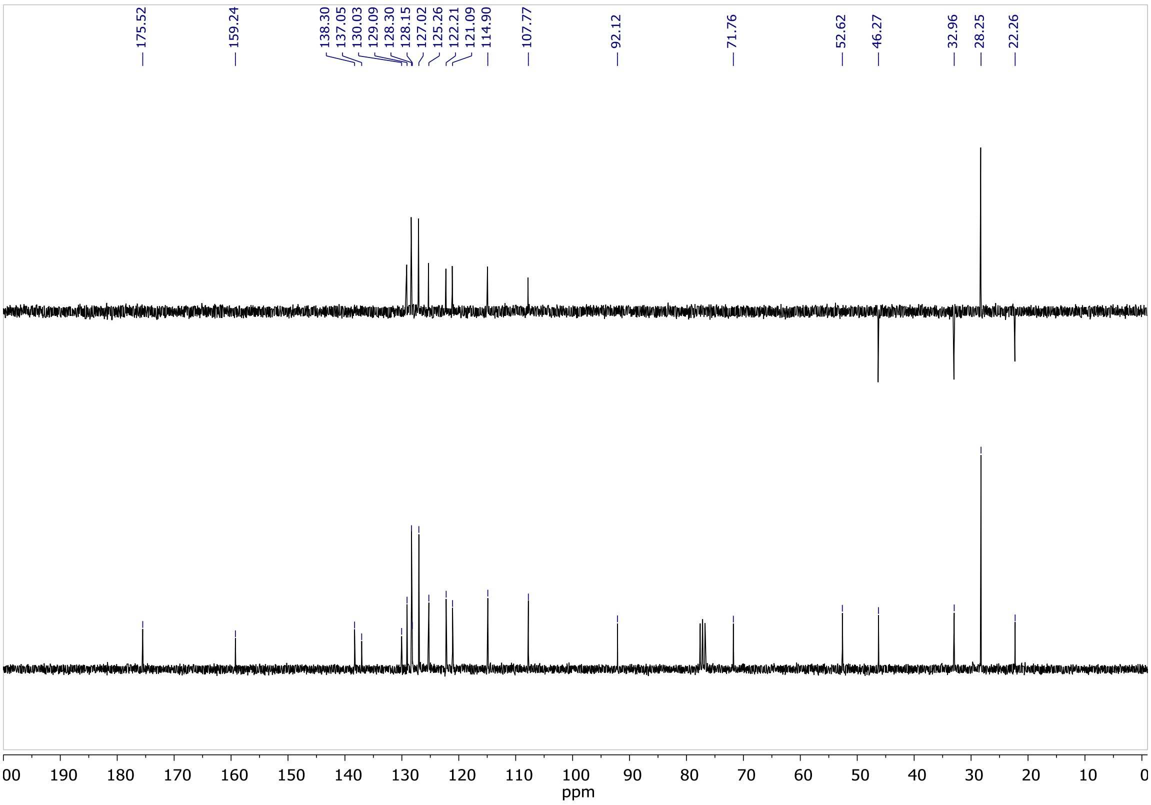

Figure S89. $^{13}C\{^{1}H\}$ and DEPT-135 NMR spectra (75 MHz, $CDCl_3$).
